# In vitro and in silico studies of 7′′,8′′-buddlenol D anti-inflammatory lignans from *Carallia brachiata* as p38 MAP kinase inhibitors

**DOI:** 10.1038/s41598-023-30475-5

**Published:** 2023-03-02

**Authors:** Nonthaneth Nalinratana, Utid Suriya, Chanyanuch Laprasert, Nakuntwalai Wisidsri, Preeyaporn Poldorn, Thanyada Rungrotmongkol, Wacharee Limpanasitthikul, Ho-Cheng Wu, Hsun-Shuo Chang, Chaisak Chansriniyom

**Affiliations:** 1 Department of Pharmacology and Physiology, Faculty of Pharmaceutical Sciences, Chulalongkorn University, Bangkok, 10330 Thailand; 2 Program in Biotechnology, Faculty of Science, Chulalongkorn University, Bangkok, 10330 Thailand; 3 Department of Pharmacology, Faculty of Medicine, Chulalongkorn University, Bangkok, 10330 Thailand; 4 Faculty of Integrative Medicine, Rajamangala University of Technology Thanyaburi, Pathum Thani, 12130 Thailand; 5 Biocatalyst and Environmental Biotechnology Research Unit, Department of Biochemistry, Faculty of Science, Chulalongkorn University, Bangkok, 10330 Thailand; 6 Program in Bioinformatics and Computational Biology, Graduate School, Chulalongkorn University, Bangkok, 10330 Thailand; 7 Graduate Institute of Pharmacognosy, College of Pharmacy, Taipei Medical University, Taipei, 110 Taiwan; 8 School of Pharmacy, College of Pharmacy, Kaohsiung Medical University, Kaohsiung, 807 Taiwan; 9 Department of Pharmacognosy and Pharmaceutical Botany, Faculty of Pharmaceutical Sciences, Chulalongkorn University, Bangkok, 10330 Thailand; 10 Natural Products and Nanoparticles Research Unit, Chulalongkorn University, Bangkok, 10330 Thailand

**Keywords:** Cell biology, Drug discovery, Molecular biology

## Abstract

Excessive macrophage activation induces the release of high levels of inflammatory mediators which not only amplify chronic inflammation and degenerative diseases but also exacerbate fever and retard wound healing. To identify anti-inflammatory molecules, we examined *Carallia brachiata*—a medicinal terrestrial plant from Rhizophoraceae. Furofuran lignans [(−)-(7′′*R*,8′′*S*)-buddlenol D (**1**) and (−)-(7′′*S*,8′′*S*)-buddlenol D (**2**)] isolated from the stem and bark inhibited nitric oxide (half maximal inhibitory concentration (IC_50_): 9.25 ± 2.69 and 8.43 ± 1.20 micromolar for **1** and **2**, respectively) and prostaglandin E_2_ (IC_50_: 6.15 ± 0.39 and 5.70 ± 0.97 micromolar for **1** and **2**, respectively) productions in lipopolysaccharide-induced RAW264.7 cells. From western blotting, **1** and **2** suppressed LPS-induced inducible nitric oxide synthase and cyclooxygenase-2 expression in a dose-dependent manner (0.3–30 micromolar). Moreover, analysis of the mitogen-activated protein kinase (MAPK) signaling pathway showed decreased p38 phosphorylation levels in **1**- and **2**-treated cells, while phosphorylated ERK1/2 and JNK levels were unaffected. This discovery agreed with in silico studies which suggested **1** and **2** bound to the ATP-binding site in p38-alpha MAPK based on predicted binding affinity and intermolecular interaction docking. In summary, 7′′,8′′-buddlenol D epimers demonstrated anti-inflammatory activities via p38 MAPK inhibition and may be used as viable anti-inflammatory therapies.

## Introduction

Inflammation is a cascade event generated by innate immunity responses against microbial infection^1^. Additionally, non-infectious stimuli such as cell damage and tissue injury also trigger these responses and lead to local inflammation. Uncontrolled acute inflammation gradually destroys tissues and organs, diminishes their functions, and evolves into chronic inflammation and degenerative diseases such as osteoarthritis, rheumatoid arthritis, and even cancer^2^. When exposed to chemoattractants, tissue monocytes evolve to macrophages which engulf and destroy microbes in phagolysosomes using various hydrolytic enzymes and substances, including nitric oxide (NO). Moreover, NO is released into the extracellular fluid and functions as an inflammatory mediator which enhances phagocyte migration to injured or infected sites to amplify immune responses. However, excessive NO production limits recovery processes and destroys surrounding tissues^3,4^. Toll-like receptors (TLRs), especially TLR-4 which is a pathogen recognition receptor, and mitogen-activated protein kinase (MAPK) signaling pathways have important roles during inflammatory responses. Their deactivation mitigates acute sepsis, chronic inflammation, and degenerative diseases^5,6^. Eritoran, a structural lipopolysaccharide (LPS) lipid A mimic, and SB203580, a pyridinylimidazole derivative, both target TLR-4 and p38 MAPK, respectively. Additionally, curcumin and its analog L48H37 are effective in inhibiting TLR-4^7,8^. Furthermore, p38 MAPK inhibition is beneficial for Alzheimer’s disease treatment^9^. Natural compounds such as icariin, apigenin, and astaxanthin also decrease amyloid-β-induced neurotoxicity via p38 MAPK inhibition^9^.

Natural products are vital sources of medicines and have been used extensively by different populations; in Thailand, traditional medicines have been used for centuries. Based on Thai traditional medicine, *Carallia brachiata* (Lour.) Merr. (Rhizophoraceae) is one such natural source whose stem and bark are used for antipyretic remedies. Similarly, in Ayurveda (traditional Hindu medicine system) the stem bark is also used to treat oral ulcers and stomatitis^10^. In a previous study, ethyl acetate (EtOAc) and methanol (MeOH) bark extracts showed significant wound healing properties in incision and excision wound rat models^11^. Furthermore, MeOH and hydro-ethanolic leaf extracts exhibited analgesic, anti-inflammatory, and anti-diabetic activities in in vivo models^12,13^. Based on these traditional uses and pharmacological activities, the anti-inflammatory activity of this plant appears to underlie its mechanism of action.

In terms of chemical investigations, two pyrrolidine alkaloids [hygroline and (+)-pseudohygroline], a megastigmane diglycoside [3-hydroxy-5,6-epoxy-β-ionol-3-*O*-β*-*apiofuranosyl-(1 → 6)-β*-*glucopyranoside], and flavonoid glycosides such as apigenin-7-*O*-α-rhamnoside-(1 → 2)-β*-*glucopyranoside were isolated from leaves of *C. brachiata*^14–16^. Also, *p*-hydroxy benzoic acid and two proanthocyanidins [carallidin and mahuanin A], which possessed anti-radical scavenging activities against 2,2-diphenyl-1-picrylhydrazyl (DPPH) and superoxide radicals and inhibited xanthine oxidase, were isolated from bark^17^. To the best of our knowledge, no evidence exists in the literature to support the compounds contributing to the activities addressed in traditional uses of this plants, especially for anti-inflammatory activity which is linked antipyretic and wound healing properties. In this work, we identified compounds which exerted anti-inflammatory activities, examined their effects on p38 MAPK signaling pathway, and developed in silico models to identify ligand binding targets.

## Results

### Compounds isolated from *C. brachiata* stem and bark

**Figure 1:**
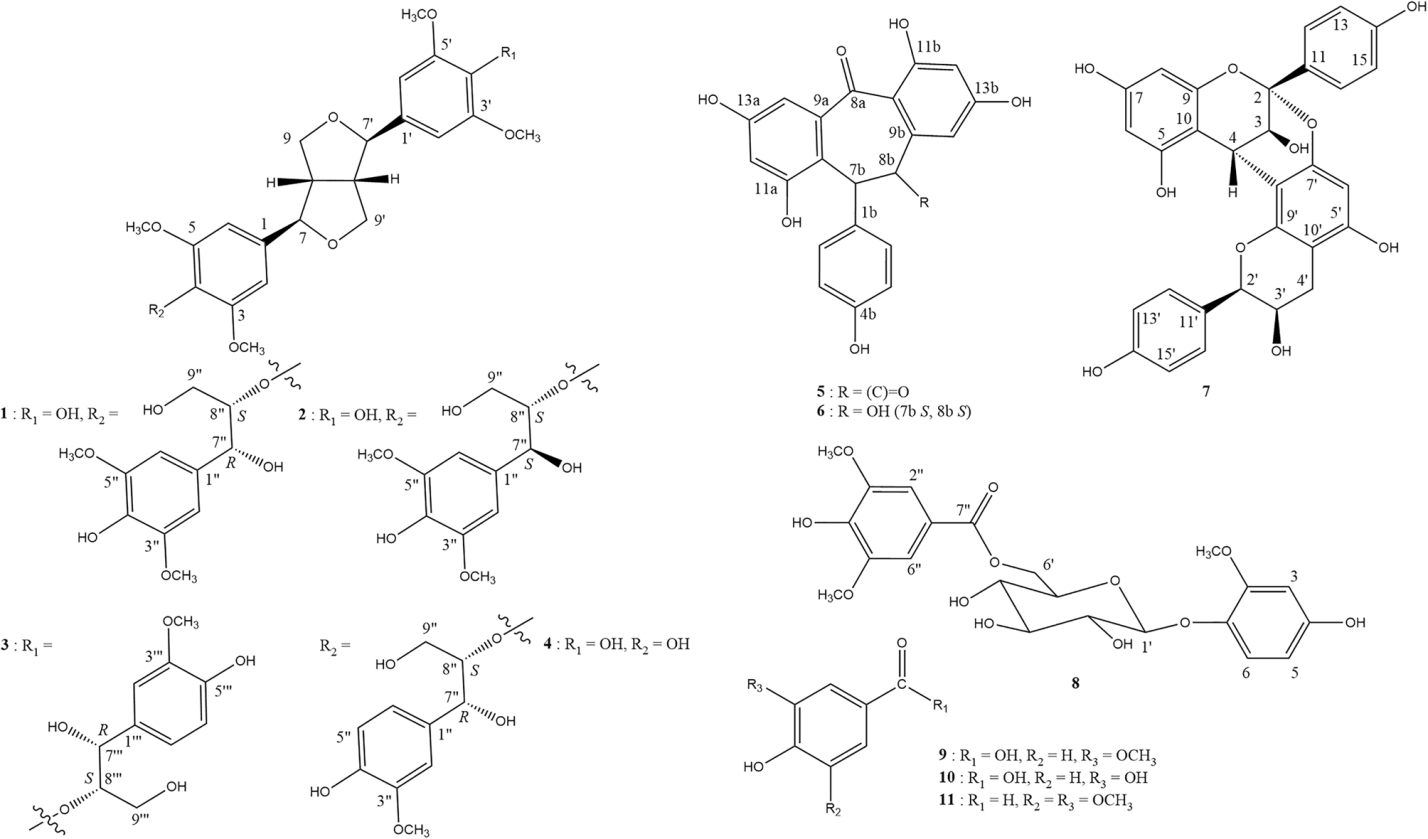
Compounds isolated from EtOAc extract of *C. brachiata* stem and bark. (−)-(7′′*R*,8′′*S*)-buddlenol D (**1**), (−)-(7′′*S*,8′′*S*)-buddlenol D (**2**), (+)-7″*R*,8′′*S*:7″′*R*,8′′′*S*-hedyotisol A (**3**), (−)-syringaresinol (**4**), (+)-diptoindonesin D (**5**), (+)-parviflorol (**6**), (−)-mahuanin A (**7**), 6-*O*-syringoyl-β-d-glucopyranoside derivative (**8**), vanillin (**9**), protocatechuic acid (**10**), and syringaldehyde (**11**).

Eleven compounds were isolated from stem and bark*.* Of these, two sesquineolignans [(−)-(7′′*R*,8′′*S*)-buddlenol D (**1**) and (−)-(7′′*S*,8′′*S*)- buddlenol D (**2**)], together with (+)-7″*R*,8′′*S*:7″′*R*,8′′′*S*-hedyotisol A (**3**), (−)-syringaresinol (**4**), two modified stilbene dimers [(+)-diptoindonesin D (**5**) and (+)-parviflorol (**6**)], a proanthrocyanidin [(−)-mahuanin A (**7**)], 4-hydroxy-2-methoxyphenyl-6-*O*-syringoyl-β-D-glucopyranoside (**8**), and three benzoic acid derivatives [vanillic acid (**9**), protocatechuic acid (**10**), and syringaldehyde (**11**)] were identified by spectroscopic analysis and compared with the literature (Fig. 1). On silica gel GF254 thin-layer chromatography (TLC) plate, **1** and** 2** were observed at retardation factor (Rf) values of 0.44 and 0.48, respectively, when triply developed in 20% acetone/dichloromethane (Me_2_CO/CH_2_Cl_2_) (Supplementary Information: Figure S1).

### (−)-(7′′***R***,8′′***S***)-Buddlenol D (1) and (−)-(7′′***S***,8′′***S***)-buddlenol D (2) inhibit LPS-stimulated NO and PEG_2_ expression in RAW264.7 cells

**Figure 2:**
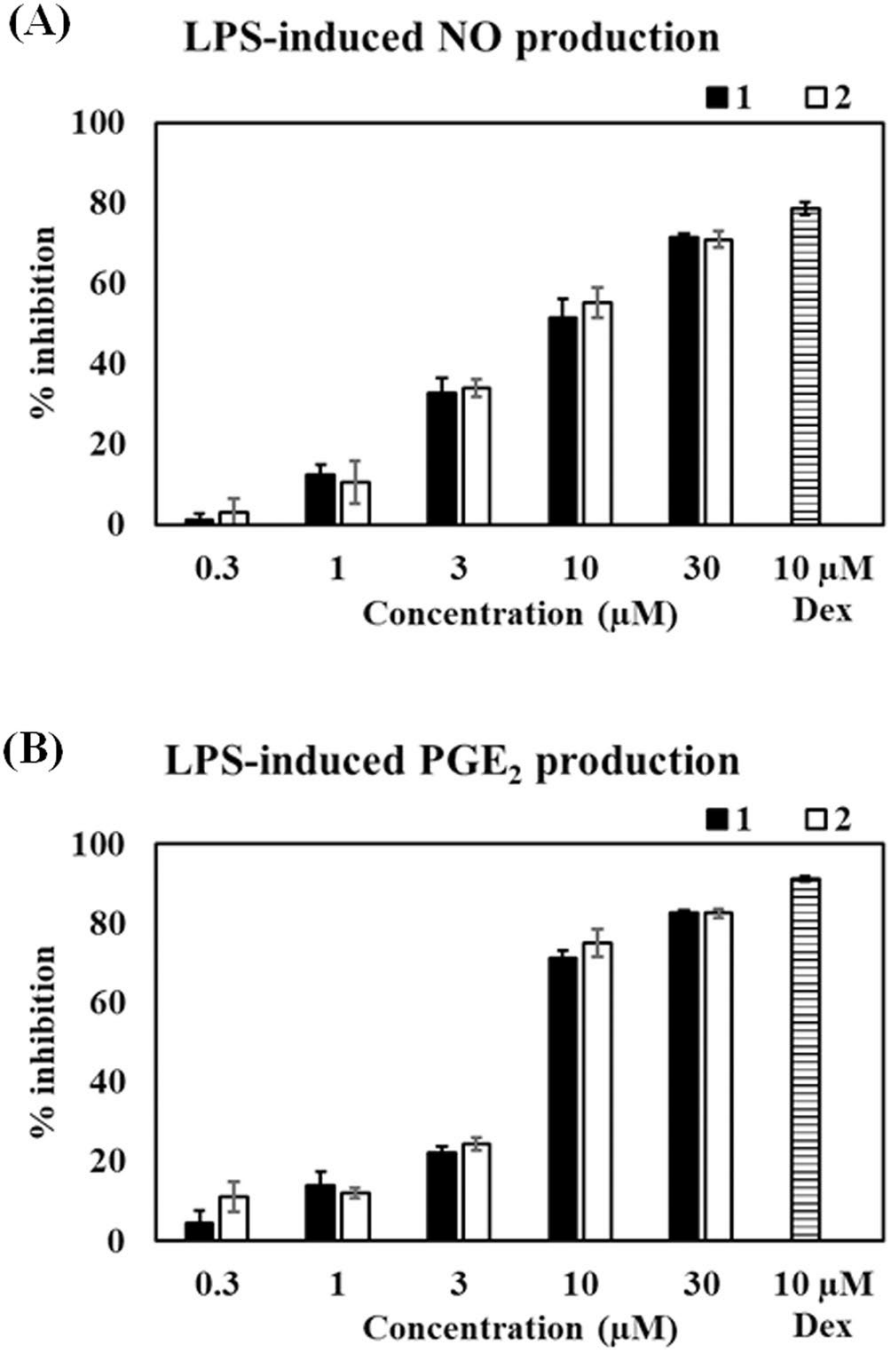
The inhibitory effects of **1** and **2** on (**A**) nitric oxide (NO) and (**B**) prostaglandin E_2_ (PGE_2_) production in lipopolysaccharide (LPS)-induced RAW264.7 macrophages. Dexamethasone was used as a positive control. Percentage inhibition was calculated using the LPS-induced group as a control. Data were represented as the mean ± standard error of the mean from three independent experiments (n = 3).

To determine the anti-inflammatory activity of *C. brachiata* stem and bark, the crude MeOH and its partitioned extracts were preliminary tested for NO inhibition in murine macrophages (RAW264.7). The EtOAc extract was selected for further isolation. All isolates were screened for NO inhibition at a final concentration of 30 μM. Only buddlenols D** 1** and **2**, which exhibited > 50% inhibition, underwent half maximal inhibitory concentrations (IC_50_) analysis. In pre-treated RAW264.7 cells with **1** and **2** prior to stimulation with 500 ng/mL LPS, **1** and **2** inhibited NO production in a concentration-dependent manner (0.3–30 µM) and exhibited no cytotoxicity (Supplementary Information: Figure S5), giving calculated IC_50_ values of 9.25 ± 2.69 and 8.43 ± 1.20 µM, respectively. Dexamethasone (10 µM) was used as a positive control showed percentage of NO inhibition at 78.69 ± 1.48% (Fig. 2).

LPS also stimulates prostaglandin E_2_ (PGE_2_) production via the arachidonic acid pathway. Buddlenols D **1** and **2** were evaluated for PGE_2_ inhibition as a putative fever suppressant. Cell pretreatment with **1** and **2** inhibited LPS-induced PGE_2_ production in a concentration-dependent manner; IC_50_ values for **1** and **2** were 6.15 ± 0.39 and 5.70 ± 0.97 µM, respectively, whereas dexamethasone (10 µM) exhibited an inhibition percentage of 91.21 ± 1.17% (Fig. 2).

### (−)-(7′′***R***,8′′***S***)-Buddlenol D (1) and (−)-(7′′***S***,8′′***S***)-buddlenol D (2) inhibit LPS-induced inducible nitric oxide synthase (iNOS) and cyclooxygenase-2 (COX2) expression

**Figure 3:**
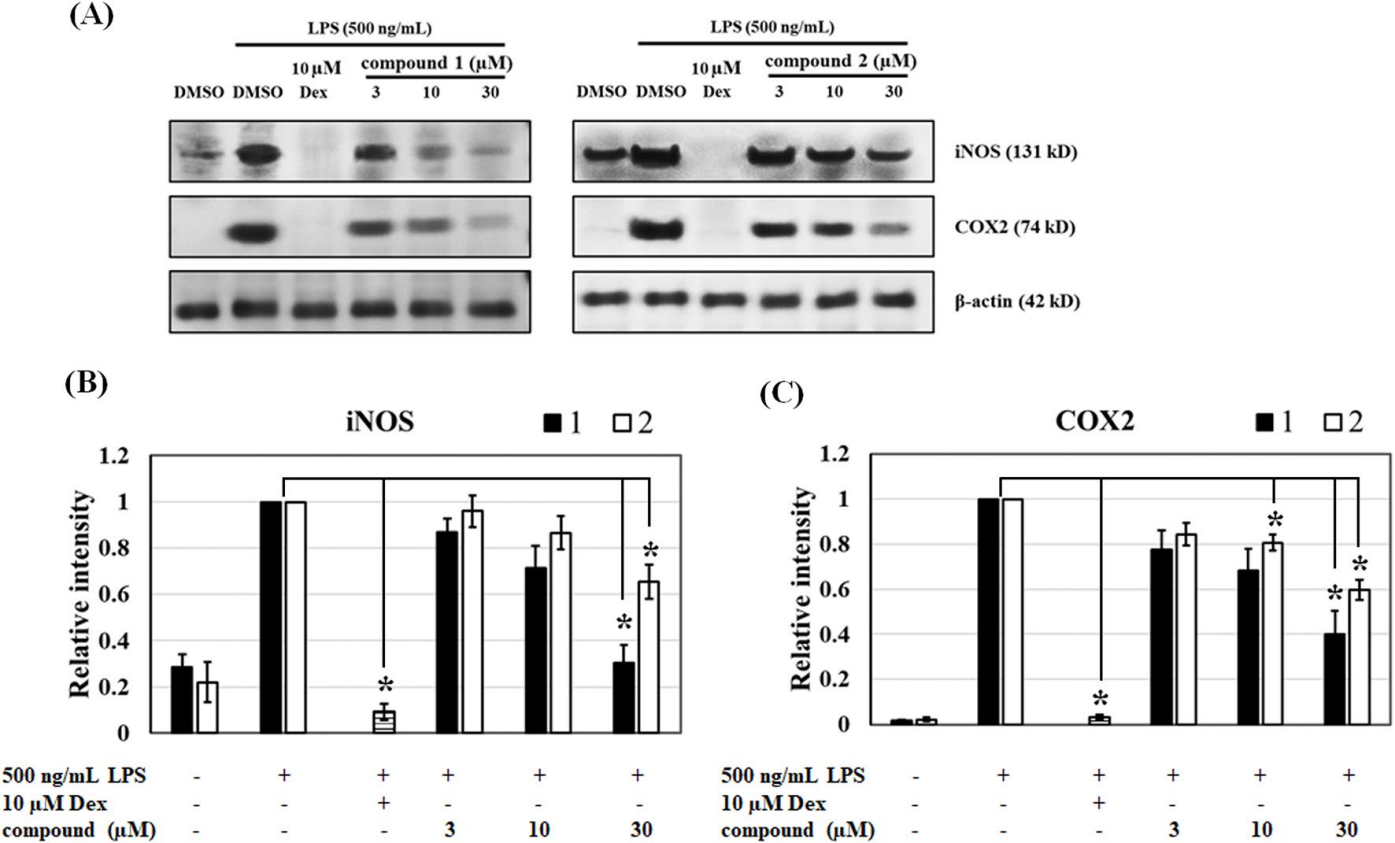
The effects of **1** and **2** on inducible nitric oxide synthase (iNOS) and cyclooxygenase 2 (COX2) protein expression. (**A**) Representative immunoblots showing LPS-induced RAW264.7 macrophages treated with **1** and **2**. The cropped blots were used in the figure. The protein lysates of each treatment group from the same experiment were performed simultaneously on the same gel for each protein detection. Full-length uncropped blots were shown in supplementary information data (Figure S59). Densitometric histograms showing (**B**) iNOS and (**C**) COX2 in LPS-induced RAW264.7 macrophages. Dexamethasone was used as a positive control. Data were expressed as relative intensity when compared with LPS-induced groups and were represented as the mean ± standard error of the mean from three independent experiments (n = 3). *p < 0.05 vs. the LPS-induced group.

Since buddlenols D **1** and **2** suppressed NO and PGE_2_ production, their effects on iNOS and COX2 protein expression were evaluated by western blotting. RAW264.7 cells treated with 500 ng/mL LPS significantly increased iNOS and COX2 expression when compared with untreated cells. Cells pre-incubated with **1** and **2** markedly decreased iNOS and COX2 expression in LPS-stimulated cells in a concentration-dependent manner. Both iNOS and COX2 expression were more reduced in **1**-treated cells than **2**-treated cells, although the IC_50_ values for NO and PGE_2_ inhibition were not significantly different between groups. Dexamethasone (10 µM) almost completely ablated iNOS and COX2 expression (Fig. 3).

### (−)-(7′′***R***,8′′***S***)-Buddlenol D (1) and (−)-(7′′***S***,8′′***S***)-buddlenol D (2) suppress LPS-induced p38 but not ERK1/2 and JNK phosphorylation

**Figure 4:**
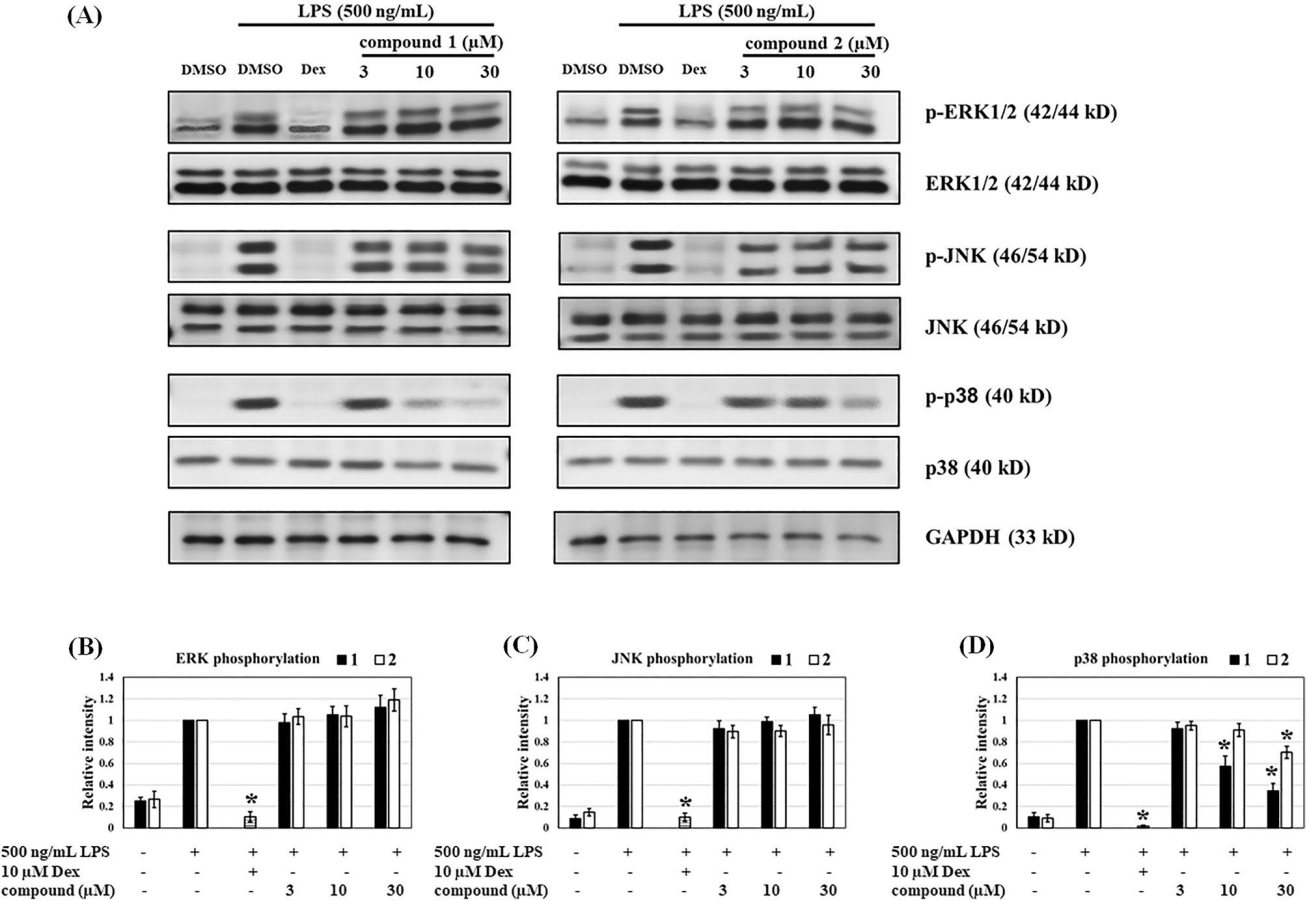
The effects of **1** and **2** on mitogen-activated protein kinase (MAPK) signaling elements. (**A**) Representative immunoblots showing LPS-induced RAW264.7 macrophages treated with **1** and **2**. The cropped blots were used in the figure. The protein lysates of each treatment group from the same experiment were performed simultaneously on the same gel for each protein detection. Full-length uncropped blots were shown in supplementary information data (Figure S60). Densitometric histograms showing (**B**) phosphorylated ERK (p-ERK), (**C**) phosphorylated JNK (p-JNK), and (**D**) phosphorylated p38 (p-38) in LPS-induced RAW264.7 macrophages. Dexamethasone was used as a positive control. Data were expressed as relative intensity compared with LPS-induced groups and represented as the mean ± standard error of the mean from three independent experiments (n = 3). *p < 0.05 vs. LPS-induced groups.

To investigate the possible intracellular mechanisms underpinning iNOS and COX2 inhibition by buddlenols D **1** and **2**, we evaluated their effects on p38, extracelluar signal-regulated protein kinase 1 and 2 (ERK1/2), and c-Jun N-terminal kinase (JNK) phosphorylation. Treatment with 500 ng/mL LPS significantly increased p-p38, p-ERK1/2, and p-JNK levels detected at 24-h incubation when compared with non-stimulated cells. Dexamethasone (10 µM) also significantly suppressed these levels when induced by LPS and suggested its anti-inflammatory activity may be related to the inhibition of p38, ERK1/2, and JNK phosphorylation. In cells pre-treated with buddlenols **1** and **2**, p38 phosphorylation was significantly reduced when compared with LPS-treated cells. However, ERK1/2 and JNK phosphorylation levels were not significantly changed. Therefore, the effects of **1** and **2** on NO and PGE_2_ suppression may be due to inhibited p38 phosphorylation (Fig. 4). In addition, the effect of a specific p38 inhibitor SB203580 on p38 kinase and NO inhibition was investigated. The results showed that SB203580 at 1 µM completely inhibited LPS-induced p38 phosphorylation and reduced NO production with an IC_50_ of 0.82 ± 0.05 µM, compared to IC_50_ values of 9.25 ± 2.69 and 8.43 ± 1.20 µM for **1** and **2**, respectively (Supplementary Information: Figure S6).

### The in silico prediction of ligand binding targets and ADMET calculation

Since buddlenols D **1** and **2** suppressed NO and PGE_2_ production, molecular docking approaches were used to identify possible binding targets to understand inhibitory actions at the atomic level. The binding affinity with respect to predicted fitness scores of **1** and **2** and targets [TLR-4 and p38-α MAPK proteins] were examined and compared to reference ligands (Table 1).

**Table 1:** Predicted binding affinity termed fitness scores toward focused receptor targets of **1**, **2**, and reference ligands.

Compounds	Fitness score*/receptor targets		
	P38-α (ATP-binding site)	P38-α (non ATP-binding site)	TLR4
BIRB-796	–	56.64	–
SB-203580	46.68	–	–
ZINC25778142	–	–	25.55
1	37.97	28.22	19.99
2	35.28	29.01	20.18

*Higher fitness scores indicated better binding affinity.

**Figure 5:**
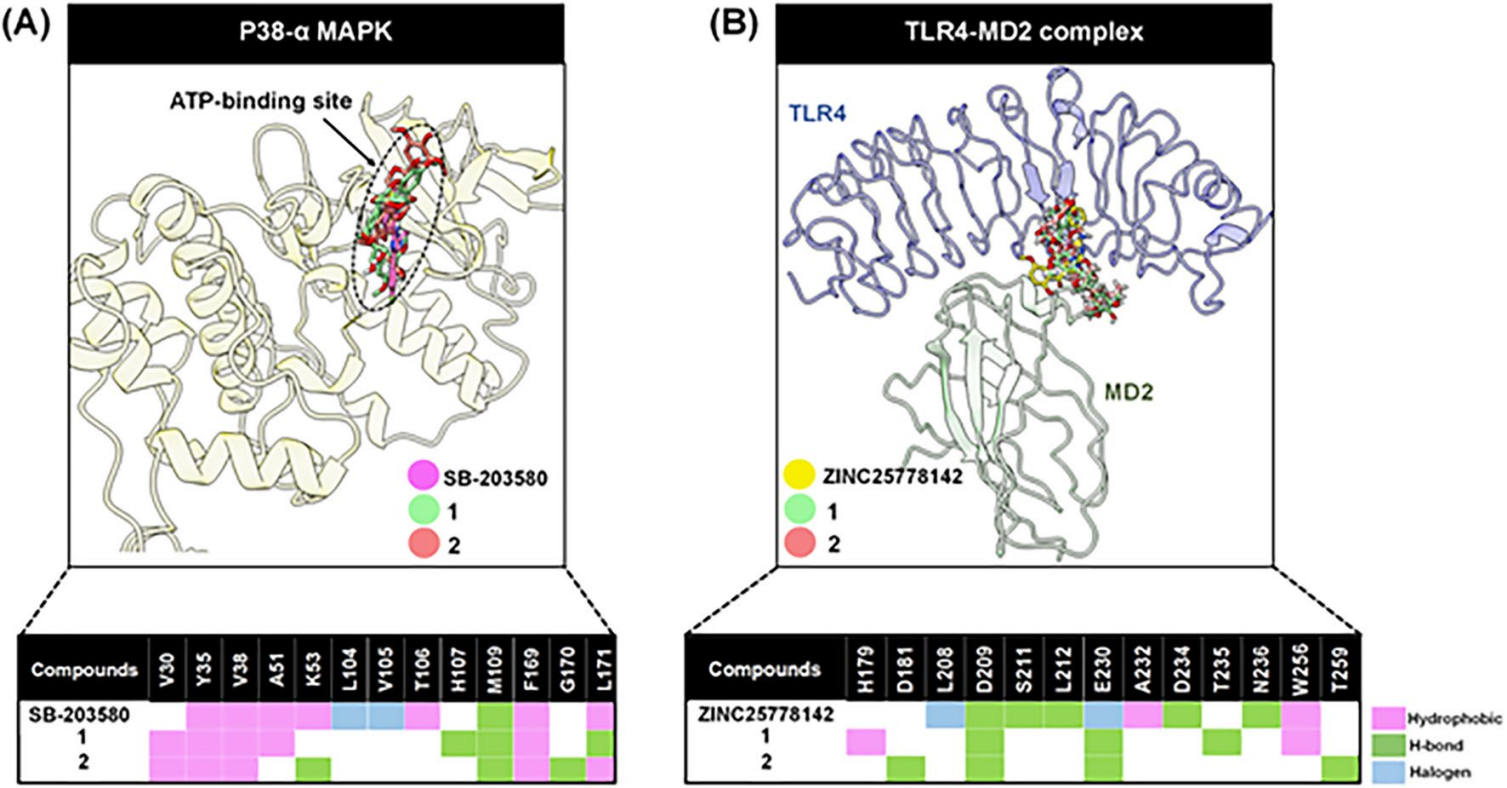
Focused ligand binding sites and ligand intermolecular interactions with surrounding amino acids of (**A**) p38-α MAPK at the ATP-binding site and (**B**) the TLR4-MD2 interface in complex with docked conformations of **1**, **2**, and reference compounds (SB203580 for P38-α MAPK at the ATP-binding site and ZINC25778142 for TLR-4); pink, green, and light blue denote hydrophobic, conventional hydrogen bond, and halogen interactions, respectively. This molecular recognition analysis was based on the rigid docked conformation predictions.

Molecular docking showed that **1** and **2** preferably bound to the ATP-binding pocket of p38-α rather than to the non-ATP (allosteric) site as their fitness scores for the allosteric pocket were much lower than BIRB-796, a p38-α non-ATP site inhibitor. For TLR-4, **1** and **2** exhibited approximately a 0.8-fold decrease in fitness score when compared with ZINC25778142, a TLR-4 inhibitor. Additionally, we investgatged protein–ligand intermolecular interactions in the ATP-binding site of p38-α MAPK and TLR4-MD2 (myeloid differentiation protien 2) interface using the most likely occurring conformation (highest fitness score) (Fig. 5A,B). These data showed **1** underwent hydrogen bonding interactions with H107, M109, and L171 and hydrophobic interactions with V30, Y35, V38, A51, and F169, whereas the amino acid residues K53, M109, and G170, and V30, Y35, V38, F169, and L171 interacted with **2** via hydrogen bonding and hydrophobic forces, respectively. For TLR-4, both buddlenols D **1** and **2** tended to interrupt the TLR4 interface via a transient construction of hydrogen bonds with D209, E230, and T235 for **1**, and D181, D209, E230, and T259 for **2**. Also, two hydrophobic interactions with H179 and W256 were observed in **1**.

Regarding to the selectivity of **1** and **2** toward other kinases in MAPK signaling pathway, the binding affinity ratios of **1** and **2** compared to corresponding crystallized ligand were evaluated. The results showed that binding affinity ratios of the **1** and **2** were 0.81 and 0.76 for p38α MAPK, respectively, while their binding affinity ratios against other kinases in MAPK pathway were ranged from 0.44 to 0.64 (Table 2). The computation study indicated that **1** and **2** selectively inhibited the p38α MAPK, which was corresponded well to the western blot results (Fig. 4).

**Table 2:** Binding affinity of **1** and **2** toward kinase targets involved in the MAPK signaling cascade compared to the original crystallized ligand.

Targets (PDB ID:)	Fitness score			Binding affinity ratio compared to corresponding crystallized ligand	
	Crystallized ligand	**1**	**2**	**1**	**2**
P38α MAPK (3ZSH)	46.68	37.97	35.28	0.81	0.76
ERK1 (4QTB)	54.28	30.57	29.11	0.56	0.54
ERK2 (1PME)	45.39	23.08	23.18	0.51	0.51
JNK1(2NO3)	36.54	22.97	23.28	0.63	0.64
JNK2 (3NPC)	67.35	30.57	31.41	0.45	0.47
JNK3 (4W4Y)	47.98	21.19	27.87	0.44	0.58
IκB kinase β (4KIK)	50.63	23.65	25.86	0.47	0.51

* The equation for binding affinity ratio was fitness score of the compound divided by fitness score of crystallized ligand.

The ADMET data (Table 3) showed that **1** and **2** were practically non-toxic and not irritant compounds, but they exhibited low bioavailability and violated the Lipinski’s rule-of-five (drug-likeness assessment).

**Table 3:** Calculated ADMET parameters of **1** and **2**.

Parameter *	compound	
	**1**	**2**
I. Physicochemical properties and pharmacokinetics		
MW (g/mol)	644.66	644.66
iLOGP	4.21	4.49
HBD	4	4
HBA	13	13
TPSA (Å^2^)	163.99	163.99
RB	13	13
nHA	46	46
MR	163.79	163.79
Log *S* (ESOL)	− 4.76 (moderately soluble)	− 4.76 (moderately soluble)
GI absorption	Low	Low
BBB permeant	No	No
P-gp substrate	No	No
CYP inhibitor (CYP1A2, 2C19, 2C9, 2D6, 3A4)	No	No
Lipinski’s rule-of-five	No; 2 violations: MW > 500, nitrogens or oxygens > 10	No; 2 violations: MW > 500, nitrogens or oxygens > 10
Bioavailability score	0.17	0.17
II. Toxicity		
LD_50_	1500 mg/kg	1500 mg/kg
Toxicity class	4	4
Mutagenicity	No	No
Tumorigenicity	Low	Low
Irritant	No	No
Reproductive effect	No	No

*The acronyms were referred to their full terms as follows: *MW* molecular weight, *iLOGP* octanol/water partition coefficient, *HBD* number of H-bond donors, *HBA* number of H-bond acceptors, *TPSA* topological polar surface area, *RB* rotatable bonds, *nHA* number of heavy atoms, *MR* molar refractivity, *Log S (ESOL)* decimal logarithm of the molar solubility in water (ESOL model), *GI absorption* gastrointestinal absorption, *BBB permeant* blood–brain barrier permeant, *P-gp substrate* permeability glycoprotein substrate, *CYP inhibitor* cytochrome P450 inhibitor, *LD*_*50*_ median lethal dose. Lipinski (rule-of-five) and Abbot bioavailability score are the assessment for the oral drug-candidates.

## Discussion

For the structural determination of buddlenols D **1** and **2**, adduct ions [M+Cl]^−^ at *m/z* of 679.2166 and 679.2156 were observed by HR-ESI–MS, and calculated for their molecular formula, C_33_H_40_O_13_ of **1** and **2**, respectively. From Heteronuclear Multiple Bond Correlation (HMBC) spectra, hydroxy protons at δ_H_ 7.05 and 7.15 of **1** were placed on C-4″ (δ_C_ 135.9, syringylglycerol subunit) and C-4′ (δ_C_ 136.3, a syringyl part of syringaresinol subunit), respectively. Additionally, a C8″-O-C4 ether linkage of both substructures was suggested by carbon chemical shifts at C-8″ (δ_C_ 88.0) and C-4 (δ_C_ 135.9)^18^. From NMR analysis, HMBC correlations [δ_H_ 7.06 (OH-4″)/C-4″ (δ_C_ 136.1), δ_H_ 7.15 (OH-4′)/C-4′ (δ_C_ 136.3)], and ^13^C-NMR signals of C-8″ (δ_C_ 89.4) and C-4 (δ_C_ 136.2) were also observed in **2**. Relative configurations on C-8″ and C-7″ of **1** and **2** were guided by NOESY correlations. The Nuclear Overhauser Effect Spectroscopy (NOESY) spectrum of **1** showed a prominent correlation of δ_H_ 4.18 (H-8″) and δ_H_ 4.98 (H-7″) and a relatively weak correlation of δ_H_ 4.18 (H-8″)/δ_H_ 4.38 (OH-7″), while a strong correlation of δ_H_ 4.00 (H-8″) and δ_H_ 4.34 (OH-7″) and a relatively pale correlation of δ_H_ 4.00 (H-8″)/δ_H_ 4.96 (H-7″) were observed in NOESY spectrum of **2**. Moreover, *J*_7′′,8′′_ of **1** and **2** were observed at 4 and 6.8 Hz, respectively, and corresponded with *erythro*- and *threo*- forms of an acyl glycerol moiety^18,19^. Thus, 7′′,8′′-*erythro* and 7′′,8′′-*threo* buddlenols D were deduced for **1** and **2**, respectively, and supported chemical shift differences for 7″ and 8′′ positions (Δ*δ*_*C8′′-C7′′*_) as suggested by Xiong et al.^19^; [Δ*δ*_*C8′′-C7′′*_: 14.3 and 15.3 for *erythro* and *threo* isomers, respectively]. From circular dichroism (CD) spectra of **1** and **2**, positive Cotton effects were observed at 239 and 233 nm, respectively, indicating the 8″*S* configuration of both compounds^19^. Additionally, negative benzene ^1^L_b_-band Cotton effects observed at 283 and 282 nm for **1** and **2**, respectively, were consistent with **4** [Δ*ε* − 3.75, 281.9 nm] and revealed 7*R*,7′*R*,8*S*,8′*S* configurations in these molecules^20^. The absolute configuration of benzylic carbon (C-7″) bearing a hydroxy group in **1** and **2** was suggested by Cotton effects in Rh_2_(OCOCF_3_)_4_-induced CD experiments, based on the secondary alcohol bulkiness rule. E band signs (≈ 350 nm) were observed as negative and positive Δ*ε* for **1** and **2**, respectively, suggesting the 7″*R* configuration for **1** and 7″*S* configuration for **2**^21^. Moreover, the assigned configurations of **1** (7′′*R*,8′′*S*-buddlenol D) and **2** (7′′*S*,8′′*S*-buddlenol D) were consistent with the electronic circular dichroism (ECD) spectrum calculated based on Density Functional Theory (DFT) (Supplementary Information: Figure S2–S42).

Buddlenol D was first isolated from *Buddleja davidii* (Scrophulariaceae)^22^. Later, the absolute configuration of two buddlenol D isomers (7′′*R*,8′′*S* and 7′′*S*,8′′*S*) were thoroughly investigated in work by Xiong et al.^19^. Both 7′′*R*,8′′*S*- and 7′′*S*,8′′*S*-buddlenols D were isolated from *Sinocalamus affinis* (Poaceae) and *Melodinus cochinchinesis* (Apocynaceae) using reverse phase chromatography^19,23^. In our study these 7″,8″-buddlenol D epimers were separated by silica gel column chromatography eluted in 20% Me_2_CO/CH_2_Cl_2_. Buddlenol D and syringaresinol are furofuran lignans comprised of synapyl alcohol units, while hedyotisol A comes from coniferyl alcohol monomers. In our study, phenolic compounds (**1**–**4, 8, 9,** and **11**) which possessed substructural guaiacyl and syringyl moieties, oligostilbenes (**5**, **6**), and a proanthocyanidin (**7**) demonstrated the influence of shikimate pathway on the biosynthesis of compounds stored in *C. brachiata* stem and bark. Although 7″,8″-buddlenol D epimers were isolated from the aforementioned plants, their biological activity and medicinal use were not previously addressed.

We also reported the anti-inflammatory activity of 7″,8″-buddlenol D epimers. The inhibition of PGE_2_, a pyrogenic mediator, via suppressed COX2 expression by **1** and **2** supported the antipyretic properties of *C. brachiata* stem and bark for traditional use, and was putatively related to the finding that a higher core temperature correlated with higher PGE_2_ production^24^. Additionally, **1** and **2** significantly inhibited iNOS expression induced by LPS treatment. As LPS-induced iNOS and COX2 expression, these results suggested **1** and **2** exerted anti-inflammatory activities by suppressing these proinflammatory enzymes and reducing NO and PGE_2_ levels. MAPK signal transduction, including ERK1/2, JNK, and p38 MAPK, modulates inflammatory processes in response to stimuli via different receptors^25^. ERK1/2, JNK, and p38 MAPK phosphorylation activities are the major downstream signaling phenomena after TLR4 activation. Therefore, molecules regulating TLR4-mediated ERK1/2, JNK, and p38 MAPK phosphorylation may exhibit anti-inflammatory potential. From our data, we hypothesized the underlying mechanisms of **1** and **2** may occurred via modulated p38 MAPK phosphorylation. In contrast, dexamethasone, which is an anti-inflammatory drug and used as a positive control inhibited all ERK1/2, JNK, and p38MAPK phosphorylation^26^ and exerted potent anti-inflammatory activity by suppressing NO and PGE_2_ production and down-regulating iNOS and COX2 expression^27^.

To examine if **1** and **2** inhibited p38 MAPK phosphorylation, a specific p38 inhibitor SB203580 was used to identify anti-inflammatory activity. In RAW264.7 cells pre-treated with SB203580 prior to LPS stimulation, p38 MAPK phosphorylation suppression was clearly observed and resulted in NO inhibition (Supplementary Information: Figure S6)^28,29^. The Western blot experiments showed that the compounds **1** and **2** inhibited p38 MAP kinase up to 24 h after LPS stimulation, suggesting that their inhibitory effects on proinflammatory signaling induced by LPS could last long. This last long effect was also observed in pre-treatment with a specific p38 inhibitor, SB203580. Although the activation of NF-κB or AP-1 was not investigated in this study, the decrease in expression of downstream inflammatory effectors, iNOS and COX2, could help explain that these suppressions were influenced by p38 kinase inhibition^30^ (Figs. 3, 4). Thus, aided by SB203580 inhibitory observations, we suggest that p38 phosphorylation inhibition by **1** and **2** may regulate anti-inflammatory processes^31^. Additionally, the anti-inflammatory and antioxidant (DPPH radical scavenging) activities of **1** and **2** support the use of *C. brachiata* extracts for oral stomatitis and wound healing^32^ (Supplementary Information: Table S1).

Next, we used in silico methods to investigate the anti-inflammatory mechanisms of **1** and **2** at the atomic level. Our binding affinity model data suggested **1** and **2**, as expected, bound TLR4 and p38-α MAPK; however, they both demonstrated a 0.8-fold lower binding capability when compared with reference ligands [ZINC25778142 and SB203580]. This may have been due to reduced ligand intermolecular interactions in the binding sites of targeted proteins. Based on our docking evidence, we hypothesize that **1** and **2** bound to both TLR-4 and p38-α MAPK at the ATP-binding site. However, immunoblot analyses suggested **1** and **2** suppressed a p38 MAPK downstream element while suppression of TLR4-mediated ERK1/2 and JNK was not observed. This implied that **1** and **2** may not bind effectively to TLR-4 since they did not inhibit the phosphorylation of all downstream elements^8^. Also, they did recognize smaller numbers of non-covalent interactions (Fig. 5B) when compared with the TLR-4 inhibitor reference (ZINC25778142). Thus, p38 MAPK may be a key player in compound binding, interrupting proinflammatory responses, and inhibiting the target as supported by computational and experimental studies. In addition to our binding analysis of p38-α MAPK, SB203580 exhibited higher intermolecular interactions when compared with **1** and **2 **(Fig. 5A), which included hydrogen bonding with M109 and hydrophobic interactions with V30, Y35, V38, A51, T106, F169, and L171. Also, two halogen interactions with L104 and V105 were identified. All residues interacting with **1** and** 2** were identified as contact residues in a previous report^33^, supporting the notion that **1** and** 2** docked at preferable sites and had the potential to inhibit p38-α MAPK. In addition to TLR4, ZINC25778142 showed more intermolecular interactions when compared with **1** and **2 **(Fig. 5B). These included five hydrogen bonds with D209, S211, L212, D234, and N236, hydrophobic interactions with A232, and W256, and two additional halogen bonds with L208 and E230. We also observed similar ZINC25778142 interactions with previously reported amino acid residues (D209, S211, and D234)^34,35^, thus verifying our docking protocol.

When combined, we suggest **1** and **2** bind to the ATP-binding pocket of p38-α MAPK and inhibit p38 phosphorylation, a downstream element of signaling pathway, rather than upstream TLR-4 inhibition. These results agree with the key roles for p38-α MAPK in regulating inflammatory responses, including iNOS and COX2 regulation, in macrophages^36^. However, future in-depth studies on **1** and **2** during LPS-TLR-4/MD-2 transduction are warranted.

Concerning the sharing features of ATP-binding pocket among kinases, we thus observed the binding capability toward other kinases involved in the focused signaling cascade. As listed in Table 2, the binding affinity based on the fitness score suggested that **1** and** 2** were most favorable to bind to p38-α MAPK at the ATP-binding site. The binding affinity ratio for p38-α MAPK at the ATP binding site compared to corresponding crystallized ligand was 0.81 and 0.76 for **1** and **2**, respectively, while the other kinases were falling into the smaller values (~ 0.4 to 0.6). Hence, molecular docking studies suggested that **1** and** 2** could selectively impede p38-α MAPK at the ATP-binding site.

Although, **1** and **2** might possess low GI absorption profile, based on in silico calculation **1** and **2** exhibited good metabolic profiles. Compounds **1** and **2** could be potential compounds for further structural development for better pharmacokinetic profiles and suitable drug delivery system. Moreover, after oral administration of **1** and **2**, they might lose the syringyl unit during the digestion process and could be transformed to enterodiol derivatives by gut microbiome^37^. There was evidence that enterodiol exhibited p38 down regulation as one of the mechanisms inhibiting the growth of colorectal cancer cells^38^.

In our previous work, we reported a natural p38 MAPK inhibitor (+)-*S*-deoxydihydroglyparvin which is a sulfur-containing propanamide derivative isolated from *Glycosmis parva* (Rutaceae) leaves^39^. As furofuran lignans such as epimagnolin B from *Magnolia fargesii* (Magnoliaceae), (−)-sesamin-2,2′-diol from *Isodon japonicus* (Lamiaceae), and zanthpodocarpins A and B from *Zanthoxylum podocarpum* (Rutaceae) displayed anti-inflammatory activity via NO inhibition in cultured cells^40–42^, we suggest this compound class may exhibit anti-inflammatory activity. From our study, we propose 7″,8″-buddlenol D epimers function as p38 MAPK inhibitors.

## Materials and methods

### Plant materials

Stems and barks from *C. brachiata* were collected in January 2018 from the botanical gardens of the Faculty of Pharmaceutical Sciences, Chulalongkorn University, Bangkok. The samples were identified by a study author (C. Chansriniyom). The herbarium specimen (CC-CB-010118) was deposited at the Department of Pharmacognosy and Pharmaceutical Botany, Chulalongkorn University. In addition, the handling and notification of stems and barks of *C. brachiata* were carried out in accordance with the Plant Variety Protection Act B.E. 2542 (1999): Section 53, the Kingdom of Thailand.

### Extraction and isolation

Dried and coarsely powdered *C. brachiata* stems and barks (4.0 kg) were successively macerated in MeOH (3 × 15 L) to yield a 203.80 g MeOH extract which was partitioned with hexane to generate a hexane extract (7.34 g). The remaining organic layer was added to water and further partitioned with EtOAc and *n*-butanol (*n*-BuOH) to obtain EtOAc (27.39 g), *n*-BuOH (50.39 g), and aqueous (113.89 g) extracts.

The EtOAc extract (27.39 g) underwent silica gel column chromatography (Flash column chromatography (Flash CC), PuriFlash^®^ XS 420, Advion Inc., NY, USA) and was eluted in a gradient solvent of MeOH, EtOAc and hexane at an initial ratio of 0:40:60 (respectively), then progressed to 30:70:0 (respectively), and terminated in 100% MeOH. Based on chromatographic patterns fractions were combined to generate seven fractions (CBE1–CBE7).

CBE7 (6.58 g) was separated into seven sub-fractions using Flash CC [Si (Silica gel 70–230 or 230–400 mesh, Merck, Darmstadt, Germany), a mobile phase of MeOH-CH_2_Cl_2_ system (8%, 12%, 20% MeOH/CH_2_Cl_2_, gradient manner)]. CBE7-3 (874 mg) was separated into 48 fractions using Medium Pressure Liquid Chromatography (MPLC, Eyela^®^ medium pressure pump VSP 3050, Kyoto, Japan) (Si, 4%MeOH/CH_2_Cl_2_). CBE7-3-12 (178.2 mg) was purified using MPLC (Si, 20%Me_2_CO/CH_2_Cl_2_) to obtain **1** (6 mg) and **2** (2.4 mg). CBE7-3-20 (88.9 mg) was separated by MPLC (Si, 20%Me_2_CO/CH_2_Cl_2_) to afford **3** (6 mg). CBE7-4 (466 mg) was purified using two MPLC steps (Si, 10%MeOH/ MeOH/CH_2_Cl_2_ and 6%MeOH/CH_2_Cl_2_) to achieve **8** (6.3 mg).

CBE6 (2.23 g) was separated by MPLC (Si, 80%EtOAc/hexane) into seven sub-fractions. CBE6-2 (453.8 mg) underwent MPLC (Si, 50%Me_2_CO/hexane) to generate 12 sub-fractions. Sub-fractions 5–6 (CBE6-2-(5–6), 110 mg) were combined and underwent two purification steps on an C18-reverse phase silica gel (RP-18, LiChroprep^®^, 25–40 μm, Merck, Darmstadt, Germany) column using 40%MeCN(acetonitrile)/water and Si, 4%MeOH/CH_2_Cl_2_ to obtain 18 mg of **4**. CBE6-2-10 (167 mg) was separated by MPLC (RP-18, 30%MeCN/water) to yield **7** (73.5 mg).

CBE5 (830 mg) was purified by MPLC (Si, EtOAc/CH_2_Cl_2_, gradient) to give 18 sub-fractions. CBE5-12 (36.6 mg) was separated by MPLC (Si, 2%MeOH/CH_2_Cl_2_) into 10 sub-fractions. Sub-fractions 8–10 (3.2 mg) were combined and subjected to Sephadex LH-20 column chromatography (50%MeOH/CH_2_Cl_2_) to provide **9** (2.0 mg). CBE5-13 (41.3 mg) was purified by MPLC (Si, 4%MeOH/CH_2_Cl_2_) to obtain **5** (4.1 mg). CBE5-14 (32.7 mg) was separated by MPLC (Si, 4%MeOH/CH_2_Cl_2_) to yield **10** (1.0 mg). CBE5-15 (44.0 mg) was purified by MPLC (RP-18, 30% MeCN/water) to generate CBE5-15-3 (10 mg) which was purified on a Sephadex™ LH-20 (GE Healthcare, Amersham, UK) column (MeOH) to yield **6** (6.8 mg).

CBE3-7 (222.9 mg), a lower polar fraction, was separated by MPLC (Si, EtOAc/CH_2_Cl_2_/hexane, 1:1:3) to yield **11** (3.6 mg).

### General procedures for structure determination

Ultraviolet (UV) spectra were recorded on a Jasco V-530 UV/VIS spectrophotometer (Jasco, Kyoto, Japan). Infrared spectra were recorded on a FTIR-4200 spectrophotometer (Attenuated total reflectance) (Jasco, Kyoto, Japan). Optical rotation data were recorded on a Jasco P-2000 polarimeter (Jasco, Kyoto, Japan). One- and two-dimensional nuclear magnetic resonance (1D- and 2D-NMR) spectra were obtained from Varian Unity AS400 (Varian, Inc. Vacuum Technologies, MA, USA), Bruker Ascend 400 NMR (Bruker, Karlsruhe, Germany), Bruker Advance NEO 400 MHz, or JEOL 500 NMR (JEOL USA, Inc. MA, USA) instruments. Electron spray ionization-mass spectrometry (ESI-MS) data were recorded on a VG-Biotech Quatro-5022 mass spectrometer (VG Biotech, Altrincham, UK). High resolution-electron spray ionization-mass spectrometry (HR-ESI–MS) data were recorded on an Agilent 6540 UHD Accurate-Mass Q-TOF mass spectrometer (Agilent Technologies, CA, USA). Circular dichroism (CD) experiments were performed using a Jasco J-815 circular dichroism spectrophotometer (Jasco, Kyoto, Japan). Rh_2_ (OCOCF_3_)_4_-induced CD studies were conducted in CH_2_Cl_2_ at a molar ratio of 1:0.4 compound to ligand^43^.

The spectroscopic data of **1** and **2** as followed: (−)-(7*R*,7′*R*,7′′*R*,8*S*,8′*S*,8′′*S*)-4′,4′′-dihydroxy-3,3′,3′′,5,5′,5′′-hexamethoxy-7,9′:7′,9-diepoxy-4,8′′-oxy-8,8′-sesquineolignan-7′′,9′′-diol (7″*R*,8″*S*-*erythro* buddlenol D, **1**); white solid. ^1^H-NMR (acetone-*d*_6_, 400 MHz) δ: 7.15 (1H, s, OH-4′), 7.05 (1H, s, OH-4″), 6.78 (2H, s, H-2, -6), 6.71 (2H, s, H-2″, -6″), 6.68 (2H, s, H-2′, -6′), 4.98 (1H, q, *J* = 4 Hz, H-7″), 4.74 (1H, d, *J* = 4.4 Hz, H-7), 4.68 (1H, d, *J* = 4.4 Hz, H-7′), 4.38 (1H, d, *J* = 4.4 Hz, OH-7″), 4.26 (2H, m, H-9a, 9′a), 4.18 (1H, m, H-8″), 3.88 (6H, s, OCH_3_-3, 5), 3.86 (2H, m, H-9b, -9′b), 3.84 (1H, m, H-9″a), 3.82 (6H, s, OCH_3_-3′, 5′), 3.80 (6H, s, OCH_3_-3″, 5″), 3.43 (1H, m, H-9″b), 3.12 (2H, m, H-8, -8′); ^13^C-NMR (acetone-*d*_6_, 100 MHz) δ: 154.3 (C-3, -5), 148.8 (C-3′, -5′), 148.5 (C-3″, -5″), 139.2 (C-1), 136.3 (C-4′), 135.9 (C-4, -4″), 133.2 (C-1′), 132.8 (C-1″), 104.9 (C-2″, -6″), 104.6 (C-2′, -6′), 104.2 (C-2, -6), 88.0 (C-8″), 86.8 (C-7′), 86.7 (C-7), 73.7 (C-7″), 72.6 (C-9, -9′), 61.1 (C-9″), 56.7 (OCH_3_-3, 5, 3′, 5′,3″, 5″), 55.4 (C-8, -8′); IR (ATR) cm^-1^: 3434 (broad), 2936, 2874, 1612, 1593, 1516, 1461, 1214, 1113; UV λ_max_ (MeOH) nm (log *ε*): 206 (4.58), 238 (3.82), 272 (2.99). ESI–MS *m*/*z*: 667 [M+Na]^+^. HR-ESI–MS m/z: 679.2166 [M+Cl]^−^, calcd. for C_33_H_40_O_13_Cl 679.2163. [α]^20^_D_: − 12.96° (*c* 1.25 × 10^–4^ g/mL, MeOH). CD nm (MeCN): 204.8 (Δ*ε* − 13.88), 208.7 (Δ*ε* − 1.50), 213.1 (Δ*ε* − 5.35), 217.9 (Δ*ε* + 3.23), 225.7 (Δ*ε* − 4.14), 234.3 (Δ*ε* + 2.06), 239.0 (Δ*ε* + 0.50), 244.6 (Δ*ε* − 0.73), 283.3 (Δ*ε* − 0.09). Rh_2_(OCOCF_3_)_4_-induced CD nm (CH_2_Cl_2_): 355.4 (Δ*ε* − 0.83) (Supplementary Information: Figure S7–S17).

(−)-(7*R*,7′*R*,7′′*S*,8*S*,8′*S*,8′′*S*)-4′,4′′-Dihydroxy-3,3′,3′′,5,5′,5′′-hexamethoxy-7,9′:7′,9-diepoxy-4,8′′-oxy-8,8′-sesquineolignan-7′′,9′′-diol (7″*S*,8″*S*-*threo* buddlenol D, **2**): white solid. ^1^H-NMR (acetone-*d*_6_, 400 MHz) δ: 7.15 (1H, s, OH-4′), 7.06 (1H, s, OH-4″), 6.76 (2H, s, H-2, -6), 6.74 (2H, s, H-2″, -6″), 6.68 (2H, s, H-2′, -6′), 4.96 (1H, dd, *J* = 6.8, 3.6 Hz, H-7″), 4.73 (1H, d, *J* = 4.0 Hz, H-7), 4.68 (1H, d, *J* = 4.0 Hz, H-7′), 4.34 (1H, d, *J* = 3.2 Hz, OH-7″), 4.25 (2H, m, H-9a, 9′a), 4.00 (1H, m, H-8″), 3.90 (6H, s, OCH_3_-3, 5), 3.87 (2H, m, H-9b, -9′b), 3.82 (6H, s, OCH_3_-3′, 5′), 3.79 (6H, s, OCH_3_-3″, 5″), 3.67 (1H, m, H-9″a), 3.36 (1H, m, H-9″b), 3.11 (2H, m, H-8, -8′); ^13^C-NMR (acetone-*d*_6_, 100 MHz) δ: 153.9 (C-3, -5), 148.8 (C-3′, -5′), 148.4 (C-3″, -5″), 139.3 (C-1), 136.3 (C-4′), 136.2 (C-4), 136.1 (C-4″), 133.2 (C-1′), 132.8 (C-1″), 105.4 (C-2″, -6″), 104.5 (C-2′, -6′), 104.0 (C-2, -6), 89.4 (C-8″), 86.8 (C-7′), 86.6 (C-7), 74.1 (C-7″), 72.6 (C-9, -9′), 61.6 (C-9″), 56.7 (OCH_3_-3, 5, 3′, 5′,3″, 5″), 55.4 (C-8, -8′); IR (ATR) cm^−1^: 3466 (broad), 2939, 2844, 1610, 1594, 1520, 1459, 1216, 1116; UV λ_max_ (MeOH) nm (log *ε*): 206 (4.64), 236 sh (3.93), 272 (3.24). ESI–MS *m*/*z*: 667 [M+Na]^+^. HR-ESI–MS m/z: 679.2156 [M+Cl]^−^, calcd. for C_33_H_40_O_13_Cl 679.2163. [α]^20^_D_: − 9.78° (*c* 4.6 × 10^–5^ g/mL, MeOH). CD nm (MeCN): 203.9 (Δ*ε* − 12.58), 207.7 (Δ*ε* + 3.04), 210.1 (Δ*ε* + 1.19), 214.8 (Δ*ε* + 6.30), 219.0 (Δ*ε* − 0.24), 221.4 (Δ*ε* + 0.42), 225.5 (Δ*ε* − 0.98), 233.1 (Δ*ε* + 0.61), 237.8 (Δ*ε* − 0.62), 282.2 (Δ*ε* − 0.04). Rh_2_(OCOCF_3_)_4_-induced CD nm (CH_2_Cl_2_): 347.2 (Δ*ε* + 0.81) (Supplementary Information: Figure S18–S28).

For compounds **3**–**11**, spectroscopic data and references were available (Supplementary Figure S29–S58).

### Cell culture and treatment

Mouse macrophage cells RAW264.7 (Catalog no. TIB-71, ATCC, Manassas VA, USA) were cultured in Dulbecco’s Modified Eagle Medium (DMEM) supplemented with 10% heat-inactivated fetal bovine serum in 5% CO_2_ at 37ºC. Cells were routinely passaged every 3 days. All compounds were dissolved in DMSO and diluted in DMEM (final DMSO concentration = 0.5%).

### NO production

Cells (5 × 10^5^ cells/mL) in 100 µL were seeded in a 96‑well plate and incubated for 24 h at 37 °C. After this, cells were treated with different compound concentrations and incubated for 24 h. Then, medium containing compound was replaced by phenol red-free medium containing LPS (Sigma-Aldrich, MO, USA) (final concentration = 500 µg/mL) and the cells were further incubated for 24 h. NO levels were determined by collecting 100 µL supernatants and mixing with 100 µL modified Griess reagent (Sigma-Aldrich). Reaction mixtures were incubated for 15 min at room temperature and absorbance measured at 560 nm using a microplate reader. NO levels were quantified by interpolation using a nitrite standard curve. Cells treated with dexamethasone (10 µM) were used as a positive control group.

### Prostaglandin E_2_ production

Cells (5 × 10^5^ cells/mL) in 100 µL were seeded in a 96‑well plate and incubated for 24 h at 37 °C. Then, cells were treated with different compound concentrations and incubated for 24 h. After that, medium containing compound was replaced by medium containing LPS (final concentration = 500 µg/mL) and the cells were incubated for another 24 h. Supernatants were collected and subjected to enzyme-linked immunosorbent assay for PGE_2_ (Enzo Life Sciences, NY, USA) following manufacturer’s instructions.

### Western blotting

Protein expression was determined in cell lysates from each treatment group at the same treatment manner with previous experiments. A 30-µg protein aliquot underwent sodium dodecyl sulfate–polyacrylamide gel electrophoresis and separated proteins were transferred to polyvinylidene fluoride membranes. The blots were blocked in 5% bovine serum albumin and probed with primary antibodies: anti-iNOS (ab15323), anti-COX2 (CST#2282), anti-phospho-ERK (CST#4376), anti-ERK (CST#4695), anti-phospho-JNK (CST#4668), anti-JNK (CST#9252), anti-phospho-p38 (CST#4511), anti-p38 (CST#9212), and followed by incubation with a horse radish peroxidase-conjugated secondary antibody. Protein bands were detected using a chemiluminescent substrate and images acquired using ImageQuant LAS 4000 (GE Healthcare, Buckinghamshire, UK). Relative protein band intensity was normalized to β-actin or GAPDH expression using ImageJ. SB203580 (Tokyo Chemical Industry, Japan) is a specific p38-α inhibitor and was used as a positive control for p38 phosphorylation inhibition.

### Computational studies

#### Molecular docking

Protein–ligand docking was conducted for eight targeted proteins under nine systems-TLR4, p38-α MAPK (ATP-binding site), p38-α MAPK (non-ATP-binding site), ERK1, ERK2, JNK1, JNK2, JNK3 and IκB kinase β using crystallized structures (retrieved from RCSB Protein Data Bank), listed as follows: 2Z65^44^, 3ZSH^33^, 1KV2^45^, 4QTB^46^, 1PME^47^, 2NO3^48^, 3NPC^49^, 4W4Y^50^, 4KIK^51^, respectively. In addition to protein preparation, the 3-dimensional structures of **1** and **2** were manually constructed using the Gaussian 09 program and optimized using the HF/6-31d basis set implemented in the program^52^. For docking studies, the Gold docking program was used, and protocols were as follows: (1) the ligand binding site was defined as 6 Å for sphere docking and (2) ChemScore was used for the scoring function. Binding between proteins and compounds was visualized in Accelrys Discovery Studio 2.5 (Accelrys Inc.).

For all systems, docking was validated using aforementioned protocols to redock the corresponding crystallized ligand (Table S2), followed by aligning redocked pose with its original conformation to compare its similarity termed root-mean-squared-deviation (RMSD) of structure coordinates. For TLR4, since no crystallized small molecule inhibitors targeting the TLR-MD2 protein–protein interface were available, docking was performed by rational validation. Briefly, we manually docked a previously studied compound (ZINC25778142)^34^ and the docked pose was then observed its orientation and intermolecular interactions with the key reported residues including D209, S211, and D234. Our docking protocols were validated for all systems (Supplementary Information: Figure S61).

#### ADMET prediction

Pharmacokinetic properties (Absorption, Distribution, Metabolism and Excretion) of compounds **1** and **2** were calculated using SwissADME platform (http://www.swissadme.ch/)^53^ while their toxicity profiles were predicted using Protox-II platform (https://tox-new.charite.de/protox_II/)^54^ and the DataWarrior software package^55^.

### Statistical analysis

All data were expressed as the mean ± standard error of the mean from at least three independent experiments. Differences among groups were evaluated using one-way analysis of variance, followed by post-hoc tests with Bonferroni correction. Statistical significance was accepted at *p* < 0.05.

## Conclusions

For the first time, we characterized the anti-inflammatory activities of 7″,8″-buddlenol D epimers from *C. brachiata* stem and bark. Their action mechanisms were mediated by inhibited p38 MAPK phosphorylation which attenuated iNOS and COX2 expression and inhibited NO and PGE_2_ production in LPS-induced RAW264.7 macrophages. These findings support the use of *C. brachiata* for antipyretic, oral stomatitis, and wound healing purposes. Therefore, 7″,8″-buddlenol D epimers can be used as biological markers for *C. brachiata* extract.

## Supplementary Information

Supplementary Information.

## Data Availability

Spectroscopic and experimental data are available. However, the compounds have almost run out. Please contact corresponding author for information.
