# Supplementary material for: In vitro and in silico studies of 7′′,8′′-buddlenol D anti-inflammatory lignans from Carallia brachiata as p38 MAP kinase inhibitors
Source: Sci Rep. 2023 Mar 2;13:3558. doi: 10.1038/s41598-023-30475-5 (PMC9981598; doi:10.1038/s41598-023-30475-5)
Supplement: Supplementary file 1 — Supplementary Information. [file 41598_2023_30475_MOESM1_ESM.pdf]

Supplementary Information

In vitro and *in silico* studies of 7′′,8′′-buddlenol D anti-inflammatory lignans from *Carallia brachiata* as p38 MAP kinase inhibitors

Nonthaneth Nalinratana[a], Utid Suriya[b], Chanyanuch Laprasert[c], Nakuntwalai Wisidsri[d], Preeyaporn Poldorn[e], Thanyada Rungrotmongkol[e, f], Wacharee Limpanasitthikul[c], Ho-Cheng Wu[g], Hsun-Shuo Chang[h], and Chaisak Chansriniyom[i, j, *]

[a]Department of Pharmacology and Physiology, Faculty of Pharmaceutical Sciences, Chulalongkorn University, Bangkok, 10330, Thailand
[b]Program in Biotechnology, Faculty of Science, Chulalongkorn University, Bangkok 10330, Thailand
[c]Department of Pharmacology, Faculty of Medicine, Chulalongkorn University, Bangkok, 10330, Thailand
[d]Faculty of Integrative Medicine,
Rajamangala University of Technology Thanyaburi, Pathum Thani 12130,
Thailand
[e]Biocatalyst and Environmental Biotechnology Research Unit, Department of Biochemistry, Faculty of Science, Chulalongkorn University, Bangkok 10330, Thailand
[f]Program in Bioinformatics and Computational Biology, Graduate School, Chulalongkorn University, Bangkok 10330, Thailand
[g]Graduate Institute of Pharmacognosy, College of Pharmacy, Taipei Medical University, Taipei 110, Taiwan
[h]School of Pharmacy, College of Pharmacy, Kaohsiung Medical University, Kaohsiung 807, Taiwan
[i]Department of Pharmacognosy and Pharmaceutical Botany, Faculty of Pharmaceutical Sciences, Chulalongkorn University, Bangkok, 10330, Thailand
[j]Natural products and Nanoparticles Research Unit, Chulalongkorn University, Bangkok, 10330, Thailand

* Corresponding author: Chaisak Chansriniyom, Tel: +66-2218-8347, E-mail address: chaisak.ch@chula.ac.th

Table of Contents

		Page
Table S1	DPPH radical scavenging and NO production inhibitory activities of the isolates	5
Figure S1	HPTLC chromatograms of (−)-(7″*R*,8″*S*)-buddlenol D (**1**) and (−)-(7″*S*,8″*S*)-buddlenol D (**2**) thrice developed with 20% acetone in dichloromethane under (**A**) UV light 254 nm, and (**B**) visualizing with cerium (III) sulfate reagent	6
Figure S2	ECD spectrum of **1** calculated based on DFT method	7
Figure S3	ECD spectrum of **2** calculated based on DFT method	8
Figure S4	Illustration of Fischer projection of (−)-(7″*R*,8″*S*)-buddlenol D (**1**) and (−)-(7″*S*,8″*S*)- buddlenol D (**2**)	9
Figure S5	Effect of **1** and **2** on RAW264.7 cell viability by Resazurin assay. (**A**) RAW264.7 cells were treated with **1** and **2** for 24 h. (**B**) RAW264.7 cells, which were treated with **1** and **2** for 24 h and their medium was replaced by LPS-containing medium (final concentration 500 ng/mL), were further incubated for 24 h. Data were represented as mean ± standard error of the mean of three independent experiments (n=3).	10
Figure S6	Effect of SB203580 on (**A**) inhibition of LPS-induced NO production in RAW264.7 macrophages with (**B**) calculated IC_{50} values compared to compounds **1** and **2** (**C**) Representative immunoblots and densitometric analysis of the effect of SB203580 on p38 phosphorylation in LPS-induced RAW264.7 macrophages	11
Figure S7	High resolution ESI-MS of (−)-(7″*R*,8″*S*)-buddlenol D (**1**), $[M+Cl]^-$ at *m/z* of 679.2166	12
Figure S8	ESI-MS of (−)-(7″*R*,8″*S*)-buddlenol D (**1**), $[M+Na]^+$ *m/z* 667	12
Figure S9	^{1}H-NMR spectrum of (−)-(7″*R*,8″*S*)-buddlenol D (**1**), (400 MHz, acetone-d_6)	13
Figure S10	^{13}C-NMR spectrum of (−)-(7″*R*,8″*S*)-buddlenol D (**1**), (100 MHz, acetone-d_6)	14
Figure S11	HSQC spectrum of (−)-(7″*R*,8″*S*)-buddlenol D (**1**), (100 MHz, acetone-d_6)	14
Figure S12	HMBC spectrum of (−)-(7″*R*,8″*S*)-buddlenol D (**1**), (100 MHz, acetone-d_6)	15
Figure S13	HMBC spectrum of (−)-(7″*R*,8″*S*)-buddlenol D (**1**), (100 MHz, acetone-d_6), (continued)	15
Figure S14	^{1}H-^{1}H COSY spectrum of (−)-(7″*R*,8″*S*)-buddlenol D (**1**), (100 MHz, acetone-d_6)	16
Figure S15	NOESY spectrum of (−)-(7″*R*,8″*S*)-buddlenol D (**1**), (100 MHz, acetone-d_6)	16
Figure S16	Selected HMBC and NOESY correlations of (−)-(7″*R*,8″*S*)-buddlenol D (**1**)	17
Figure S17	CD spectra of (−)-(7″*R*,8″*S*)-buddlenol D (**1**), (MeCN): (**A**) Y-axis : milli degree, (**B**) Y-axis : $\Delta\varepsilon$, and (**C**) $Rh_2(OCOCF_3)_4$-induced CD spectrum, (CH_2Cl_2): Y-axis : $\Delta\varepsilon$	17

		Page
Figure S18	High resolution ESI-MS of (−)-(7″*S*,8″*S*)-buddlenol D (**2**), $[M+Cl]^-$ at *m/z* of 679.2156	18
Figure S19	ESI-MS of (−)-(7″*S*,8″*S*)- buddlenol D (**2**), $[M+Na]^+$ *m/z* 667	18
Figure S20	^{1}H-NMR spectrum of (−)-(7″*S*,8″*S*)-buddlenol D (**2**), (400 MHz, acetone-d_6)	19
Figure S21	^{13}C-NMR spectrum of (−)-(7″*S*,8″*S*)-buddlenol D (**2**), (100 MHz, acetone-d_6)	20
Figure S22	HSQC spectrum of (−)-(7″*S*,8″*S*)-buddlenol D (**2**), (100 MHz, acetone-d_6)	20
Figure S23	HMBC spectrum of (−)-(7″*S*,8″*S*)-buddlenol D (**2**), (100 MHz, acetone-d_6)	21
Figure S24	HMBC spectrum of (−)-(7″*S*,8″*S*)-buddlenol D (**2**), (100 MHz, acetone-d_6), (continued)	21
Figure S25	^{1}H-^{1}H COSY spectrum of (−)-(7″*S*,8″*S*)-buddlenol D (**2**), (100 MHz, acetone-d_6)	22
Figure S26	NOESY spectrum of (−)-(7″*S*,8″*S*)-buddlenol D (**2**), (100 MHz, acetone-d_6)	22
Figure S27	Selected HMBC and NOESY correlations of (−)-(7″*S*,8″*S*)-buddlenol D (**2**)	23
Figure S28	CD spectra of (−)-(7″*S*,8″*S*)- buddlenol D (**2**), (MeCN): (**A**) Y-axis : milli degree, (**B**) Y-axis : $\Delta\varepsilon$, and (**C**) $Rh_2(OCOCF_3)_4$-induced CD spectrum, (CH_2Cl_2): Y-axis : $\Delta\varepsilon$	23
Figure S29	ESI-MS of (+)-7″*R*,8″*S*:7‴*R*,8‴*S*-hedyotisol A (**3**), $[M+Na]^+$ *m/z* 833	24
Figure S30	^{1}H-NMR spectrum of (+)-7″*R*,8″*S*:7‴*R*,8‴*S*-hedyotisol A (**3**), (400 MHz, acetone-d_6)	24
Figure S31	^{13}C-NMR spectrum of (+)-7″*R*,8″*S*:7‴*R*,8‴*S*-hedyotisol A (**3**), (100 MHz, acetone-d_6)	25
Figure S32	CD spectra of (+)-7″*R*,8″*S*:7‴*R*,8‴*S*-hedyotisol A (**3**), (MeCN): (**A**) Y-axis : milli degree, (**B**) Y-axis : $\Delta\varepsilon$, and (**C**) $Rh_2(OCOCF_3)_4$-induced CD spectrum, (CH_2Cl_2): Y-axis : $\Delta\varepsilon$.	26
Figure S33	ESI-MS of (–)-syringaresinol (**4**), $[M+H]^+$ *m/z* 419	27
Figure S34	^{1}H-NMR spectrum of (–)-syringaresinol (**4**), (400 MHz, $CDCl_3$)	27
Figure S35	^{13}C-NMR spectrum of (–)-syringaresinol (**4**), (100 MHz, $CDCl_3$)	28
Figure S36	CD spectra of (–)-syringaresinol (**4**), (MeCN): (**A**) Y-axis : milli degree, (**B**) Y-axis : $\Delta\varepsilon$.	29
Figure S37	ESI-MS of (+)-diptoindonesin D (**5**), $[M+H]^+$ *m/z* 379	30
Figure S38	^{1}H-NMR spectrum of (+)-diptoindonesin D (**5**), (500 MHz, acetone-d_6)	30
Figure S39	^{13}C-NMR spectrum of (+)-diptoindonesin D (**5**), (125 MHz, acetone-d_6)	31
Figure S40	CD spectrum of (+)-diptoindonesin D (**5**), (MeOH), [Y-axis : milli degree]	31
Figure S41	ESI-MS of (+)-parviflorol (**6**), $[M+H]^+$ *m/z* 381	32
Figure S42	^{1}H-NMR spectrum of (+)-parviflorol (**6**), (500 MHz, acetone-d_6)	32
Figure S43	^{13}C-NMR spectrum of (+)-parviflorol (**6**), (125 MHz, acetone-d_6)	33
Figure S44	CD spectrum of (+)-parviflorol (**6**), (MeOH), [Y-axis : milli degree]	34

		Page
Figure S45	ESI-MS of (–)-mahuanin A (**7**), $[M+H]^+$ *m*/*z* 545	35
Figure S46	^{1}H-NMR spectrum of (–)-mahuanin A (**7**), (400 MHz, acetone-d_6)	35
Figure S47	^{13}C-NMR spectrum of (–)-mahuanin A (**7**), (100 MHz, acetone-d_6)	36
Figure S48	CD spectrum of (–)-mahuanin A (**7**), (MeOH), [Y-axis : milli degree]	37
Figure S49	ESI-MS of 4-hydroxy-2-methoxyphenyl-6-*O*-syringoyl-β-D-glucopyranoside (**8**), $[M+H]^+$ *m*/*z* 483	38
Figure S50	^{1}H-NMR spectrum of 4-hydroxy-2-methoxyphenyl-6-*O*-syringoyl-β-D-glucopyranoside (**8**), (400 MHz, acetone-d_6/D_2O (5:1 v/v))	38
Figure S51	^{13}C-NMR spectrum of 4-hydroxy-2-methoxyphenyl-6-*O*-syringoyl-β-D-glucopyranoside (**8**), (100 MHz, acetone-d_6/D_2O (5:1 v/v))	39
Figure S52	ESI-MS of vanillin (**9**), $[M+H]^+$ *m*/*z* 169	40
Figure S53	^{1}H-NMR spectrum of vanillin (**9**), (400 MHz, $CDCl_3$)	40
Figure S54	ESI-MS of protocatechuic acid (**10**), $[M+H]^+$ *m*/*z* 155	41
Figure S55	^{1}H-NMR spectrum of protocatechuic acid (**10**), (400 MHz, CD_3OD)	41
Figure S56	ESIMS of syringaldehyde (**11**), $[M+H]^+$ *m*/*z* 183	42
Figure S57	^{1}H-NMR spectrum of syringaldehyde (**11**), (400 MHz, $CDCl_3$)	42
Figure S58	^{13}C-NMR spectrum of syringaldehyde (**11**), (100 MHz, $CDCl_3$)	43
Figure S59	The original Western blots of presenting in **Figure 3**	44
Figure S60	The original Western blots of presenting in **Figure 4**	45-46
Figure S61	Validation of docking protocols used in this study. (**A-H**) Alignment of the redocked pose and available crystallized ligand for focused kinases in the molecular docking study as well as calculated RMSD value derived from superimposition of two structures (the lower RMSD indicates greater overlapping). (**I**) The orientation within the TLR4-MD2 interface of the docked conformation (ZINC25778142 compound) used as a reference and its intermolecular interactions with the key reported residues including D209, S211, and D234.	47
Table S2	Targeted proteins and crystallized ligand used in *in silico* studies	48

Table S1 DPPH radical scavenging and NO production inhibitory activities of the isolates

Compounds	DPPH radical scavenging assay[a] IC_{50}(μM)	Inhibition of NO production[b] IC_{50}(μM)
**1**<br>(−)-(7″*R*,8″*S*)-buddlenol D	52.14 ± 1.99	9.25 ± 2.69
**2**<br>(−)-(7″*S*,8″*S*)-buddlenol D	53.24 ± 0.43	8.43 ± 1.20
**3**<br>(+)-7″*R*,8″*S*:7‴*R*,8‴*S*-hedyotisol A	80.37 ± 1.46	>30
**4**<br>(−)-syringaresinol	46.85 ± 1.27	>30
**5**<br>(+)-diptoindonesin D	>100	>30
**6**<br>(+)-parviflorol	>100	>30
**7**<br>(−)-mahuanin A	64.82 ± 1.80	>30
**8**<br>4-hydroxy-2-methoxyphenyl-6-*O*-syringoyl-β-D-glucopyranoside	>100	>30
**9**<br>vanillic acid	>100	>30
**10**<br>protocatechuic acid	62.29±1.37	>30
**11**<br>syringaldehyde	>100	>30
ascorbic acid	30.23±1.61	
dexamethasone		78.69 ± 1.48% (10 μM)

[a] The maximum concentration used in DPPH scavenging assay was 100 μM. Briefly, 25 μL of the compounds was mixed with 50 μL of 0.2 mM DPPH (Sigma-Aldrich, USA) solution (in methanol) to give final concentrations of 1, 10, and 100 μM. The mixture solution was incubated for 30 min without direct exposure to light. The absorbance was determined at 520 nm using a microplate reader. Ascorbic acid and 2% DMSO were used as the positive and solvent controls, respectively.

%DPPH radical scavenging activity = $[(OD_{control} - OD_{sample}) / OD_{control}) \times 100$,

where OD is the optical density

[b] The NO levels in supernatant of treated cells were measured by Griess assay. The maximum concentration used in the assay was 30 μM.

Data were expressed as mean ± standard error of the mean (n = 3).

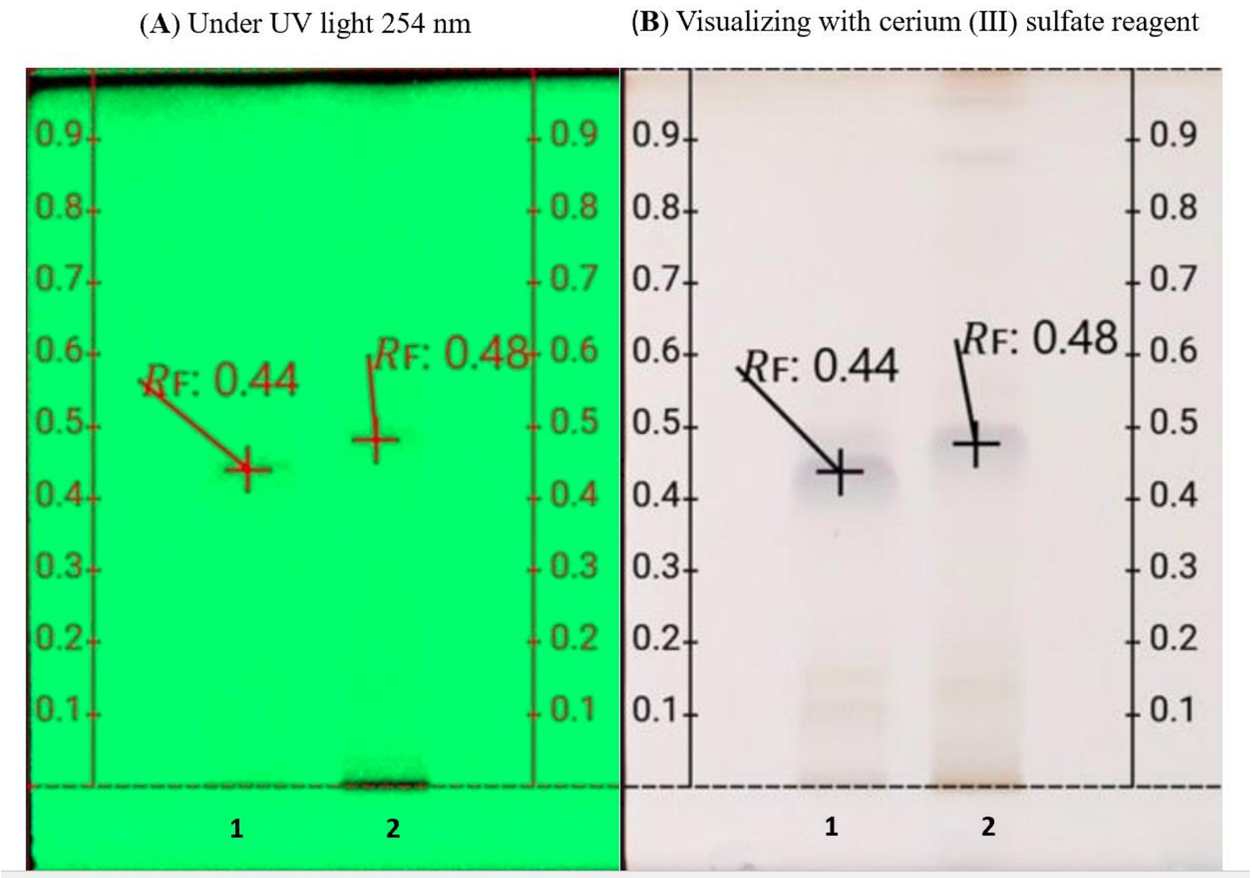

Figure S1. HPTLC chromatograms of (−)-(7″*R*,8″*S*)-buddlenol D (**1**) and (−)-(7″*S*,8″*S*)-buddlenol D (**2**) triply developed with 20% acetone in dichloromethane under (**A**) UV light 254 nm, and (**B**) visualizing with cerium (III) sulfate reagent

HPTLC instruments (CAMAG, Muttenz, Switzerland) were used for this planar chromatography experiment.

DFT calculations

Computational method

All configurations of **1** and **2** in this work were optimized using DFT approach at the B3LYP/6-31G(d,p) level of theory. The excited states were calculated by using TD-DFT at the B3LYP functional with TZVP basis. The geometry optimization and TD-DFT calculations were both performed with Continuum Model (PCM) solvation model with acetonitrile. The rotary strengths of 90 excited states were calculated. All calculations were performed using Gaussian16 program package with σ=0.3 eV fitting parameter using SpecDis1.64 program.

Reference:

Frisch, M. *et al.* Gaussian 16, Revision A. 03, Gaussian, Inc., Wallingford CT. *See also: URL: http://www. gaussian. com* (2016).

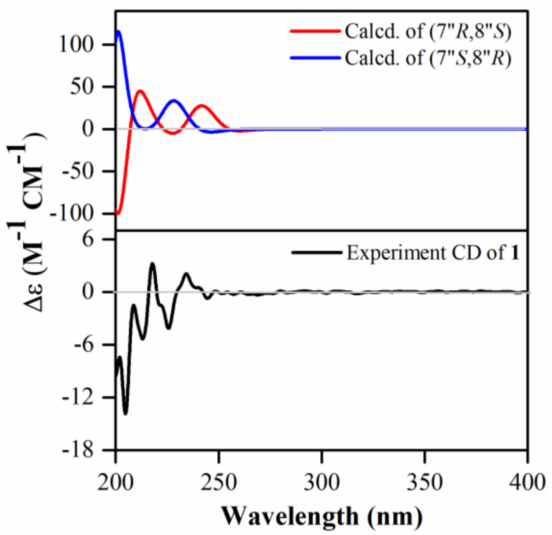

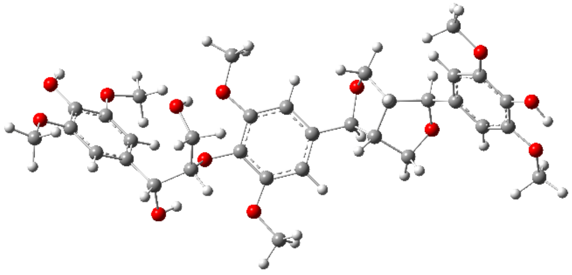

7″*R*,8″*S* (-2259.645201 Ha)

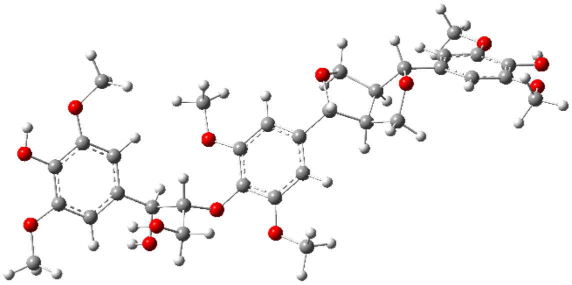

7″*S*,8″*R* (-2259.639377 Ha)

Figure S2. ECD spectrum of **1** calculated based on DFT method

The result deduced compound **1** to be 7″*R*,8″*S*-buddlenol D.

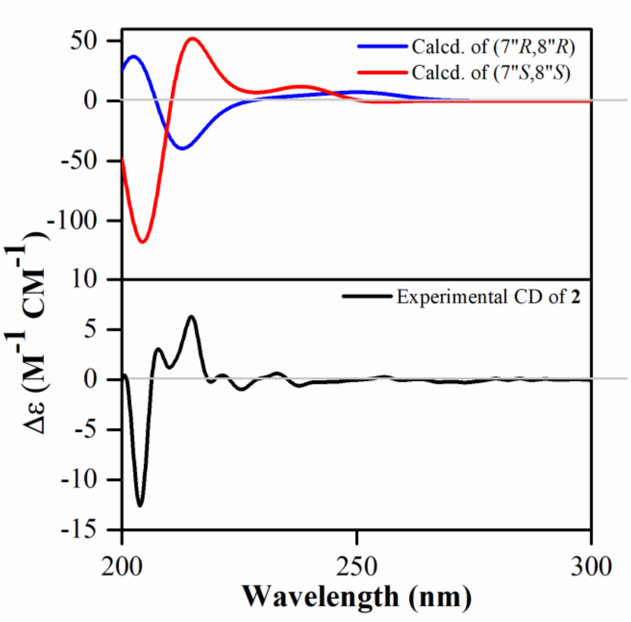

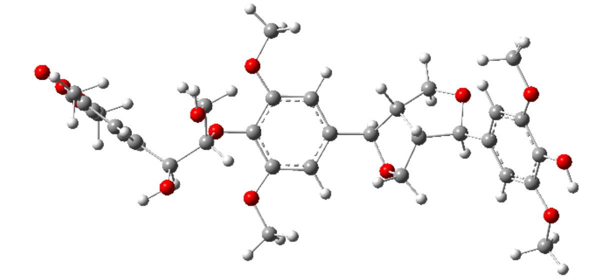	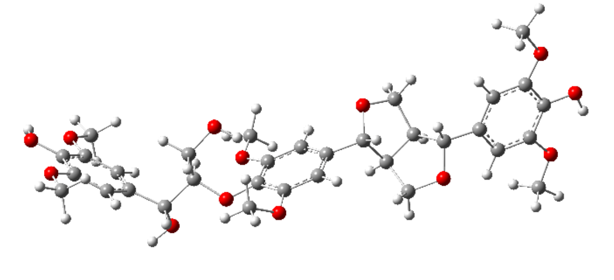
7″*S*,8″*S* (-2259.634290Ha)	7″*R*,8″*R* (-2259.628704 Ha)

Figure S3. ECD spectrum of **2** calculated based on DFT method

The result deduced compound **2** to be 7″*S*,8″*S*-buddlenol D.

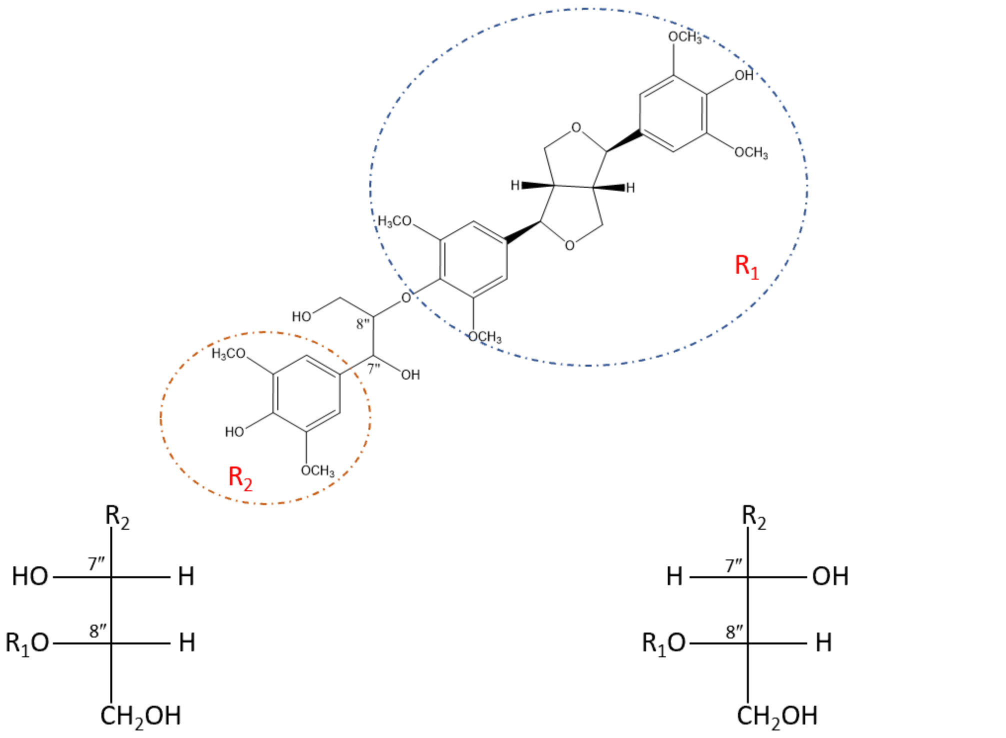

(−)-(7″R,8″S)-buddlenol D (**1**)
(−)-7″8″-erythro buddlenol D (**1**)

(−)-(7″S,8″S)- buddlenol D (**2**)
(−)-7″,8″-threo buddlenol D (**2**)

Figure S4: Illustration of Fischer projection of (−)-(7″*R*,8″*S*)-buddlenol D (**1**) and (−)-(7″*S*,8″*S*)-buddlenol D (**2**)

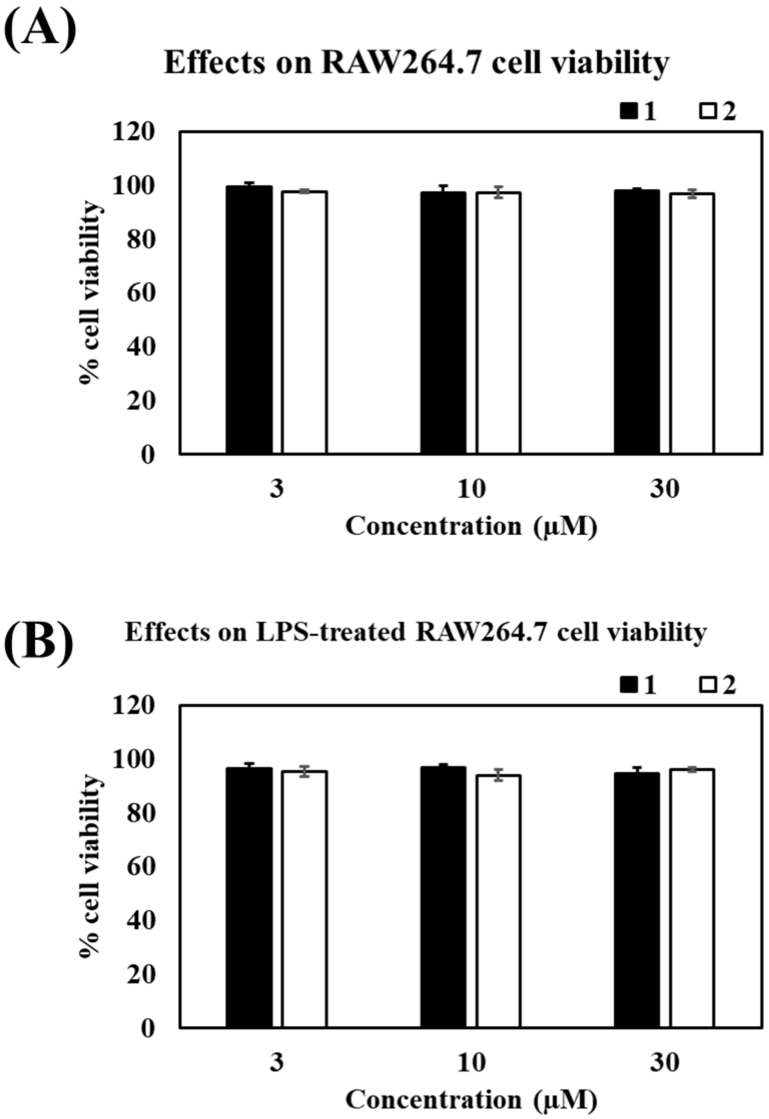

Figure S5. Effect of **1** and **2** on RAW264.7 cell viability by Resazurin assay. (**A**) RAW264.7 cells were treated with **1** and **2** for 24 h. (**B**) RAW264.7 cells, which were treated with **1** and **2** for 24 h and their medium was replaced by LPS-containing medium (final concentration 500 ng/mL), were further incubated for 24 h. Data were represented as mean ± standard error of the mean of three independent experiments (n=3).

(A)

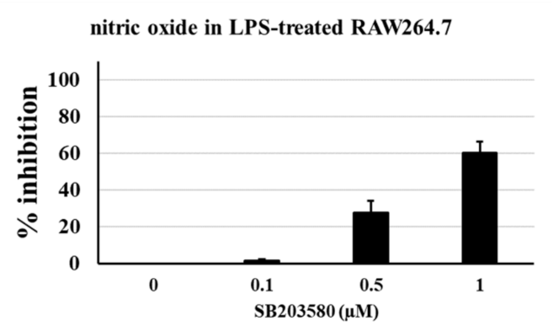

(B)

IC_{50} of % inhibition on LPS-induced nitric oxide production

	1	2	SB203580
NO IC_{50} (μM)	9.25 ± 2.69	8.43 ± 1.20	0.82 ± 0.05

(C)

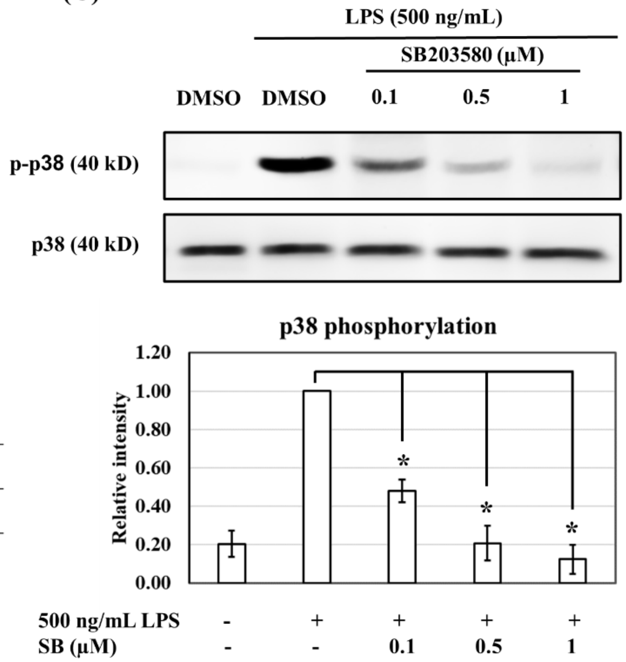

Figure S6. Effect of SB203580 on (**A**) inhibition of LPS-induced NO production in RAW264.7 macrophages with (**B**) calculated IC_{50} values compared to compound **1** and **2**. (**C**) Representative immunoblots and densitometric analysis of the effect of SB203580 on p38 phosphorylation in LPS-induced RAW264.7 macrophages.

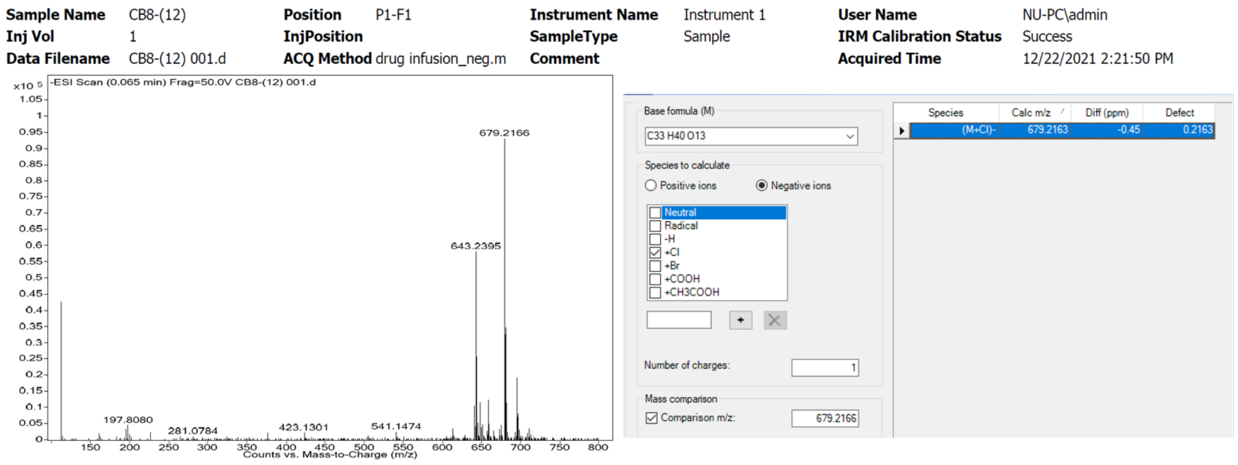

Figure S7: High resolution ESI-MS of (−)-(7″*R*,8″*S*)-buddlenol D (**1**), [M+Cl]$^-$ at *m/z* of 679.2166

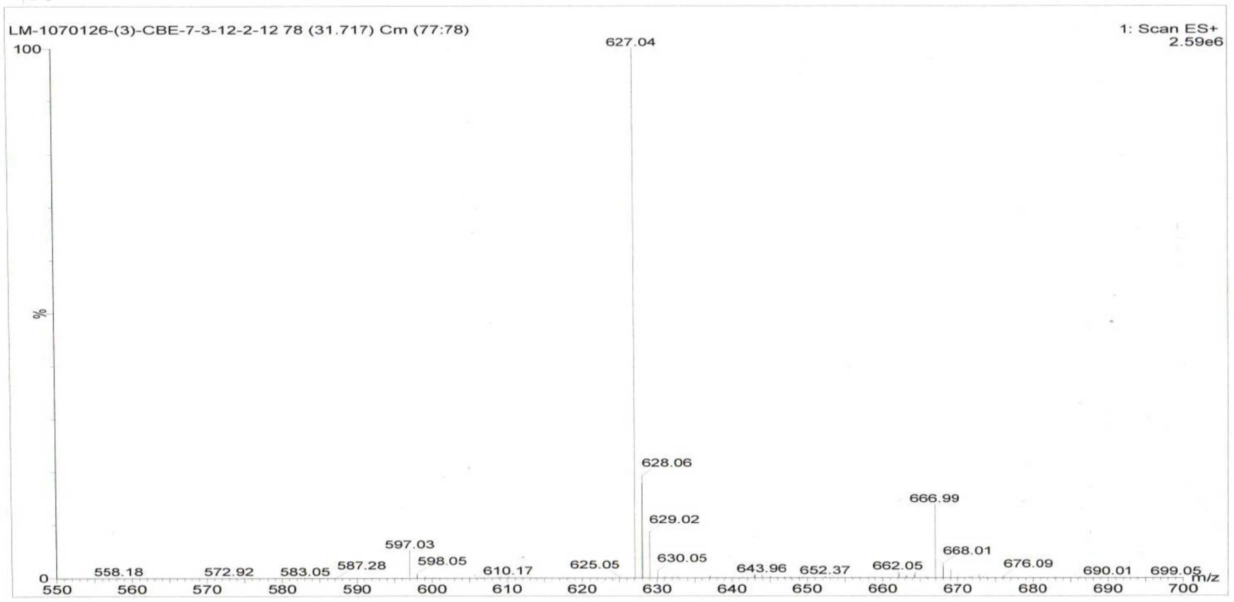

Figure S8: ESI-MS of (−)-(7″*R*,8″*S*)-buddlenol D (**1**), [M+Na]$^+$ *m/z* 667

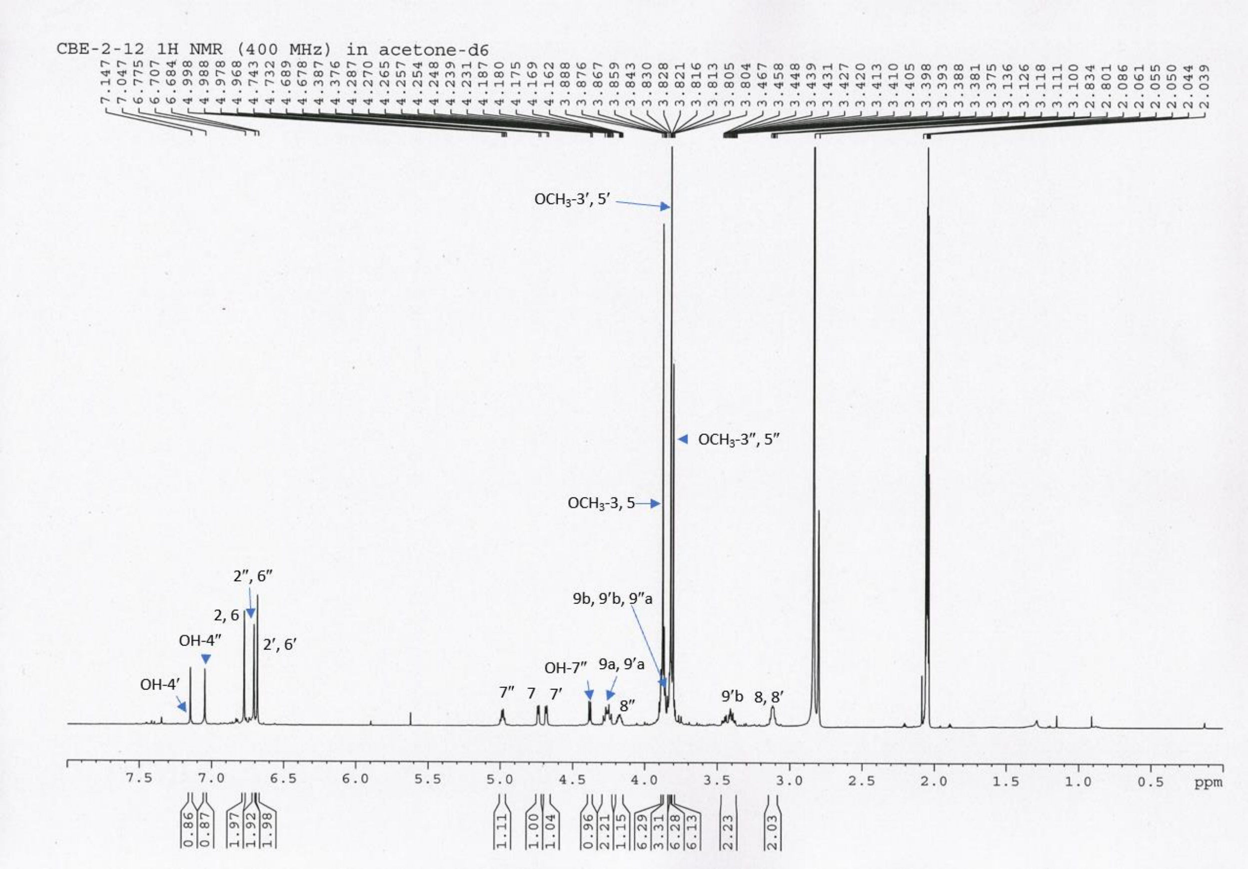

Figure S9: ^{1}H-NMR spectrum of (−)-(7″*R*,8″*S*)-buddlenol D (**1**), (400 MHz, acetone-d_6)

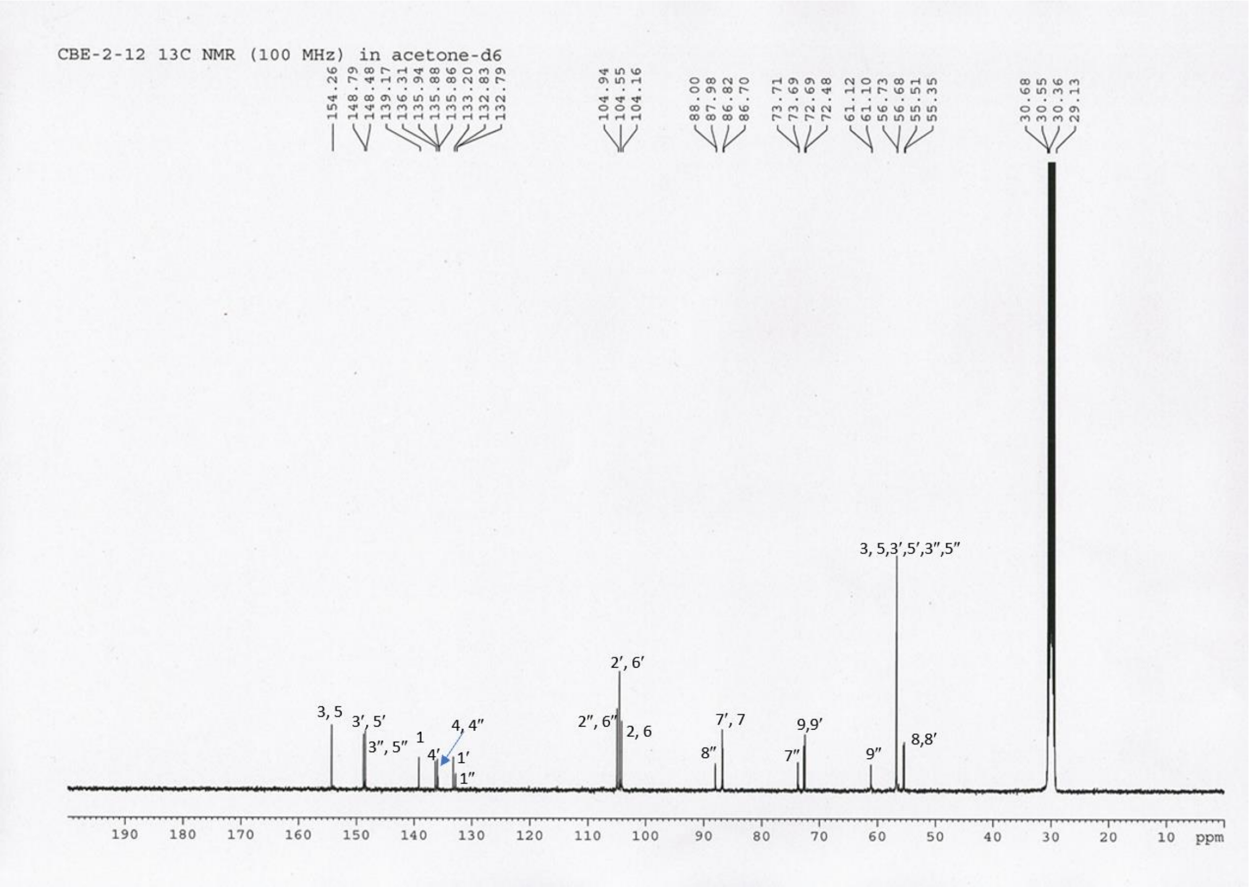

Figure S10: ^{13}C-NMR spectrum of (−)-(7″*R*,8″*S*)-buddlenol D (**1**), (100 MHz, acetone-d_6)

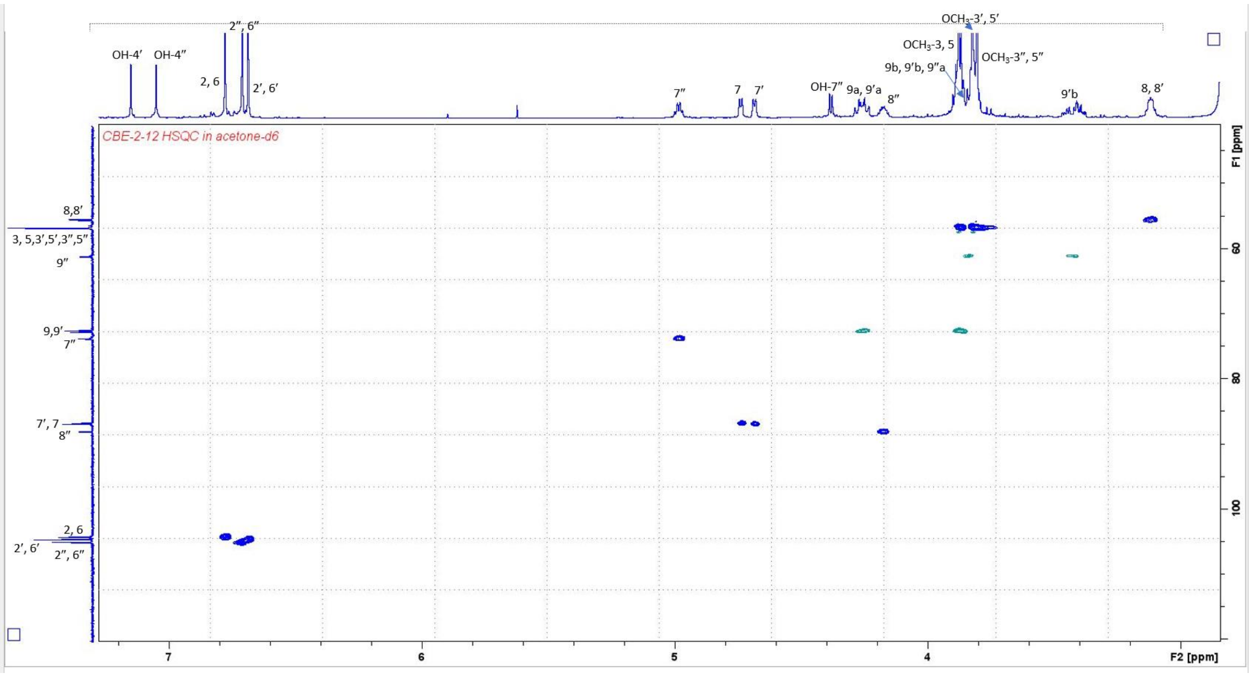

Figure S11: HSQC spectrum of (−)-(7″*R*,8″*S*)-buddlenol D (**1**), (100 MHz, acetone-d_6)

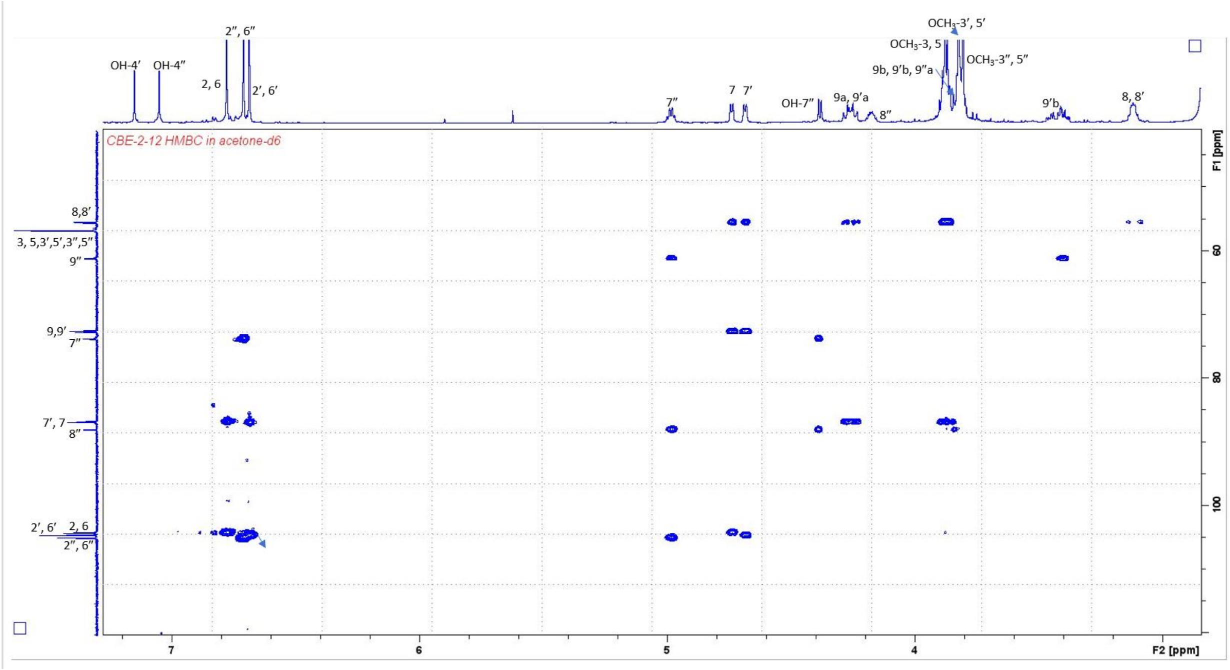

Figure S12: HMBC spectrum of (−)-(7″*R*,8″*S*)-buddlenol D (**1**), (100 MHz, acetone-d_6)

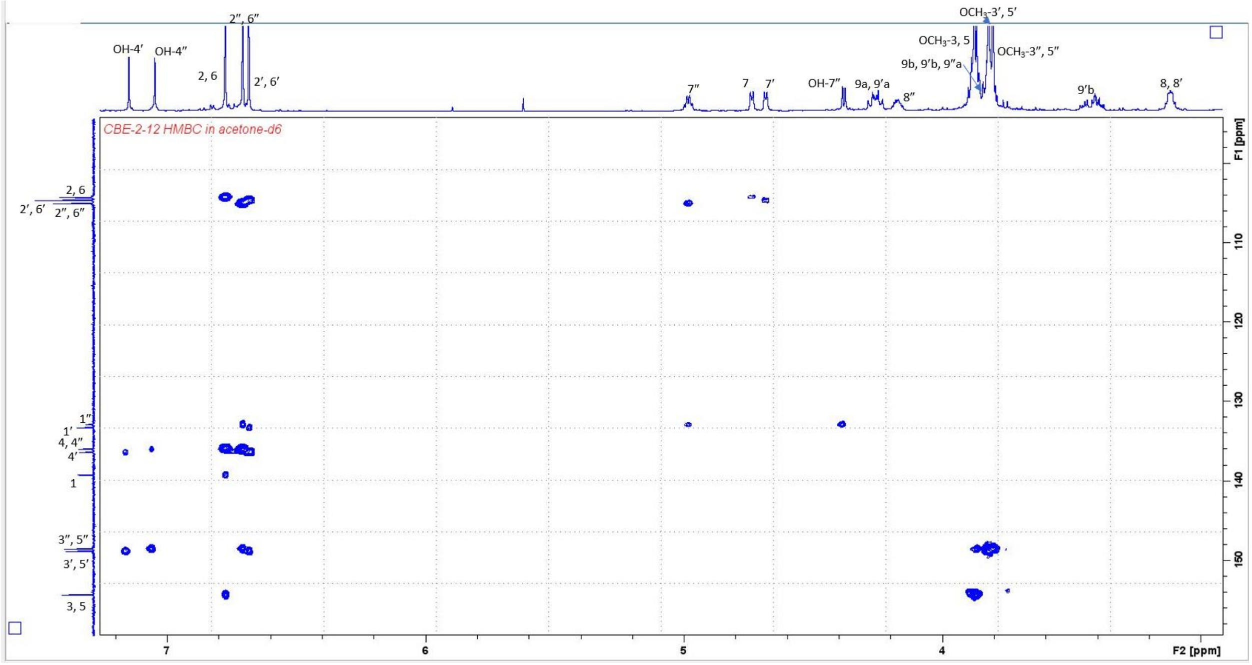

Figure S13: HMBC spectrum of (−)-(7″*R*,8″*S*)-buddlenol D (**1**), (100 MHz, acetone-d_6), (continued)

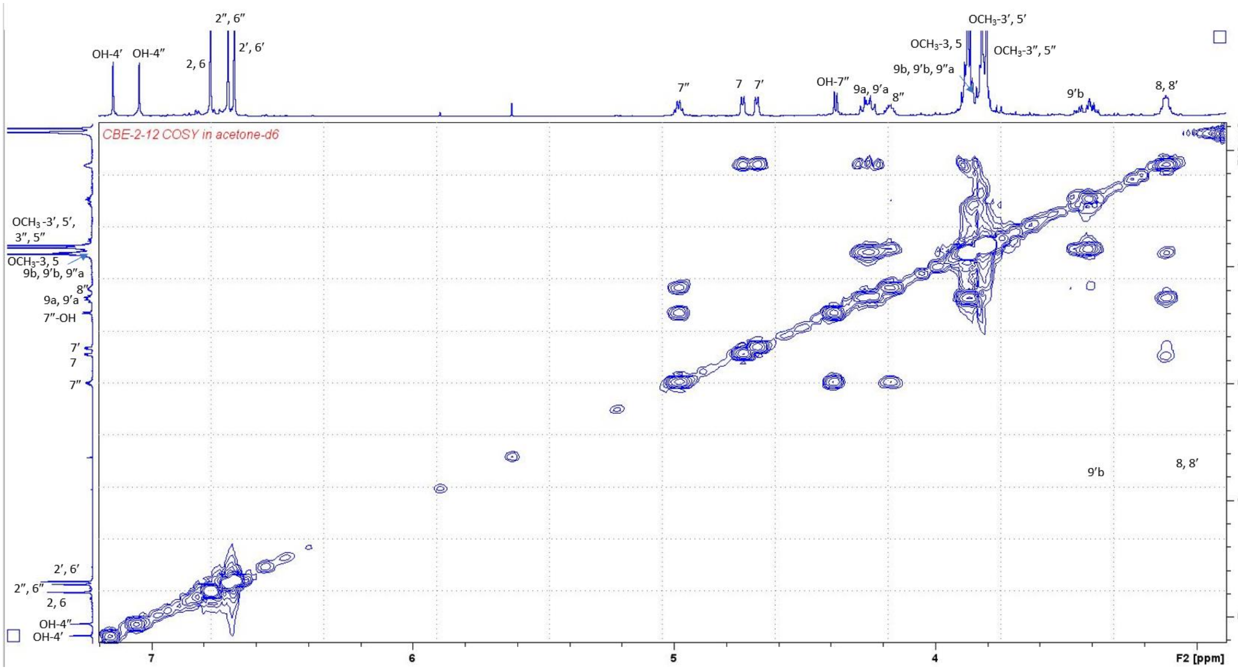

Figure S14: ^{1}H-^{1}H COSY spectrum of (−)-(7′′*R*,8′′*S*)-buddlenol D (**1**), (100 MHz, acetone-*d*$_6$)

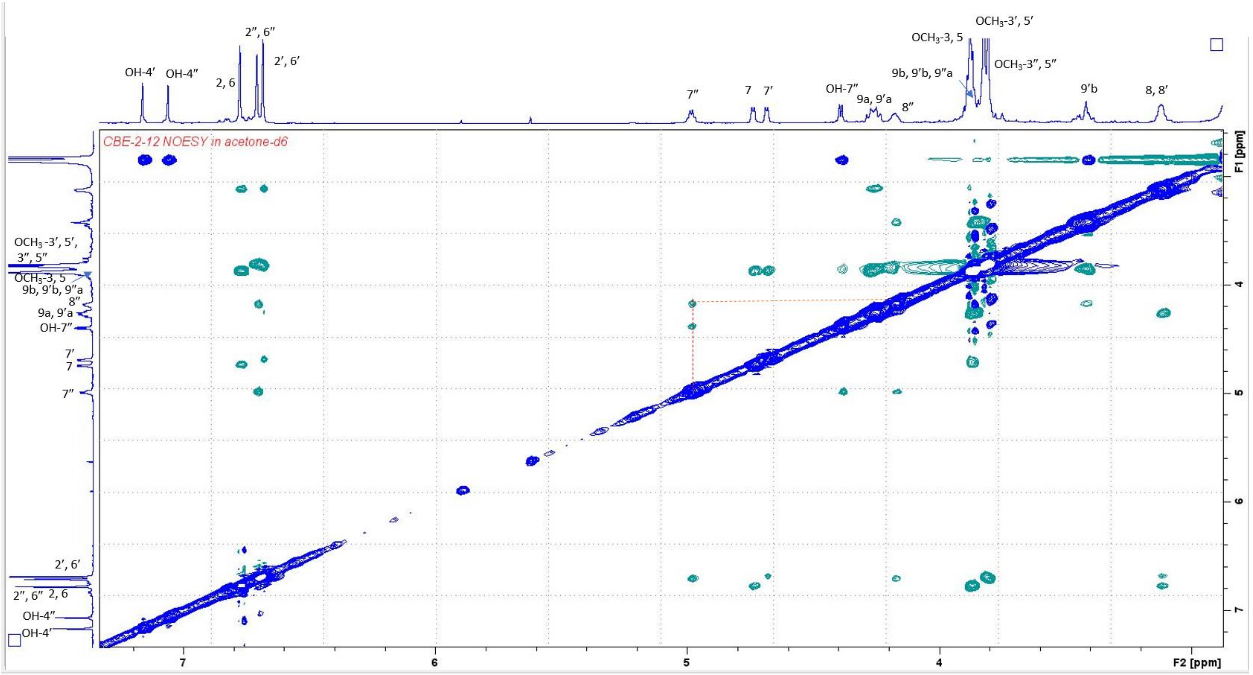

Figure S15: NOESY spectrum of (−)-(7′′*R*,8′′*S*)-buddlenol D (**1**), (100 MHz, acetone-*d*$_6$),

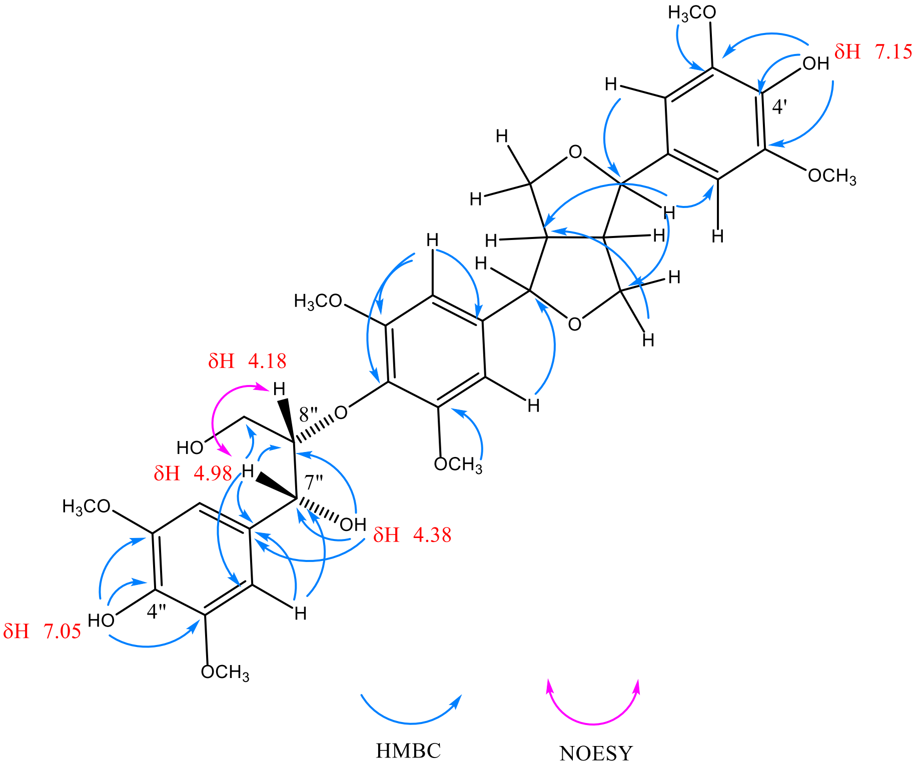

Figure S16: Selected HMBC and NOESY correlations of (−)-(7′′*R*,8′′*S*)-buddlenol D (**1**)

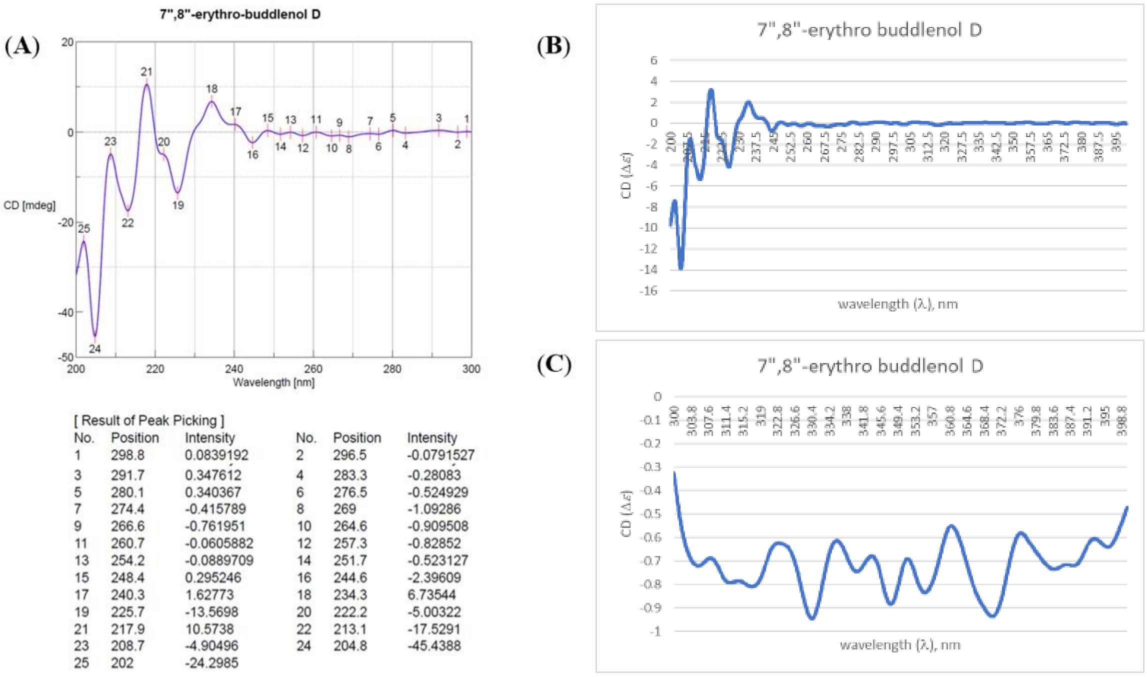

[Result of Peak Picking]

No.	Position	Intensity	No.	Position	Intensity
1	298.8	0.0839192	2	296.5	-0.0791527
3	291.7	0.347612	4	283.3	-0.28083
5	280.1	0.340367	6	276.5	-0.524929
7	274.4	-0.415789	8	269	-1.09286
9	266.6	-0.761951	10	264.6	-0.909508
11	260.7	-0.0605882	12	257.3	-0.82852
13	254.2	-0.0889709	14	251.7	-0.523127
15	248.4	0.295246	16	244.6	-2.39609
17	240.3	1.62773	18	234.3	6.73544
19	225.7	-13.5698	20	222.2	-5.00322
21	217.9	10.5738	22	213.1	-17.5291
23	208.7	-4.90496	24	204.8	-45.4388
25	202	-24.2985			

Figure S17: CD spectra of (−)-(7′′*R*,8′′*S*)-buddlenol D (**1**), (MeCN): (**A**) Y-axis : milli degree, (**B**) Y-axis : Δ*ε*, and (**C**) $Rh_2(OCOCF_3)_4$-induced CD spectrum, (CH_2Cl_2): Y-axis : Δ*ε*

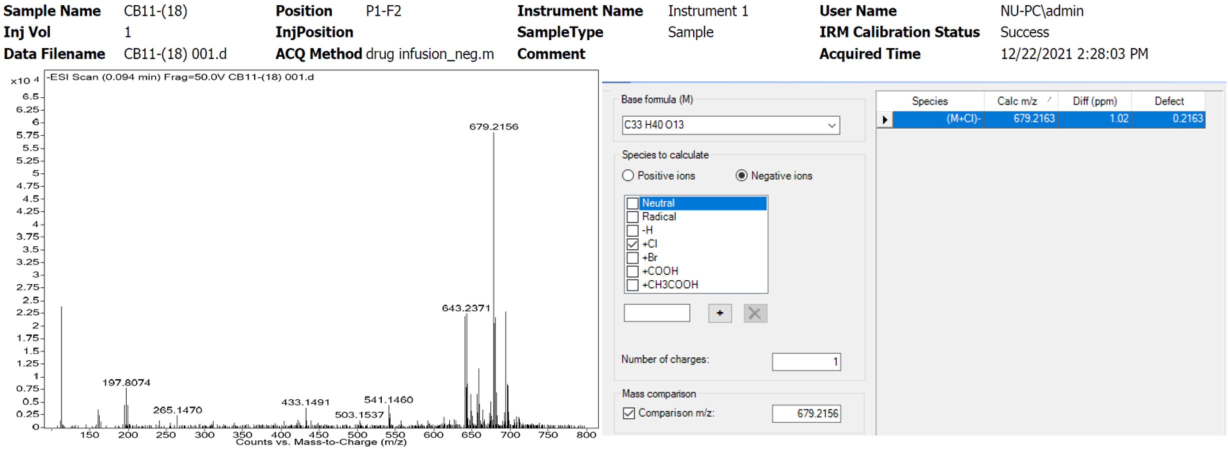

Figure S18: High resolution ESI-MS of (−)-(7″*S*,8″*S*)-buddlenol D (**2**), $[M+Cl]^-$ at *m/z* of 679.2156

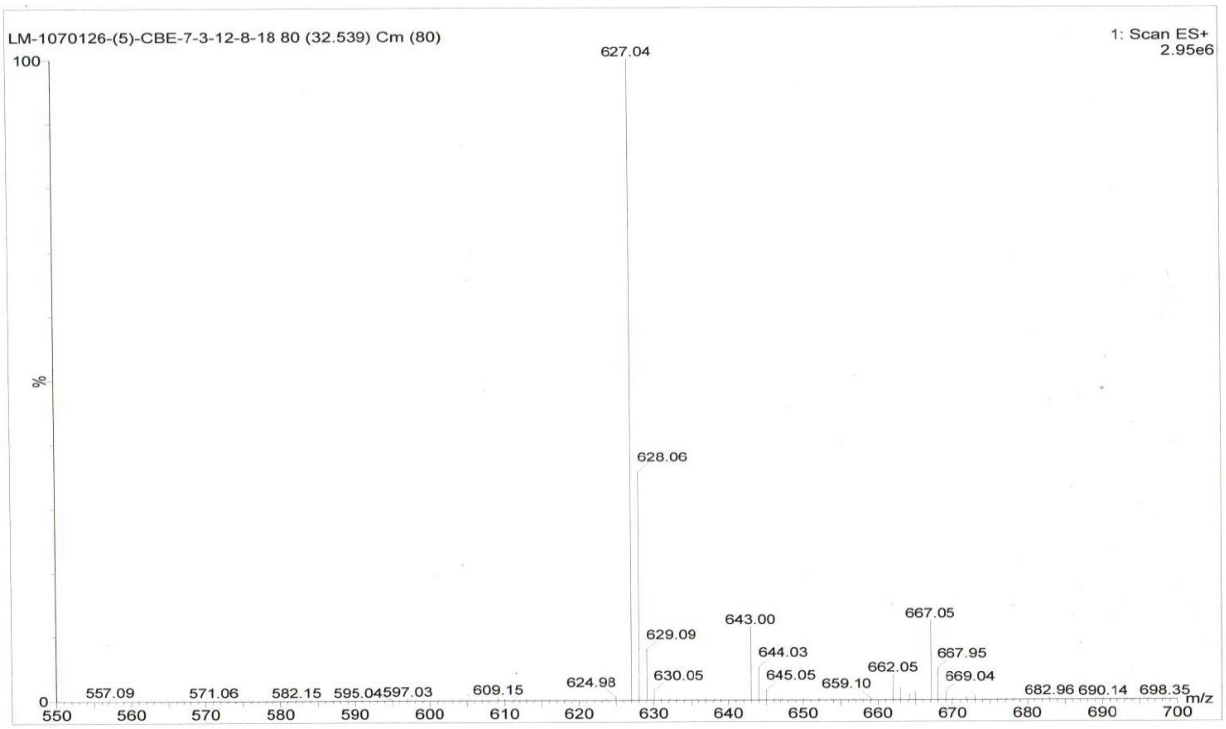

Figure S19: ESI-MS of (−)-(7″*S*,8″*S*)- buddlenol D (**2**), $[M+Na]^+$ *m/z* 667

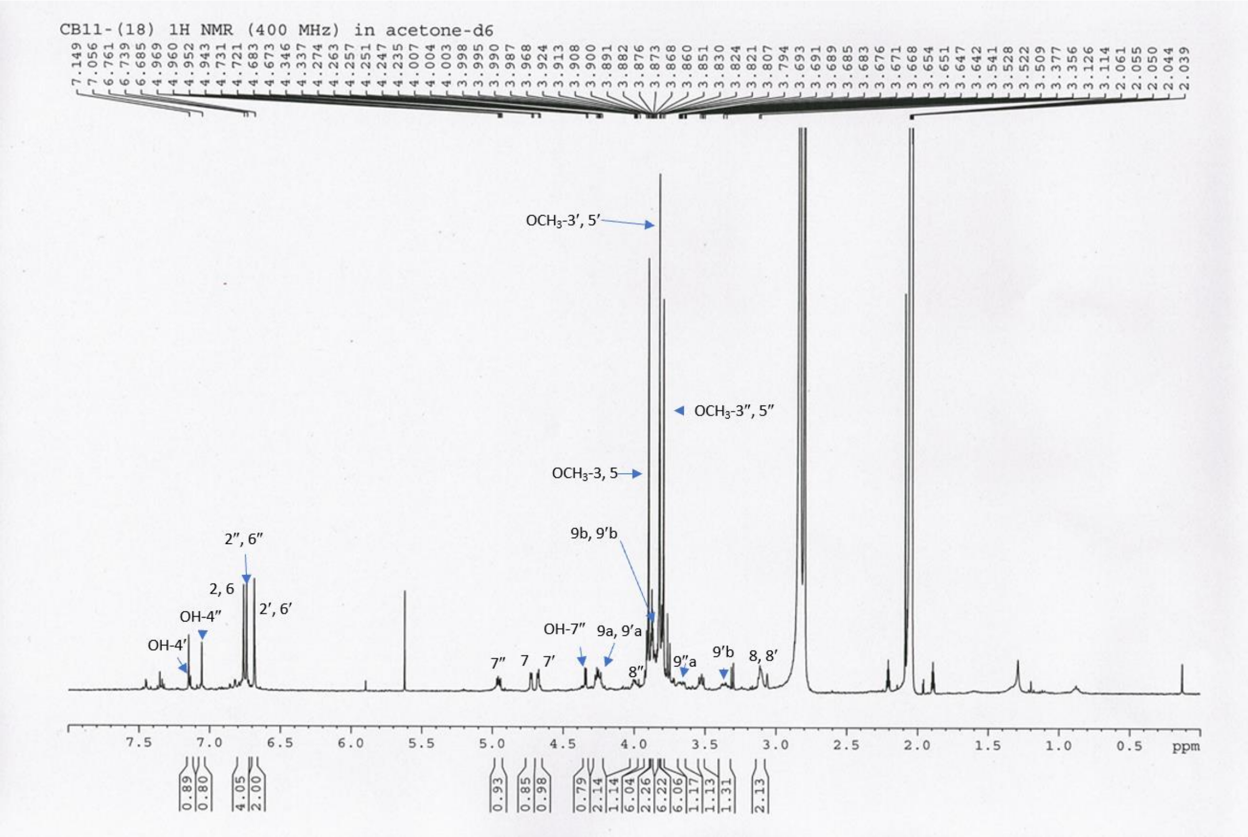

Figure S20: ^{1}H-NMR spectrum of (−)-(7″*S*,8″*S*)-buddlenol D (**2**), (400 MHz, acetone-d_6)

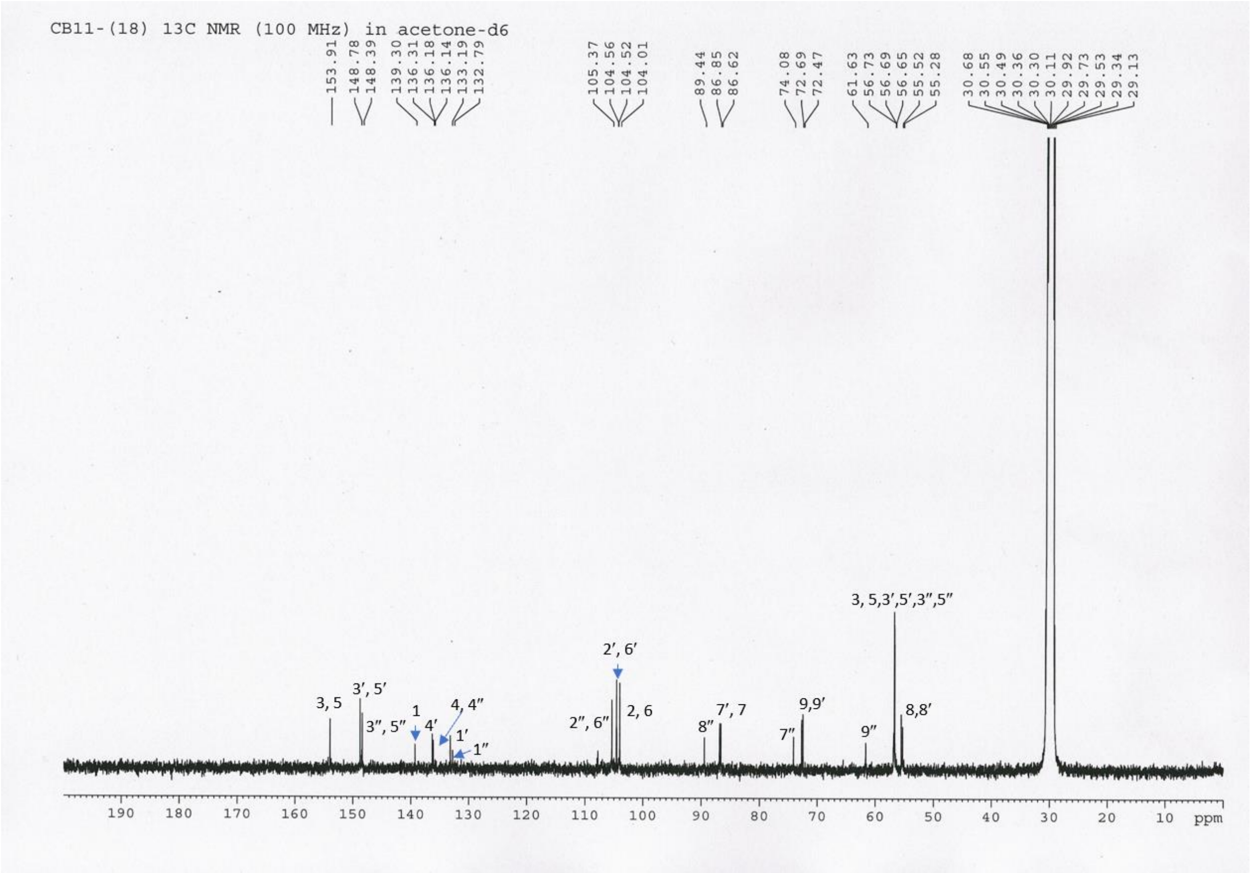

Figure S21: ^{13}C-NMR spectrum of (−)-(7″*S*,8″*S*)-buddlenol D (**2**), (100 MHz, acetone-*d*$_6$)

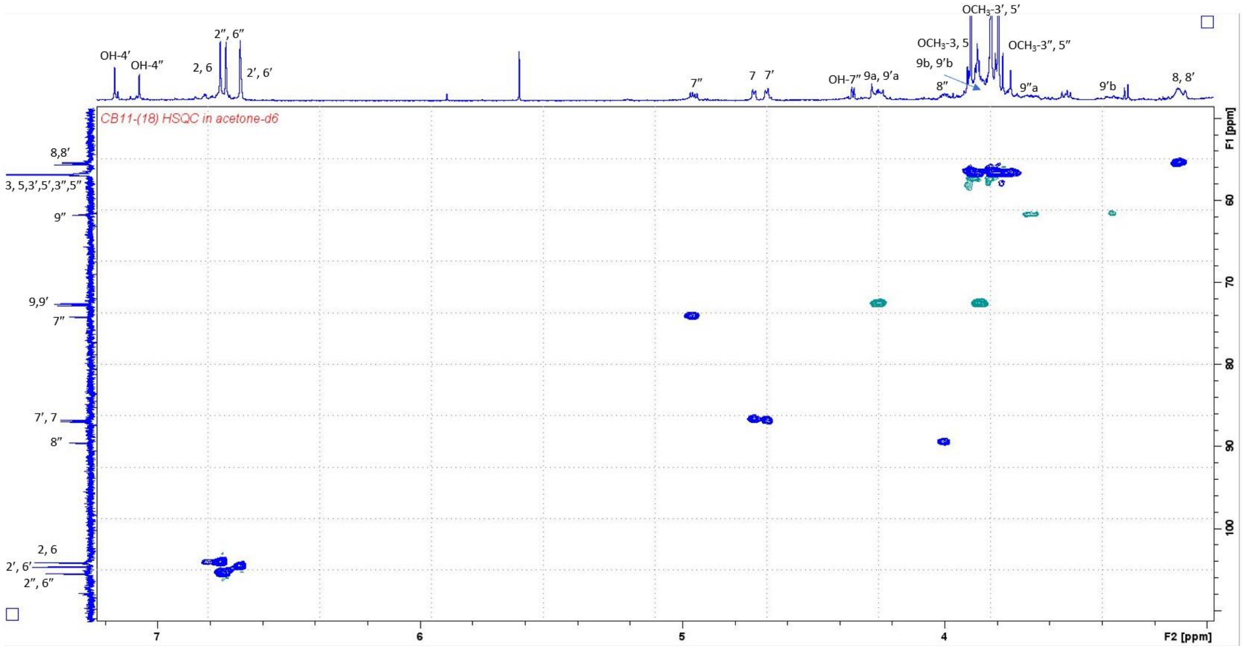

Figure S22: HSQC spectrum of (−)-(7″*S*,8″*S*)-buddlenol D (**2**), (100 MHz, acetone-*d*$_6$)

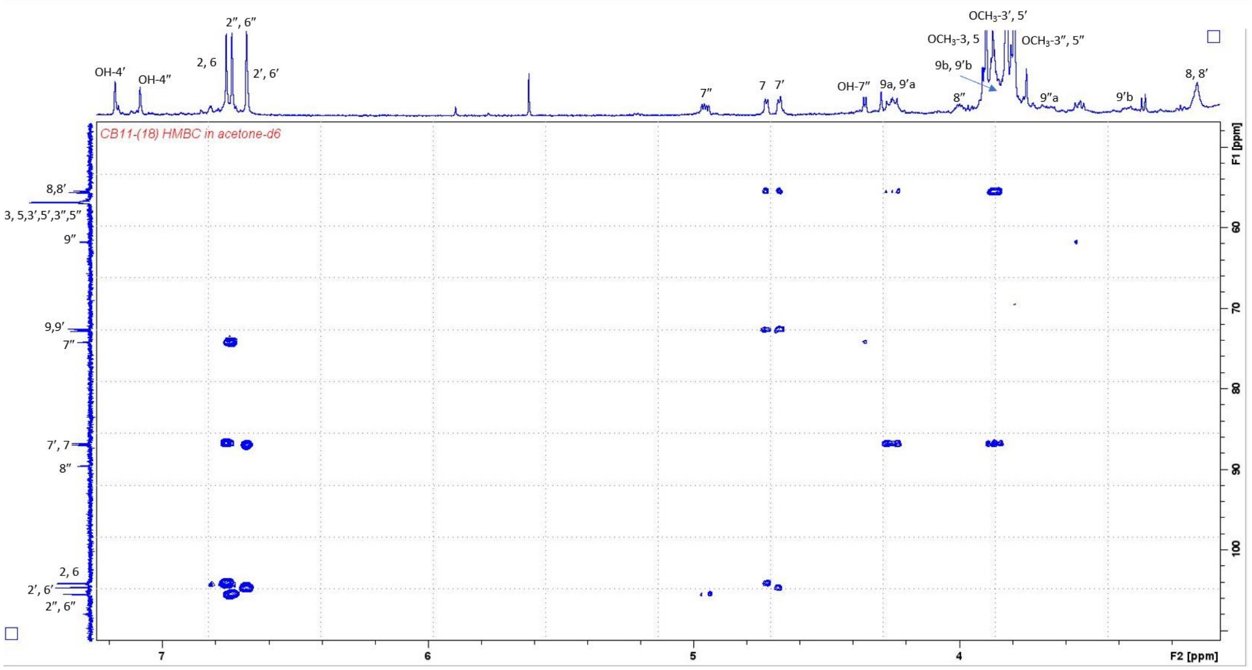

Figure S23: HMBC spectrum of (−)-(7″*S*,8″*S*)-buddlenol D (**2**), (100 MHz, acetone-d_6)

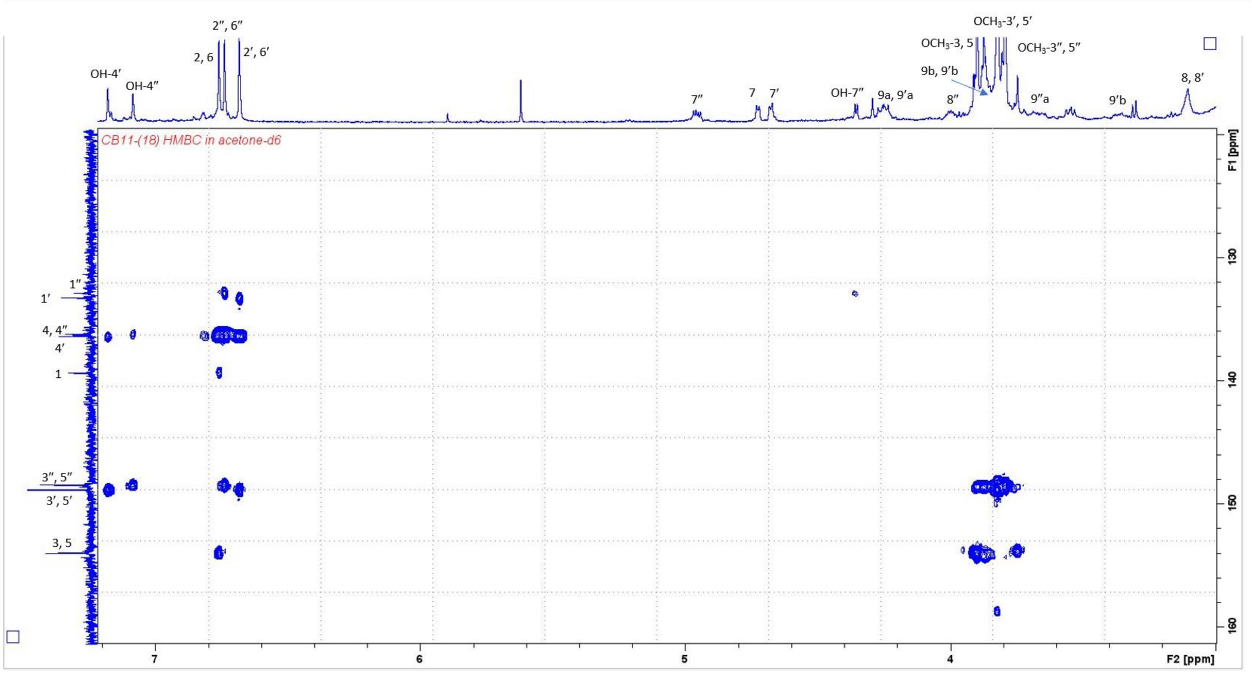

Figure S24: HMBC spectrum of (−)-(7″*S*,8″*S*)-buddlenol D (**2**), (100 MHz, acetone-d_6), (continued)

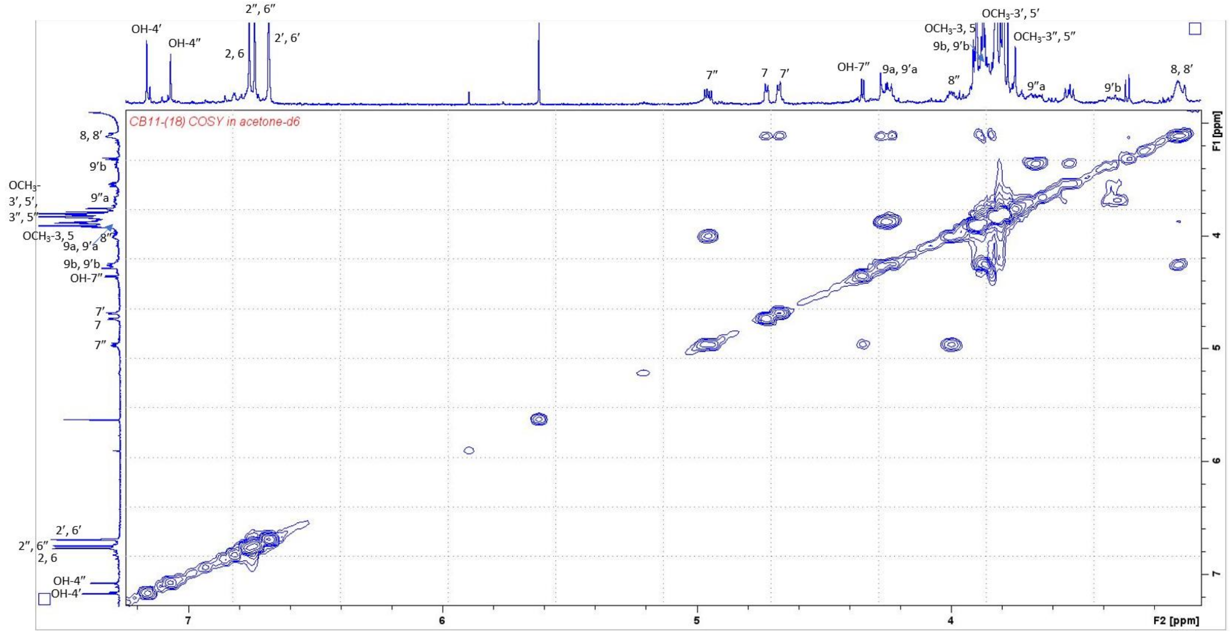

Figure S25: ^{1}H-^{1}H COSY spectrum of (−)-(7′′*S*,8′′*S*)-buddlenol D (**2**), (100 MHz, acetone-d_6)

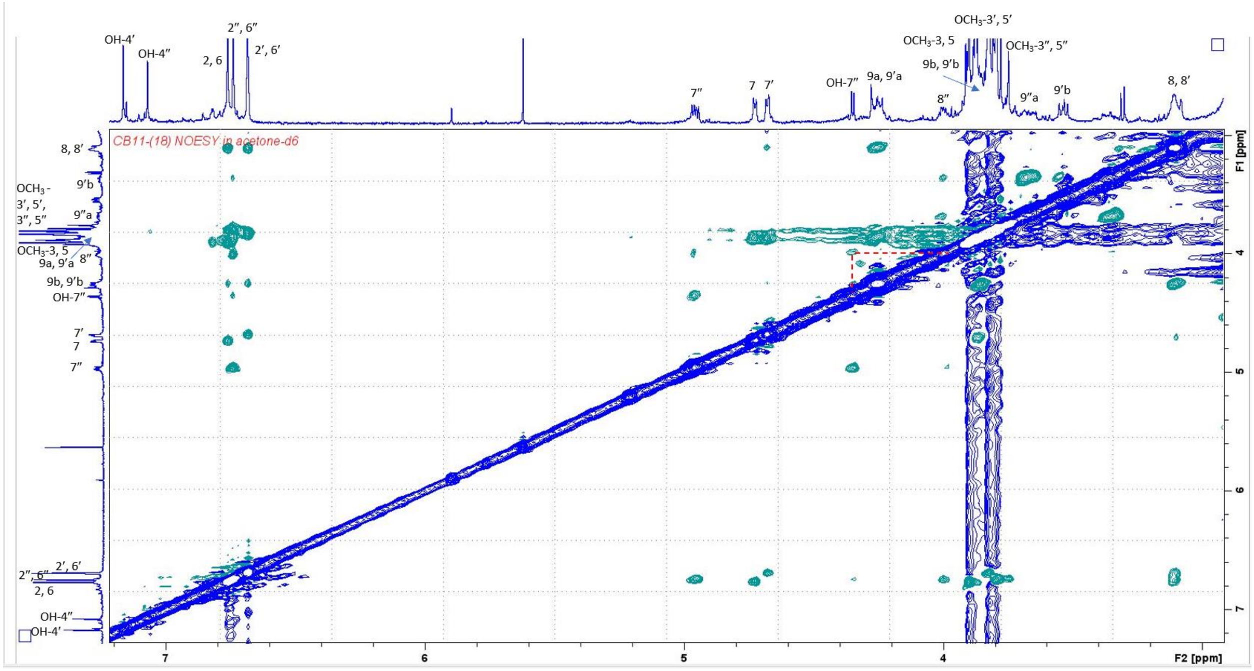

Figure S26: NOESY spectrum of (−)-(7′′*S*,8′′*S*)-buddlenol D (**2**), (100 MHz, acetone-d_6)

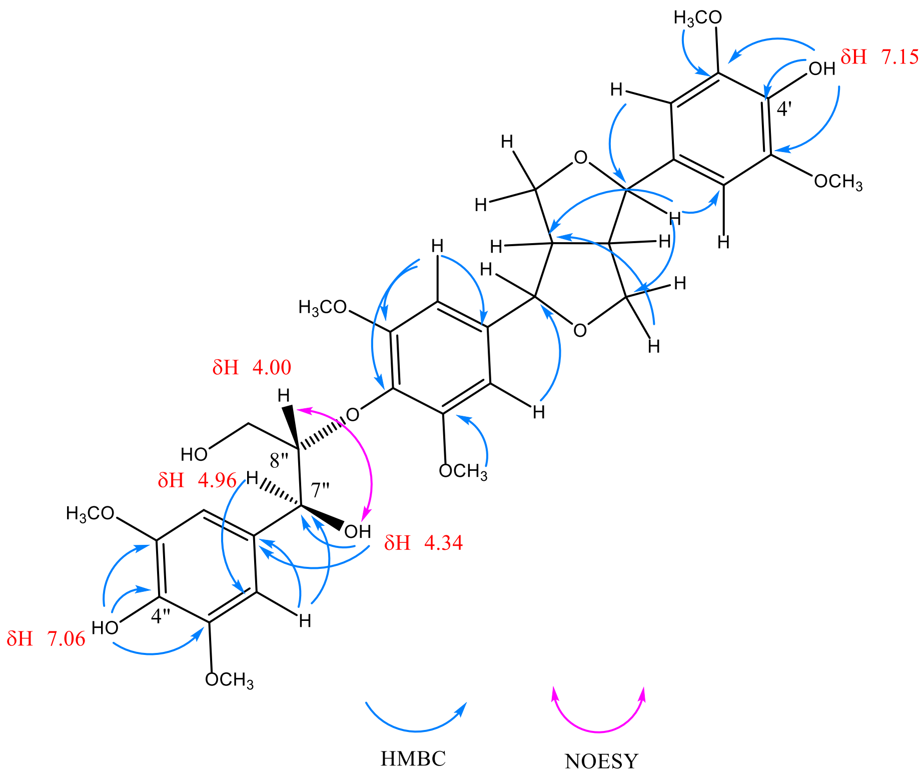

Figure S27: Selected HMBC and NOESY correlations of (−)-(7″*S*,8″*S*)-buddlenol D (**2**)

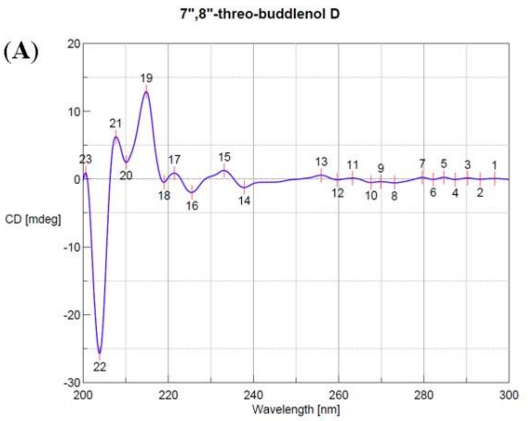

[Result of Peak Picking]

No.	Position	Intensity	No.	Position	Intensity
1	296.6	0.0512264	2	293.2	-0.0538078
3	290.3	0.126716	4	287.4	-0.0946122
5	284.7	0.224508	6	282.2	-0.0865591
7	279.7	0.206197	8	273.1	-0.586035
9	269.9	-0.412068	10	267.6	-0.531063
11	263.3	0.104969	12	259.7	-0.131848
13	255.9	0.540492	14	237.8	-1.26585
15	233.1	1.24671	16	225.5	-2.01109
17	221.4	0.854654	18	219	-0.488054
19	214.8	12.8931	20	210.1	2.43598
21	207.7	6.21192	22	203.9	-25.7475
23	200.6	0.899951			

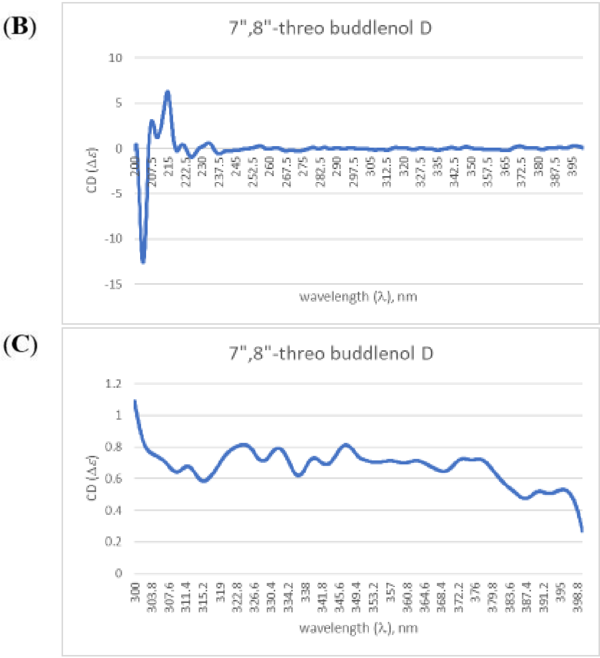

Figure S28: CD spectra of (−)-(7″*S*,8″*S*)- buddlenol D (**2**), (MeCN): (**A**) Y-axis : milli degree, (**B**) Y-axis : Δ*ε*, and (**C**) $Rh_2(OCOCF_3)_4$-induced CD spectrum, (CH_2Cl_2): Y-axis : Δ*ε*.

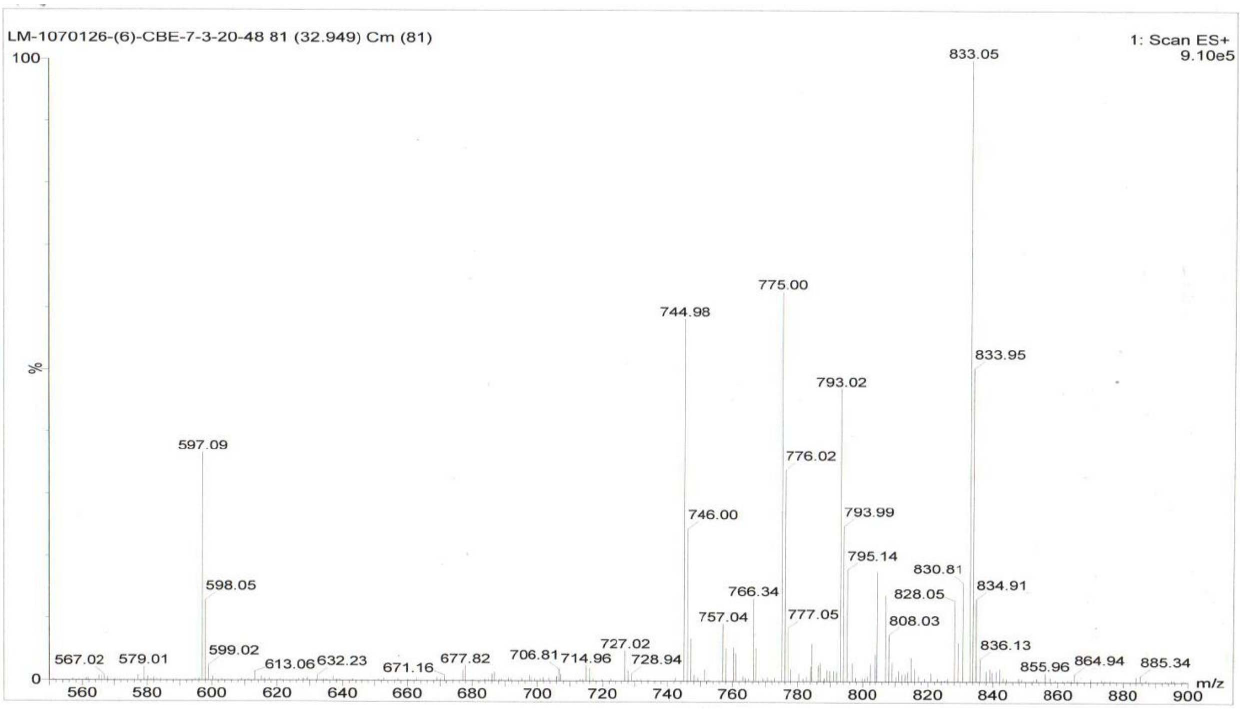

Figure S29: ESI-MS of (+)-7″*R*,8″*S*:7‴*R*,8‴*S*-hedyotisol A (**3**), [M+Na]$^+$ *m*/*z* 833

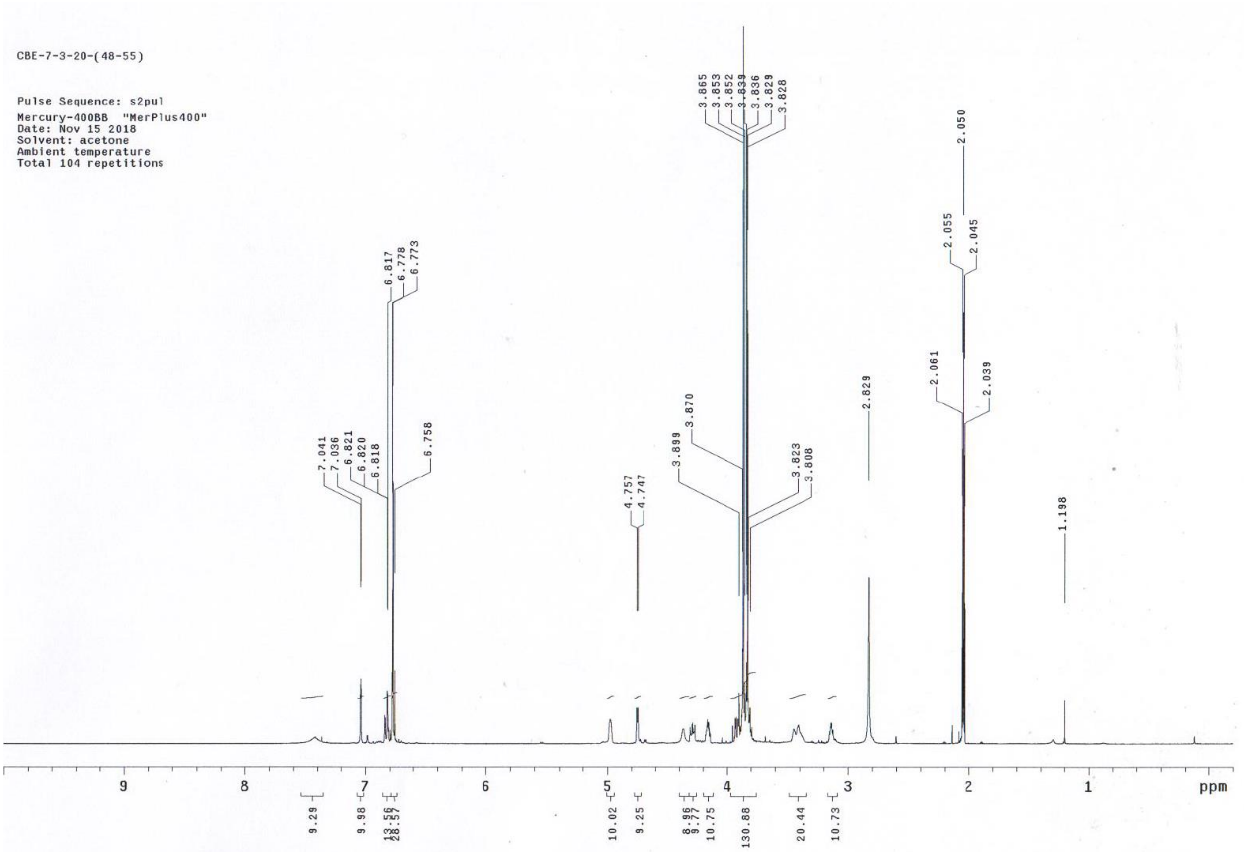

Figure S30: ^{1}H-NMR spectrum of (+)-7″*R*,8″*S*:7‴*R*,8‴*S*-hedyotisol A (**3**), (400 MHz, acetone-d_6)

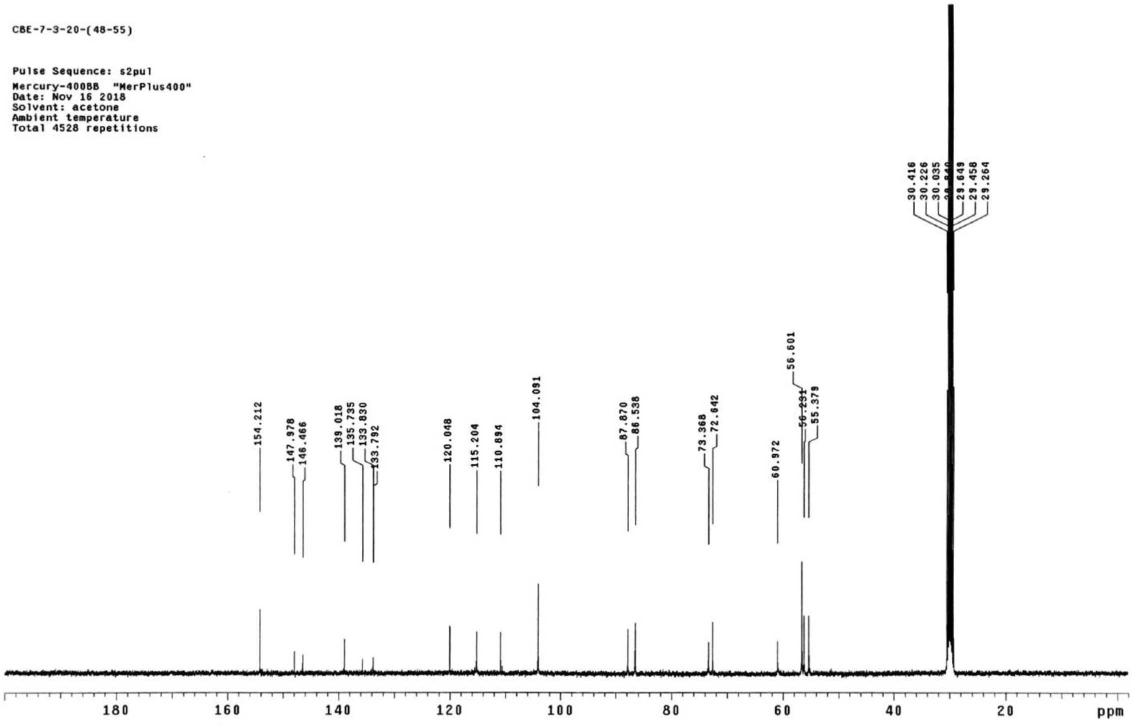

Figure S31: ^{13}C-NMR spectrum of (+)-7″*R*,8″*S*:7‴*R*,8‴*S*-hedyotisol A (**3**), (100 MHz, acetone-d_6)

(A)

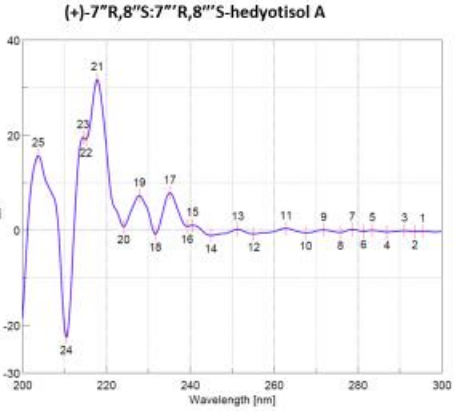

No.	Position	Intensity	No.	Position	Intensity
1	295.4	-0.186606	2	293.5	-0.311325
3	291.1	-0.121414	4	286.9	-0.395515
5	283.4	-0.0243537	6	281.3	-0.231928
7	278.6	0.107917	8	275.8	-0.478755
9	271.8	0.0188826	10	267.5	-0.5631
11	262.8	0.370029	12	255.1	-0.789339
13	251.3	0.165777	14	244.9	-1.10092
15	240.5	1.01149	16	239.2	0.837814
17	235.1	7.84533	18	231.6	-0.839622
19	227.8	7.28944	20	224.1	0.749185
21	217.8	31.6584	22	215.1	19.0569
23	214.4	19.4803	24	210.4	-22.5176
25	203.7	15.6586			

(B) (C)

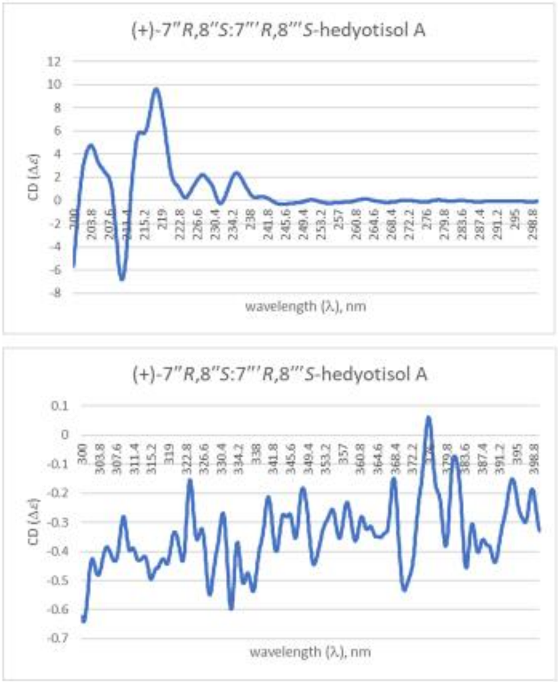

Figure S32: CD spectra of (+)-7″*R*,8″*S*:7‴*R*,8‴*S*-hedyotisol A (**3**), (MeCN): (**A**) Y-axis : milli degree, (**B**) Y-axis : Δ*ε*, and (**C**) $Rh_2(OCOCF_3)_4$-induced CD spectrum, (CH_2Cl_2): Y-axis : Δ*ε*. CD nm (MeCN): 203.7 (Δ*ε* +4.75), 210.4 (Δ*ε* –6.84), 214.4 (Δ*ε* +5.91), 217.8 (Δ*ε* +9.61), 224.1 (Δ*ε* +0.23), 227.8 (Δ*ε* +2.21), 231.6 (Δ*ε* –0.26), 235.1 (Δ*ε* +2.38), 240.5 (Δ*ε* +0.31), 244.9 (Δ*ε* –0.33), 281.3 (Δ*ε* –0.07). $Rh_2(OCOCF_3)_4$-induced CD nm (CH_2Cl_2): 350.7 (Δ*ε* –0.45). $[\alpha]^{20}_D$: +2.88° (*c* 1.25×10^{-4} g/mL, MeOH)

Reference for **3** :

Xiong, L. *et al.* Lignans and neolignans from *Sinocalamus affinis* and their absolute configurations. *Journal of Natural Products* **74**, 1188-1200, doi:10.1021/np200117y (2011).

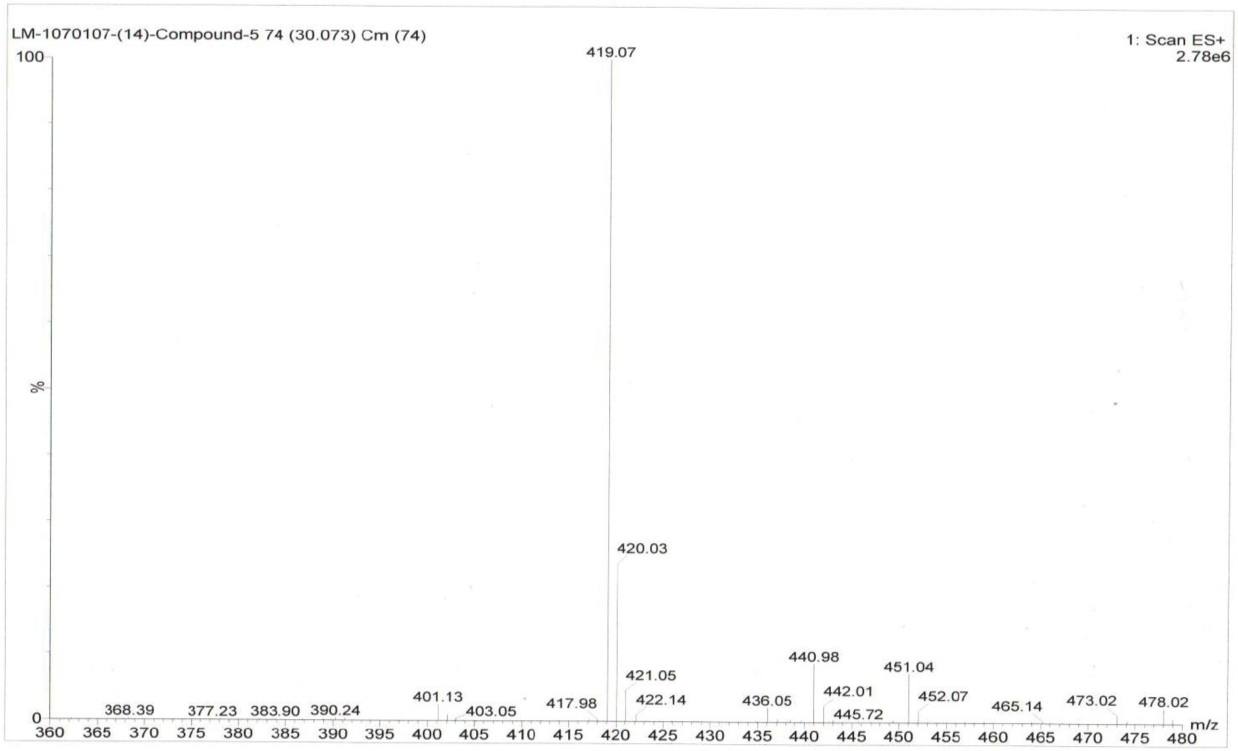

Figure S33: ESI-MS of (–)-syringaresinol (**4**), $[M+H]^+$ *m*/*z* 419

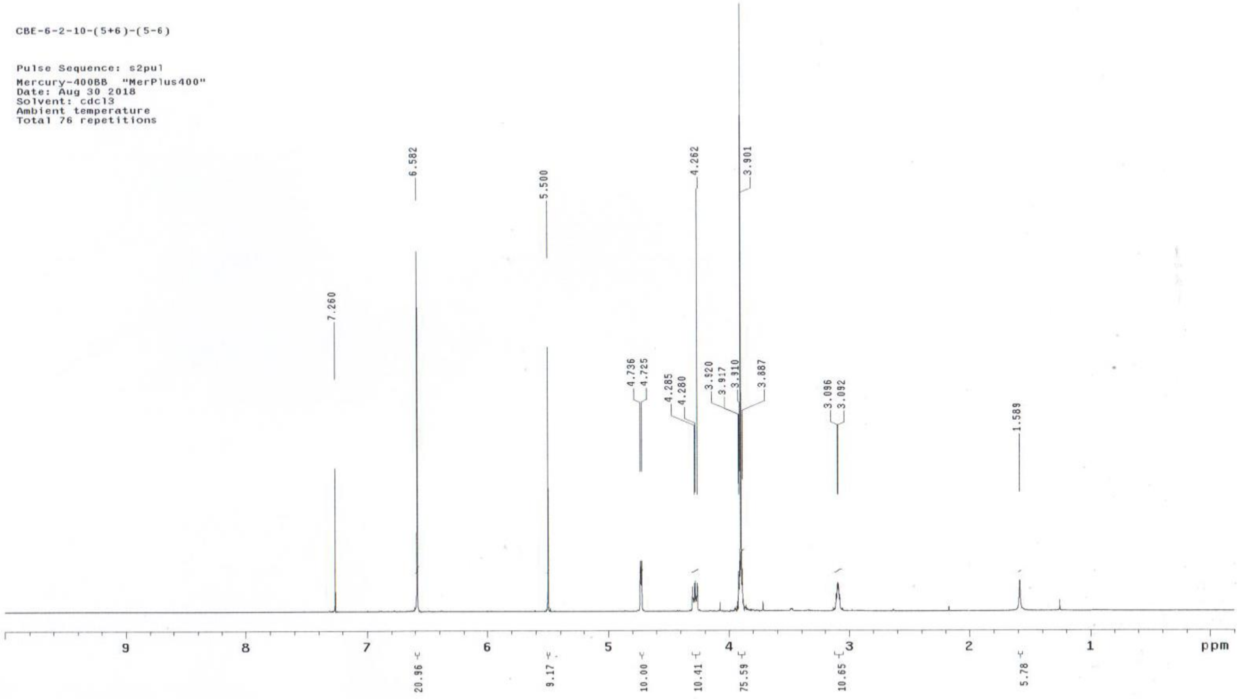

Figure S34: ^{1}H-NMR spectrum of (–)-syringaresinol (**4**), (400 MHz, $CDCl_3$)

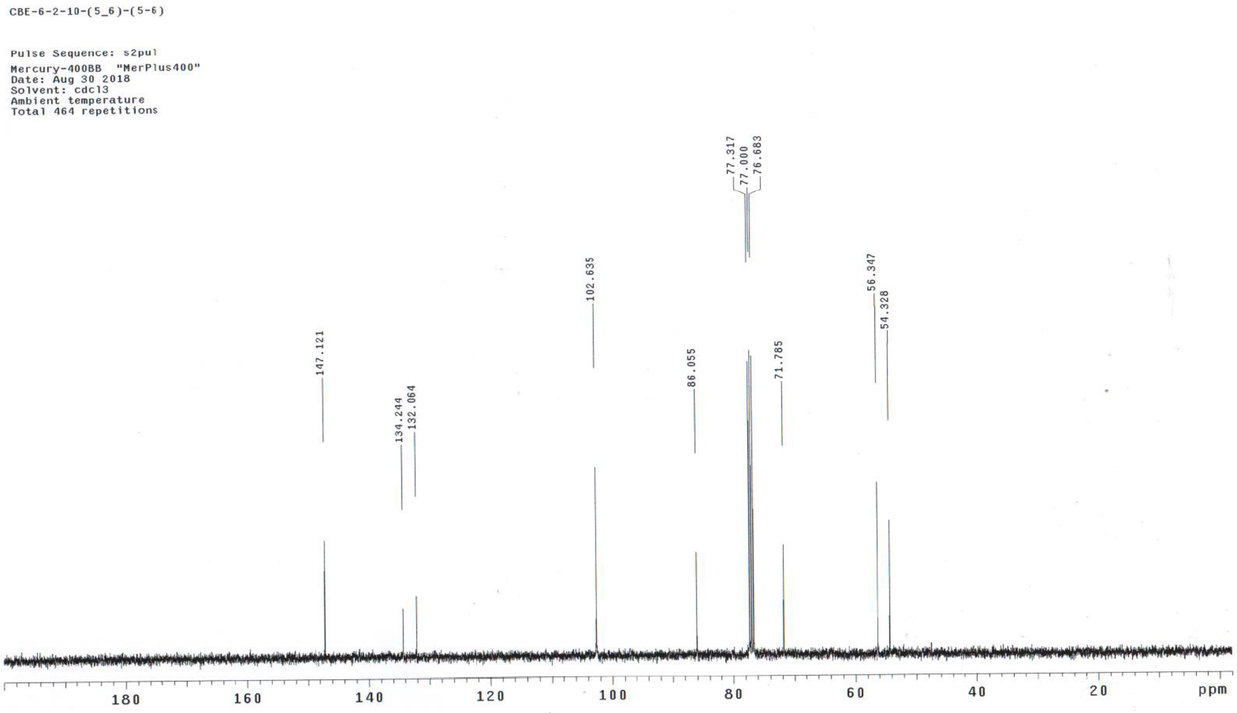

Figure S35: ^{13}C-NMR spectrum of (–)-syringaresinol (**4**), (100 MHz, $CDCl_3$)

(A)

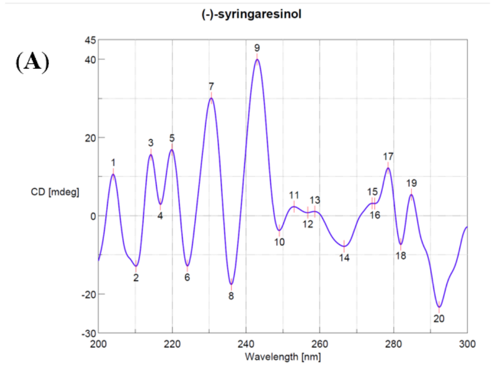

No.	Position	Intensity	No.	Position	Intensity
1	204	10.5587	2	210.2	-12.9382
3	214.2	15.6041	4	216.8	2.86159
5	219.9	16.8565	6	224.1	-12.8759
7	230.6	30.0401	8	236	-17.6021
9	243	39.9183	10	249	-3.81281
11	253	2.25721	12	256.7	0.753546
13	258.6	1.03854	14	266.6	-7.86732
15	274.2	3.11664	16	274.9	3.07984
17	278.5	12.1558	18	281.9	-7.38224
19	284.8	5.35748	20	292.3	-23.4055

(B)

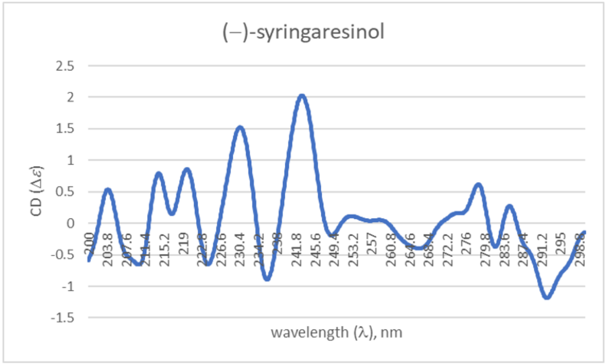

Figure S36: CD spectra of (−)-syringaresinol (**4**), (MeCN): (**A**) Y-axis : milli degree, (**B**) Y-axis : Δ*ε*.

CD nm (MeOH): 204.0 (Δ*ε* +0.54), 210.2 (Δ*ε* −0.66), 214.2 (Δ*ε* +0.79), 216.8 (Δ*ε* +0.15), 219.9 (Δ*ε* +0.86), 224.1 (Δ*ε* −0.65), 230.6 (Δ*ε* +1.53), 236.0 (Δ*ε* −0.89), 243 (Δ*ε* +2.03), 266.6 (Δ*ε* −0.40), 278.5 (Δ*ε* +0.62), 281.9 (Δ*ε* −0.38), 284.8 (Δ*ε* +0.27), 292.3 (Δ*ε* −1.19). $[\alpha]^{20}_{D}$: −26.0° (*c* 5.0×10^{-5} g/mL, MeOH)

References for **4** :

Maihesuti, L., Lan, P., Imerhasan, M., Eshbakova, K. & Jia, X. A new spiro compound from Caragana acanthophylla. *Chemistry of Natural Compounds* **53**, 646-648 (2017).

Wu, Y.-C., Chang, G.-Y., Ko, F.-N. & Teng, C.-M. Bioactive constitutents from the stems of Annona montana. *Planta medica* **61**, 146-149 (1995).

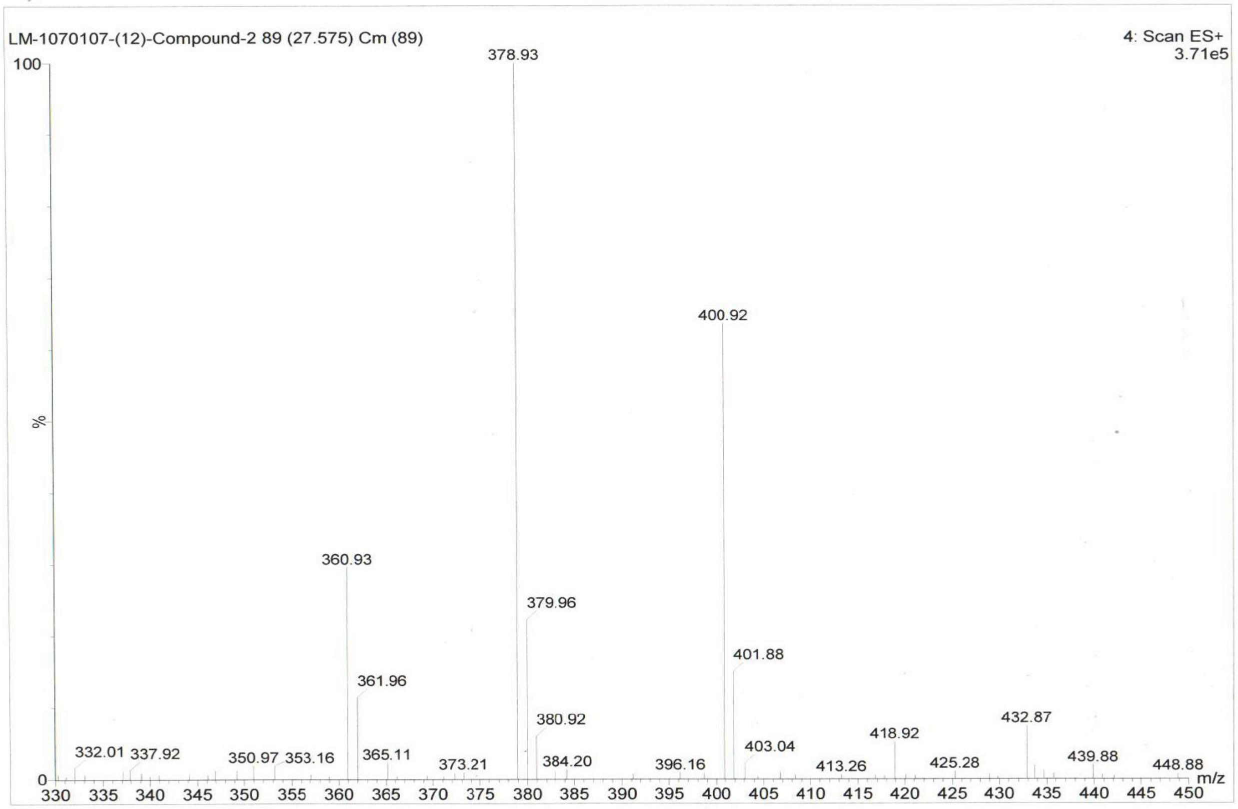

Figure S37: ESI-MS of (+)-diptoindonesin D (**5**), $[M+H]^+$ *m/z* 379

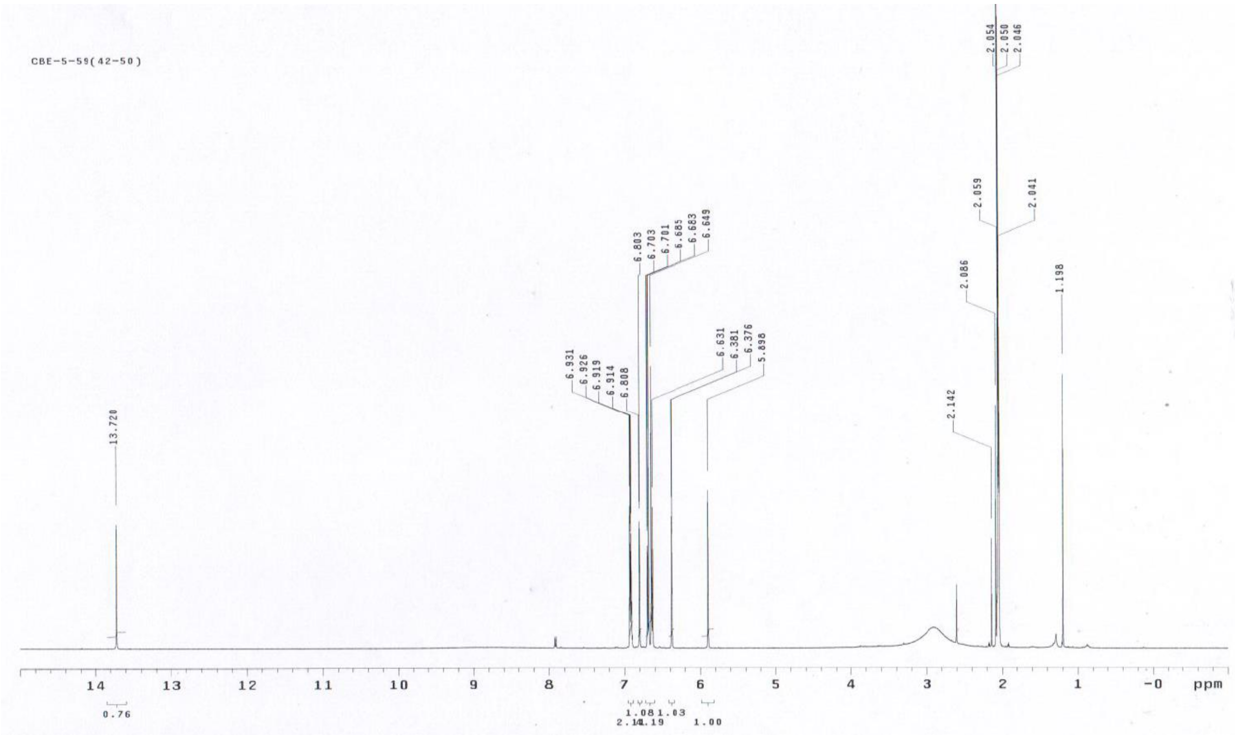

Figure S38: ^{1}H-NMR spectrum of (+)-diptoindonesin D (**5**), (500 MHz, acetone-d_6)

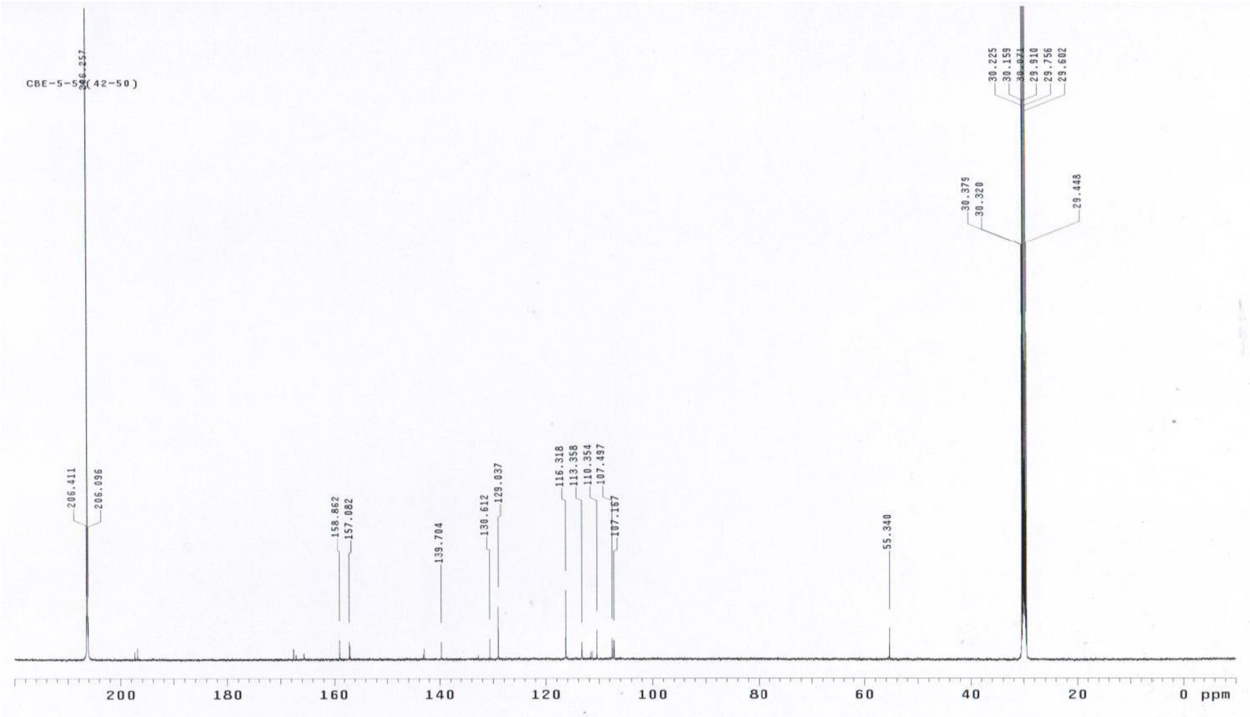

Figure S39: ^{13}C-NMR spectrum of (+)-diptoindonesin D (**5**), (125 MHz, acetone-d_6)

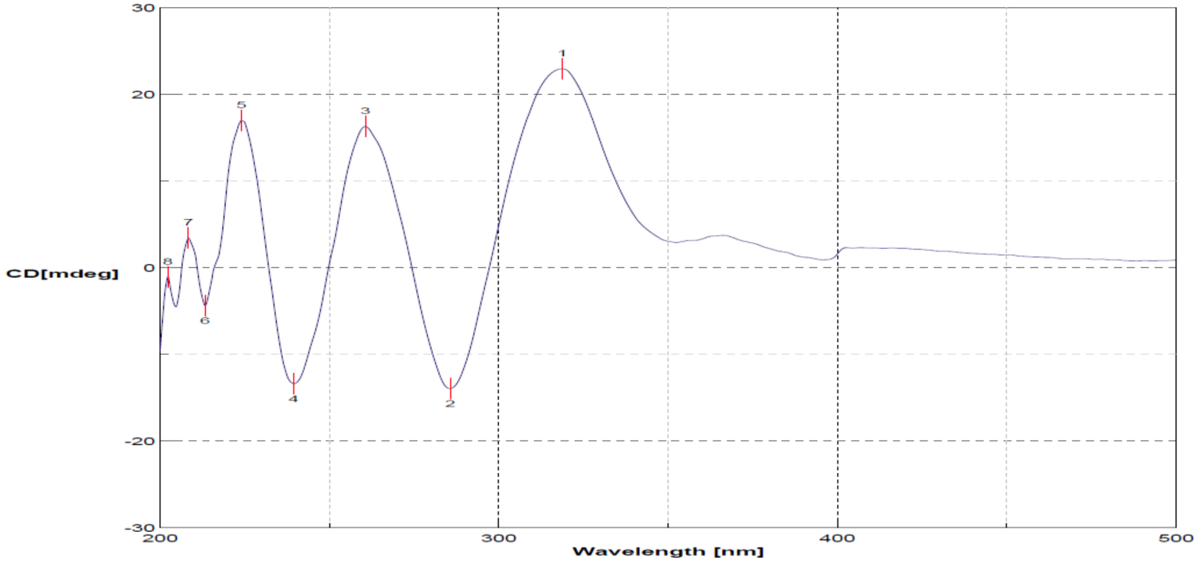

Figure S40: CD spectrum of (+)-diptoindonesin D (**5**), (MeOH), [Y-axis : milli degree]

CD nm (MeOH): 208.4 ($\Delta\varepsilon$ +1.18), 213.3 ($\Delta\varepsilon$ –1.51), 224.1 ($\Delta\varepsilon$ +5.84), 239.6 ($\Delta\varepsilon$ –4.62), 260.7 ($\Delta\varepsilon$ +5.61), 285.7 ($\Delta\varepsilon$ –4.81), 318.8 ($\Delta\varepsilon$ +7.89). $[\alpha]^{23}_{D}$: + 438.6° (c 1.0×10^{-4} g/mL, MeOH)

Reference for **5** :

Sahidin *et al.* Cytotoxic properties of oligostilbenoids from the tree barks of *Hopea dryobalanoides*. *Z Naturforsch C J Biosci* **60**, 723-727, doi:10.1515/znc-2005-9-1011 (2005).

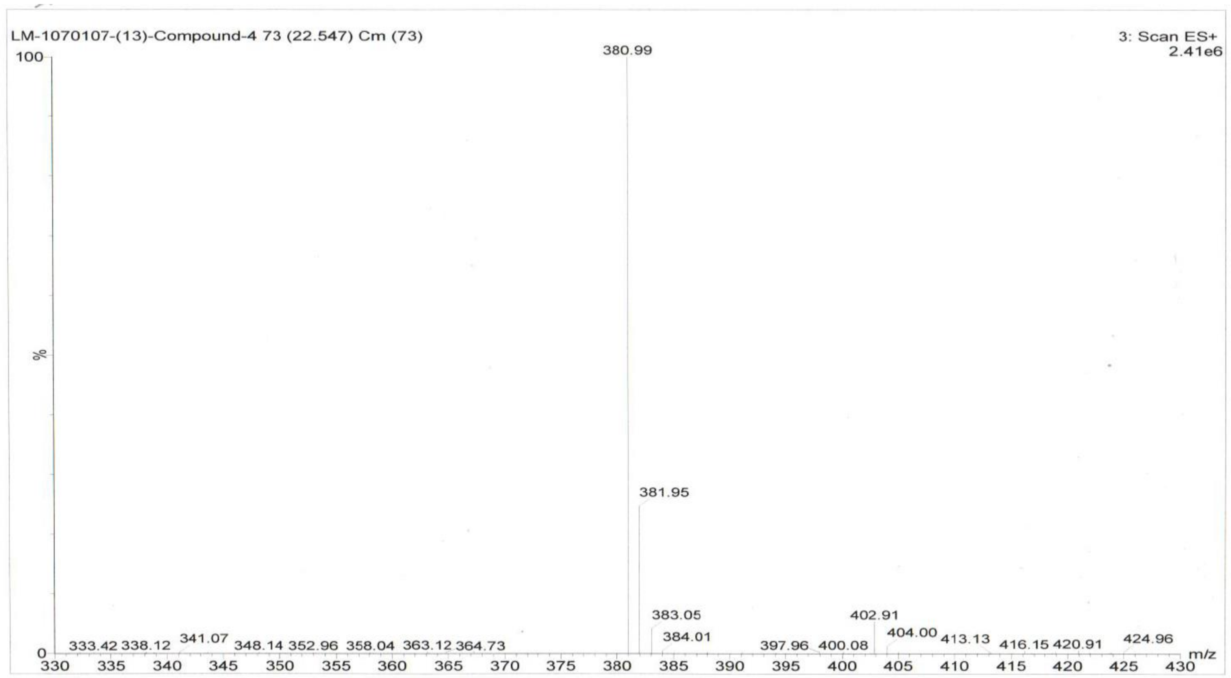

Figure S41: ESI-MS of (+)-parviflorol (**6**), $[M+H]^+$ *m/z* 381

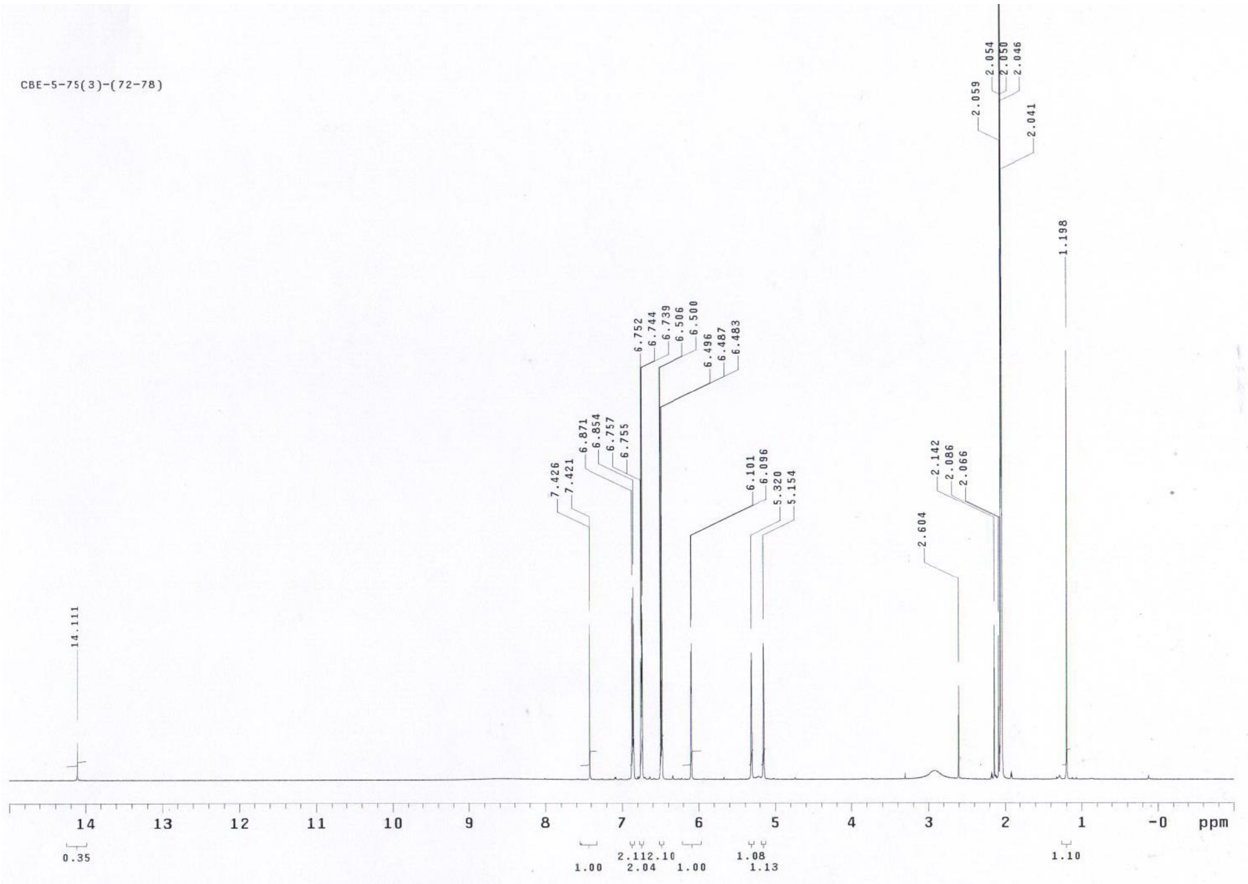

Figure S42: ^{1}H-NMR spectrum of (+)-parviflorol (**6**), (500 MHz, acetone-d_6)

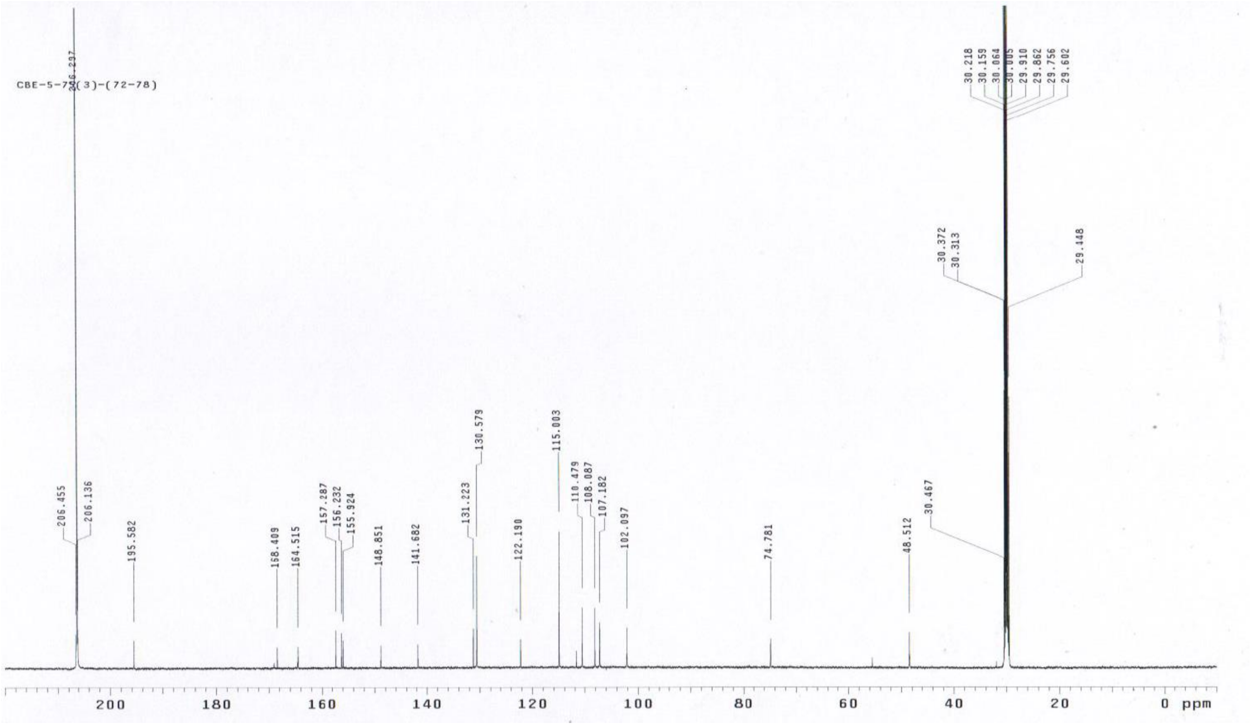

Figure S43: ^{13}C-NMR spectrum of (+)-parviflorol (**6**), (125 MHz, acetone-d_6)

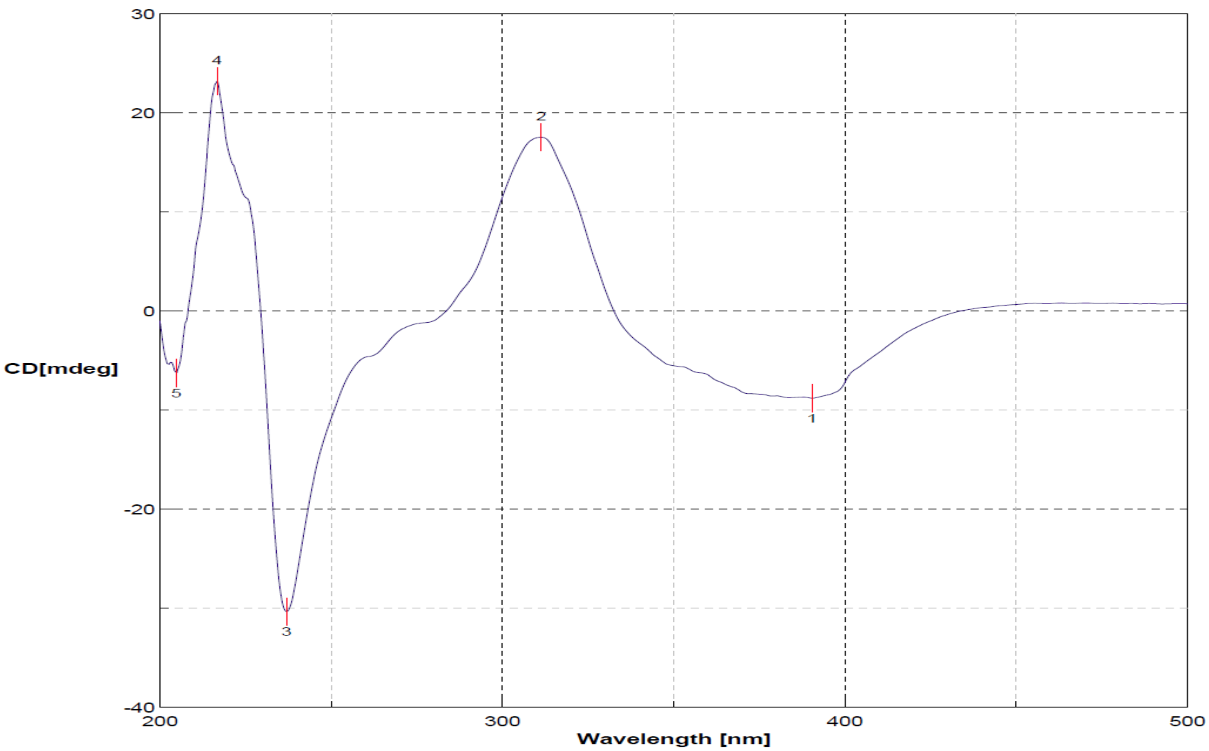

Figure S44: CD spectrum of (+)-parviflorol (**6**), (MeOH), [Y-axis : milli degree]

CD nm (MeOH): 202.5 ($\Delta\varepsilon$ –1.56), 204.9 ($\Delta\varepsilon$ +1.22), 207.7 ($\Delta\varepsilon$ –3.76), 220.1 ($\Delta\varepsilon$ +6.41), 236.8 ($\Delta\varepsilon$ –10.53), 311.3 ($\Delta\varepsilon$ +6.02), 386.8 ($\Delta\varepsilon$ –3.10). $[\alpha]^{23}_{D}$: + 264.7° (*c* 2.0×10^{-4} g/mL, MeOH)

Reference for **6** :

Tanaka, T. *et al.* Stilbenoids in the stem bark of *Hopea parviflora*. *Phytochemistry* **53**, 1015-1019, doi:10.1016/s0031-9422(00)00019-4 (2000).

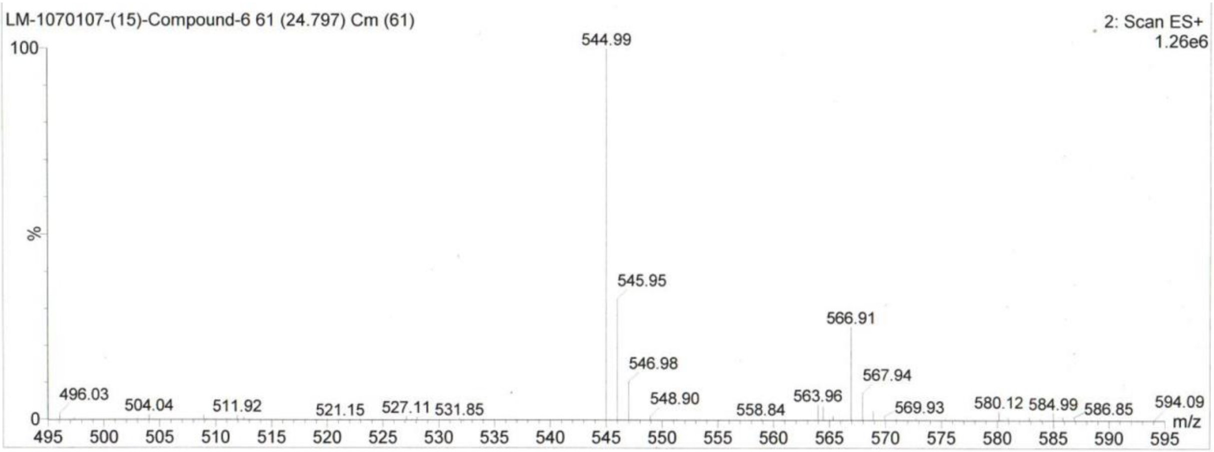

Figure S45: ESI-MS of (–)-mahuanin A (**7**), $[M+H]^+$ *m/z* 545

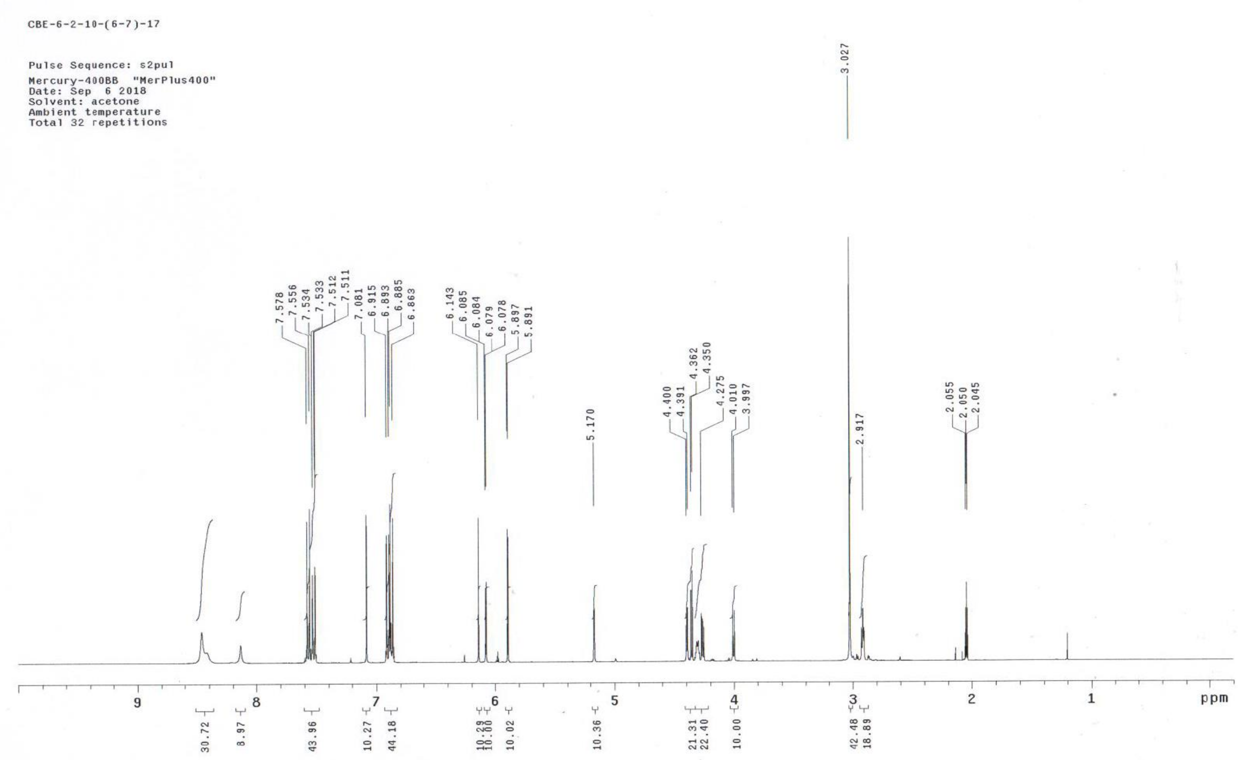

Figure S46: ^{1}H-NMR spectrum of (–)-mahuanin A (**7**), (400 MHz, acetone-d_6)

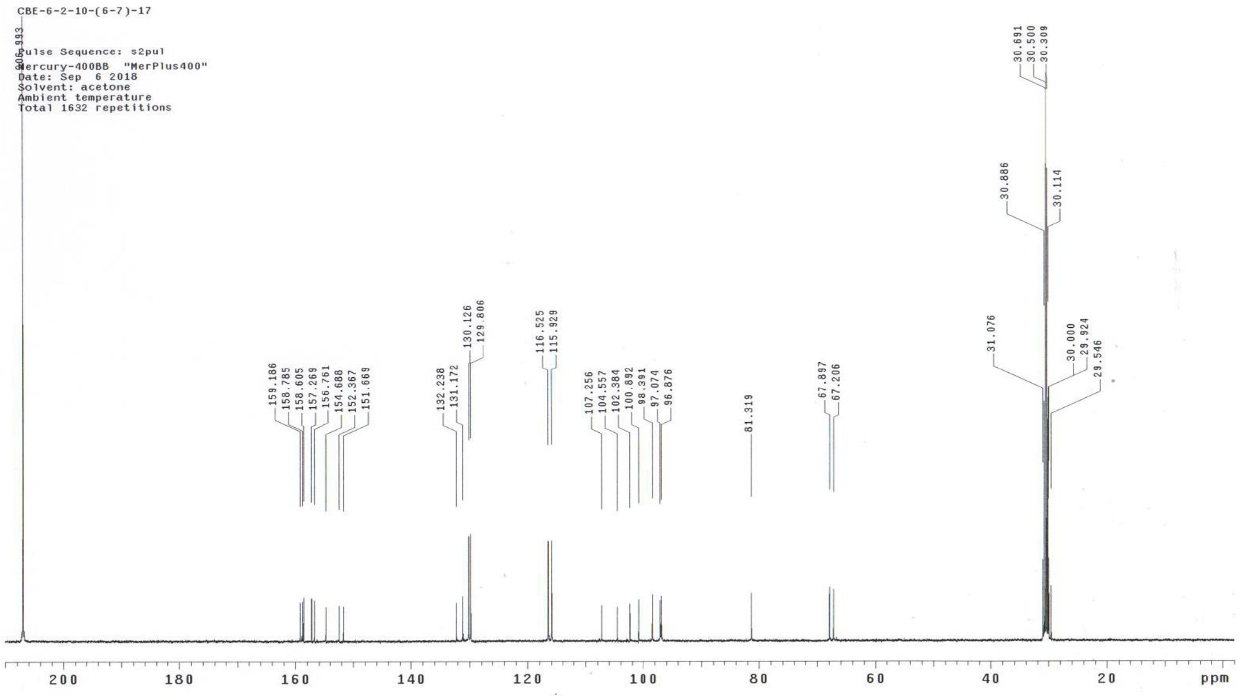

Figure S47: ^{13}C-NMR spectrum of (–)-mahuanin A (**7**), (100 MHz, acetone-d_6)

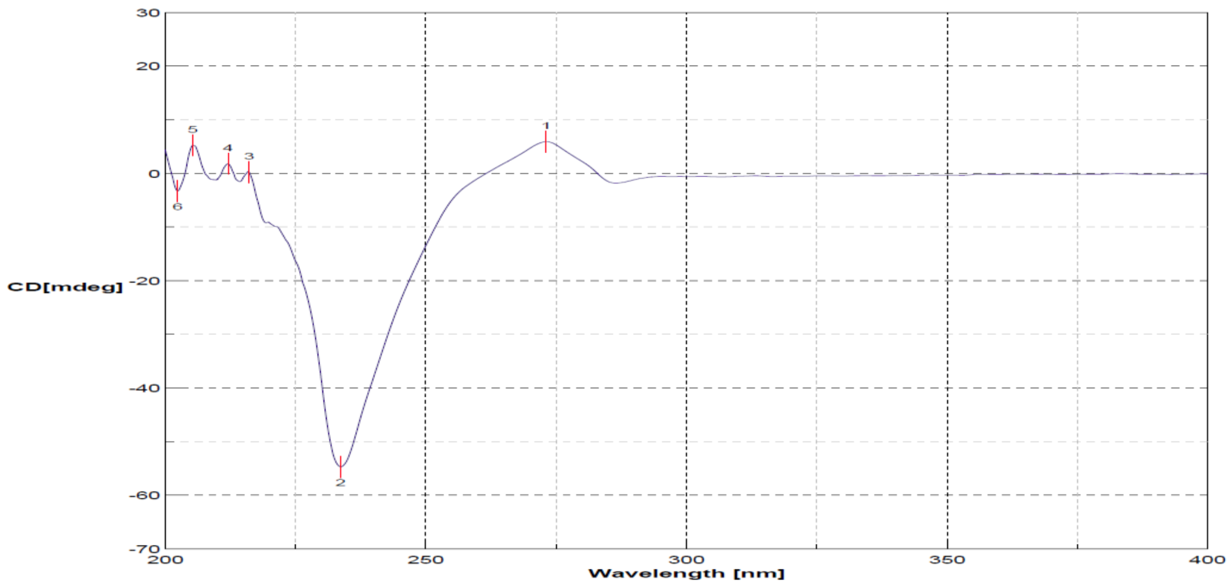

Figure S48: CD spectrum of (–)-mahuanin A (**7**), (MeOH), [Y-axis : milli degree]

CD nm (MeOH): 202.4 (Δ*ε* –1.63), 205.3 (Δ*ε* +2.59), 212.1 (Δ*ε* +0.90), 216.1 (Δ*ε* +0.11), 233.7 (Δ*ε* –27.11), 273.1 (Δ*ε* +2.93). $[\alpha]^{23}_{D}$: – 235.9° (*c* 4.0×10^{-4} g/mL, MeOH)

Reference for **7** :

Rawat, M. S. M., Prasad, D., Joshi, R. K. & Pant, G. Proanthocyanidins from *Prunus armeniaca* roots. *Phytochemistry* **50**, 321-324, doi:https://doi.org/10.1016/S0031-9422(98)00541-X (1999).

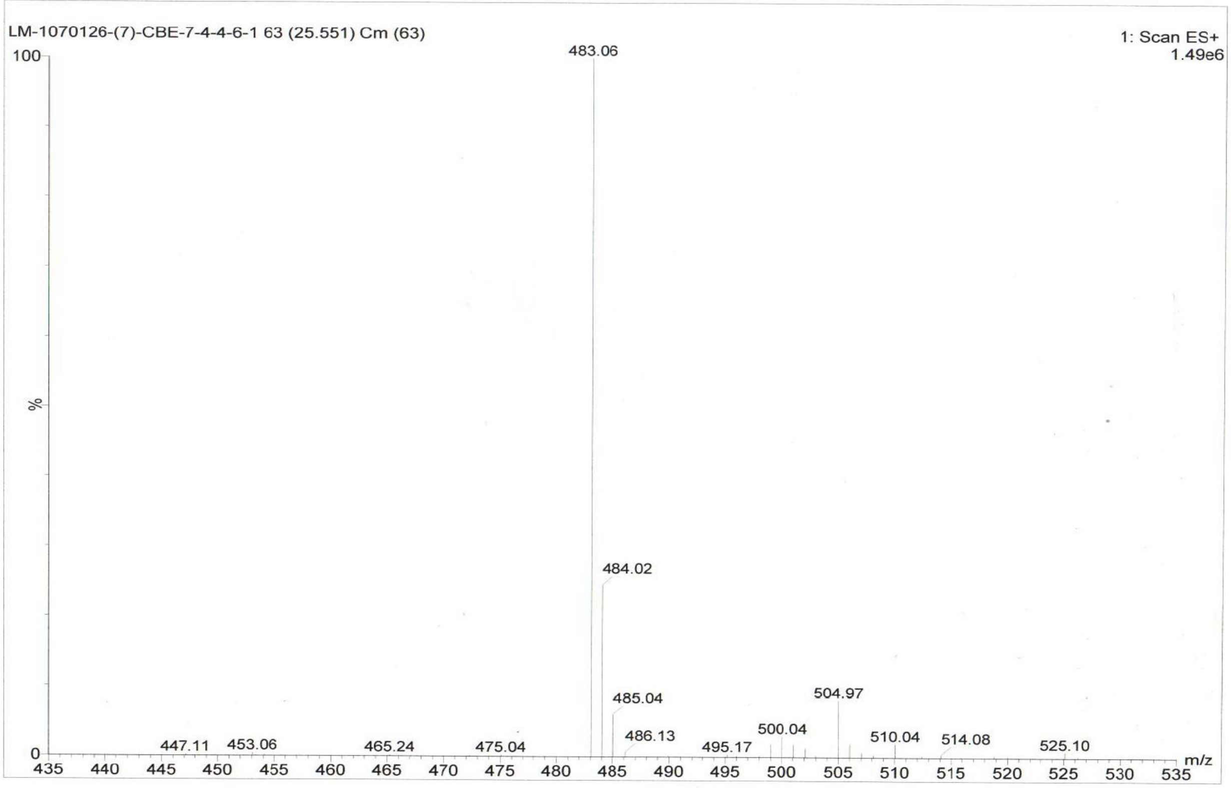

Figure S49: ESI-MS of 4-hydroxy-2-methoxyphenyl-6-*O*-syringoyl-β-D-glucopyranoside (**8**), $[M+H]^+$ *m/z* 483

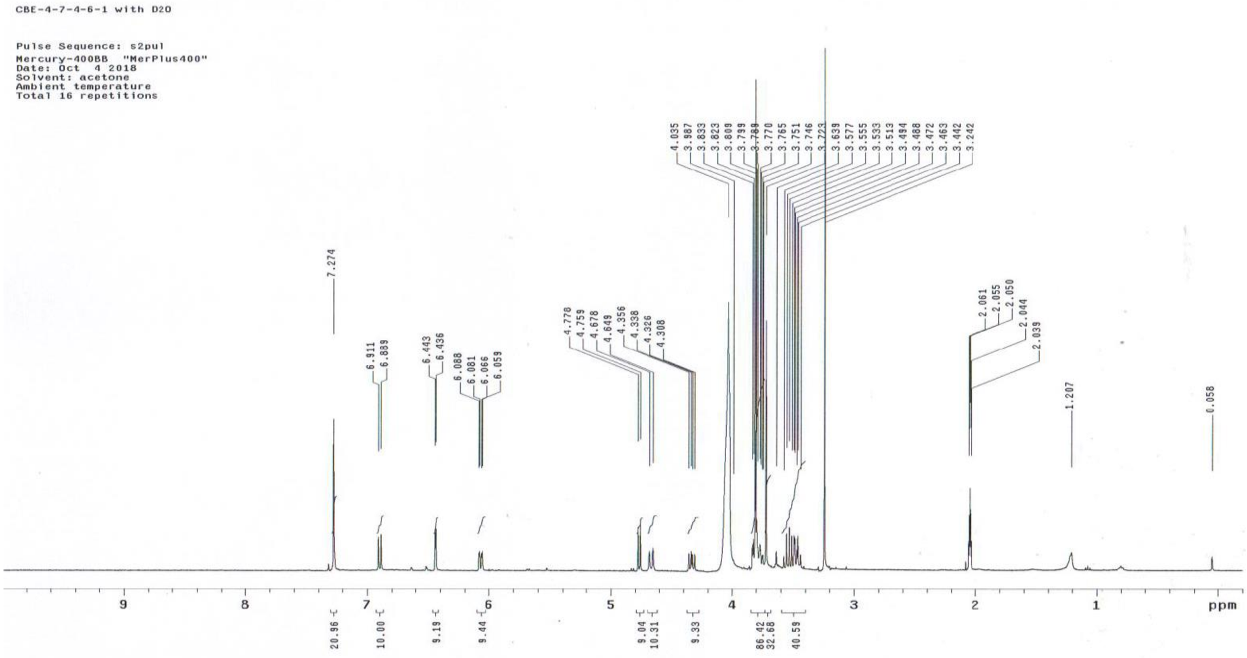

Figure S50: ^{1}H-NMR spectrum of 4-hydroxy-2-methoxyphenyl-6-*O*-syringoyl-β-D-glucopyranoside (**8**), (400 MHz, acetone-d_6/D_2O (5:1 v/v))

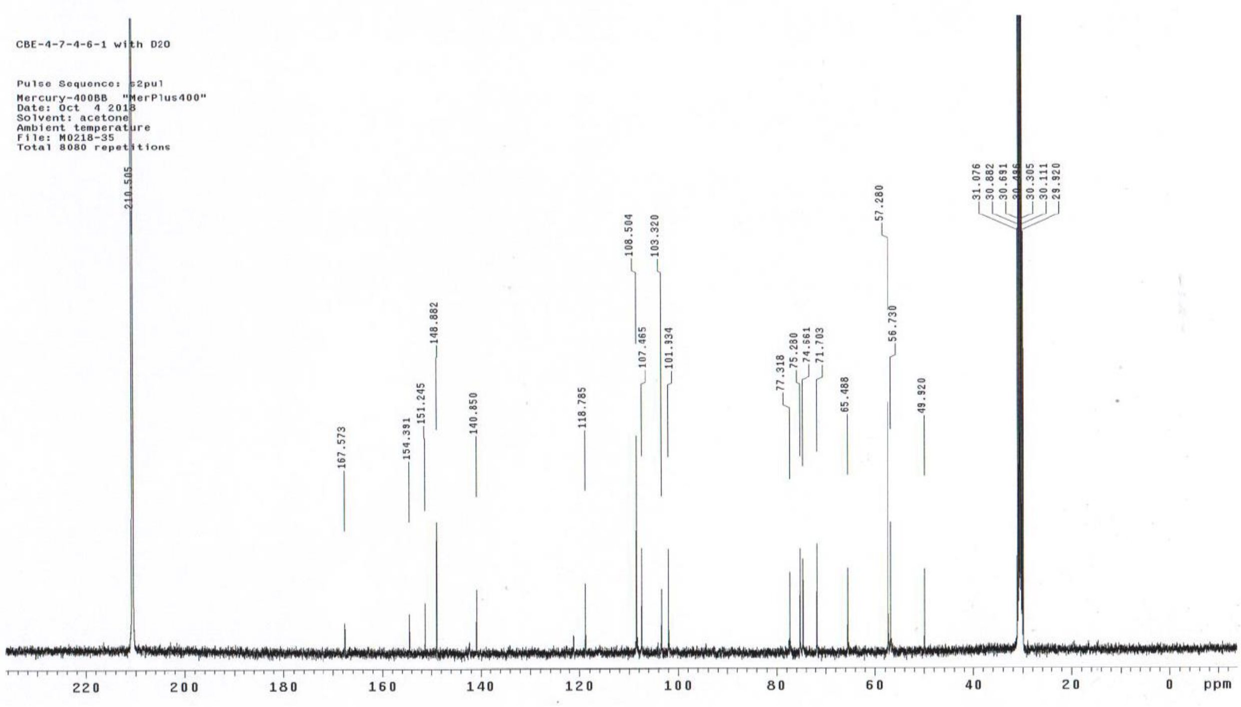

Figure S51: ^{13}C-NMR spectrum of 4-hydroxy-2-methoxyphenyl-6-*O*-syringoyl-β-D-glucopyranoside (**8**), (100 MHz, acetone-d_6/D_2O (5:1 v/v))

Reference for **8** :

Hiltunen, E., Pakkanen, T. T. & Alvila, L. Phenolic compounds in silver birch (*Betula pendula* Roth) wood. *Holzforschung* **60**, 519-527 (2006).

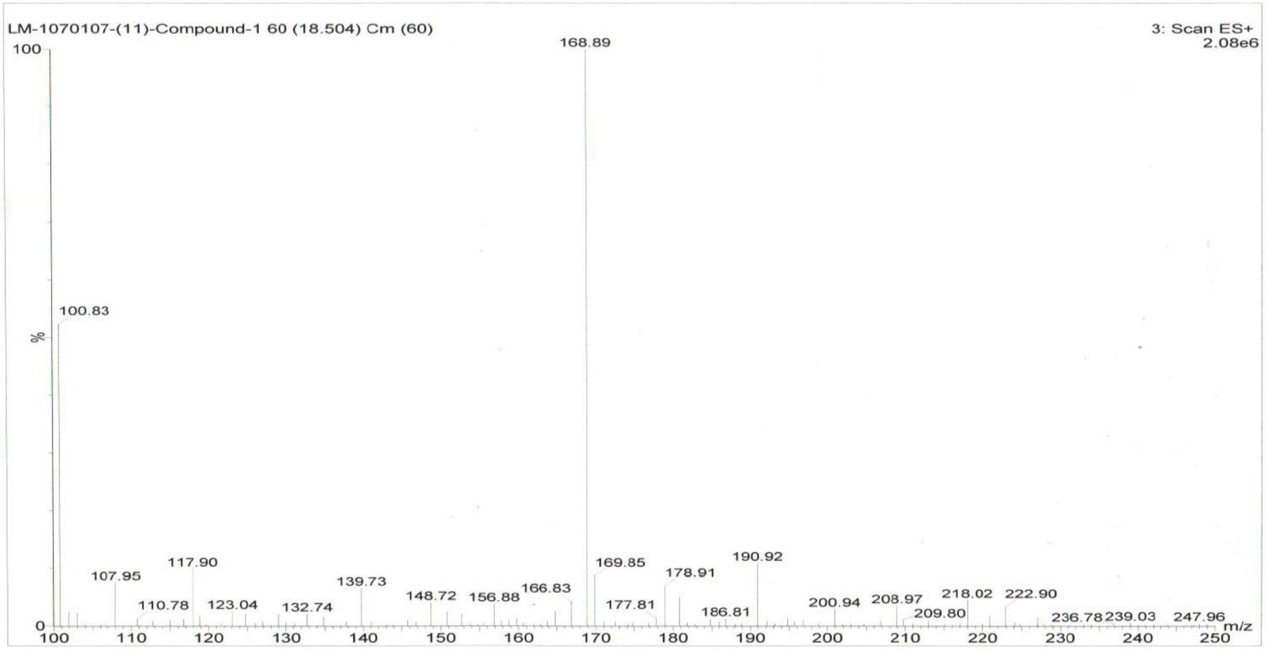

Figure S52: ESI-MS of vanillic acid (**9**), $[M+H]^+$ *m/z* 169

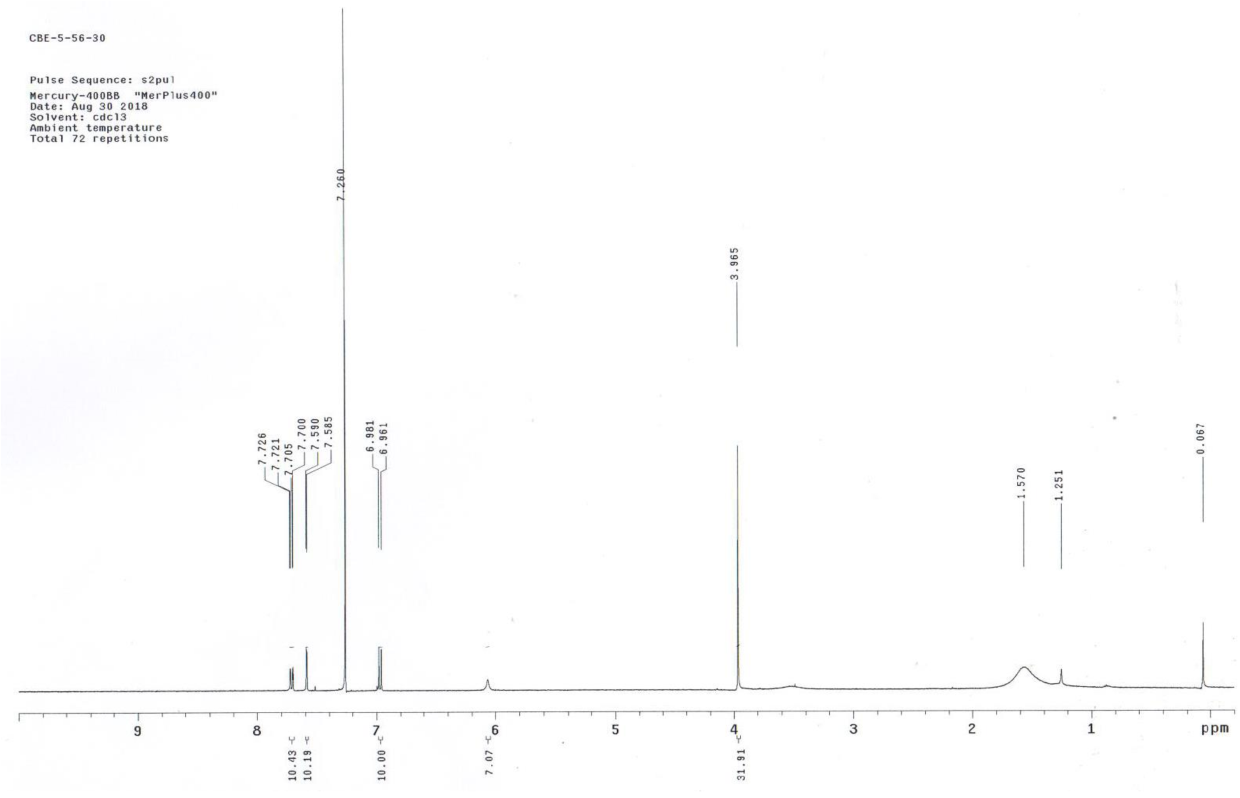

Figure S53: ^{1}H-NMR spectrum of vanillic acid (**9**), (400 MHz, $CDCl_3$)

Reference for **9** :

Chen, G. *et al.* A new flavonol from the stem-bark of *Premna fulva*. *Arkivoc* **2**, 179-185 (2010).

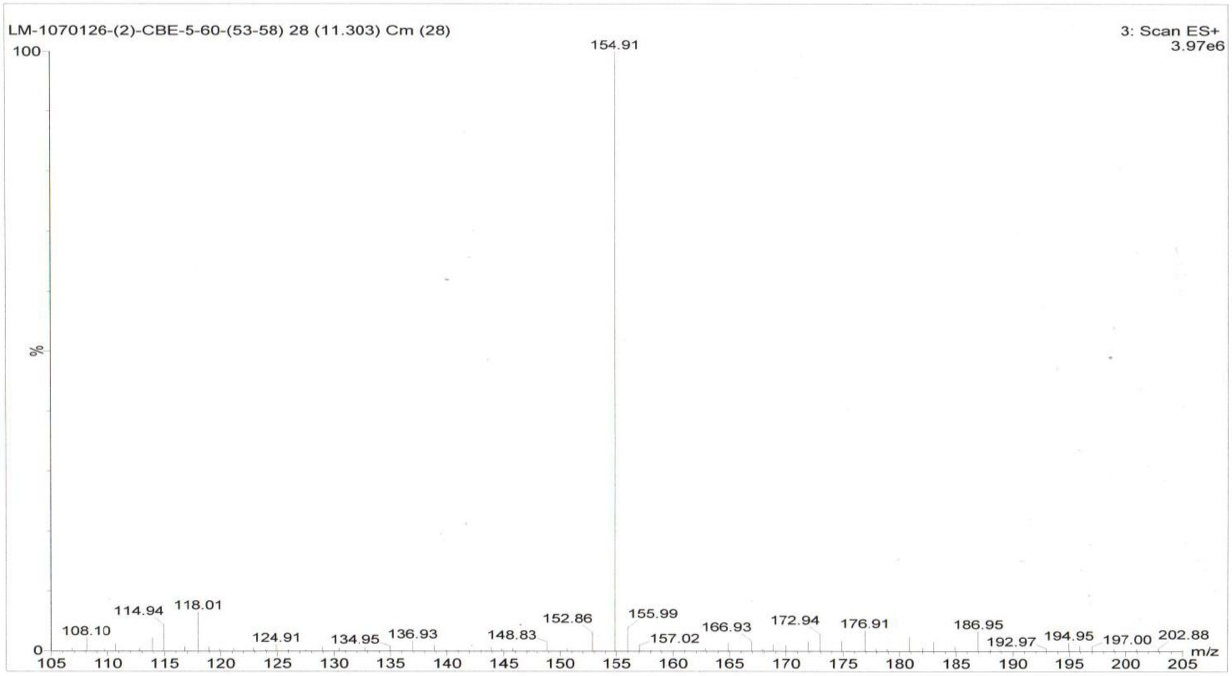

Figure S54: ESI-MS of protocatechuic acid (**10**), $[M+H]^+$ *m/z* 155

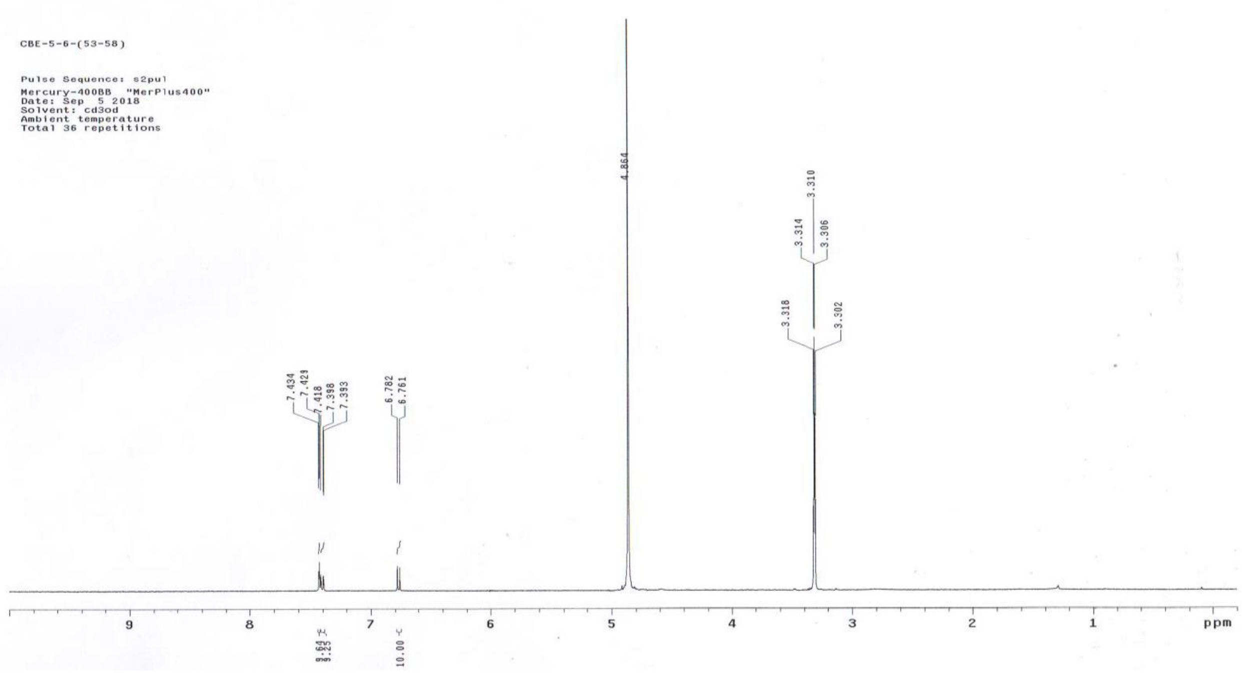

Figure S55: ^{1}H-NMR spectrum of protocatechuic acid (**10**), (400 MHz, CD_3OD)

Reference for **10** :

Rho, T. & Yoon, K. D. Chemical constituents of *Nelumbo nucifera* seeds. *Natural Product Sciences* **23**, 253-257 (2017).

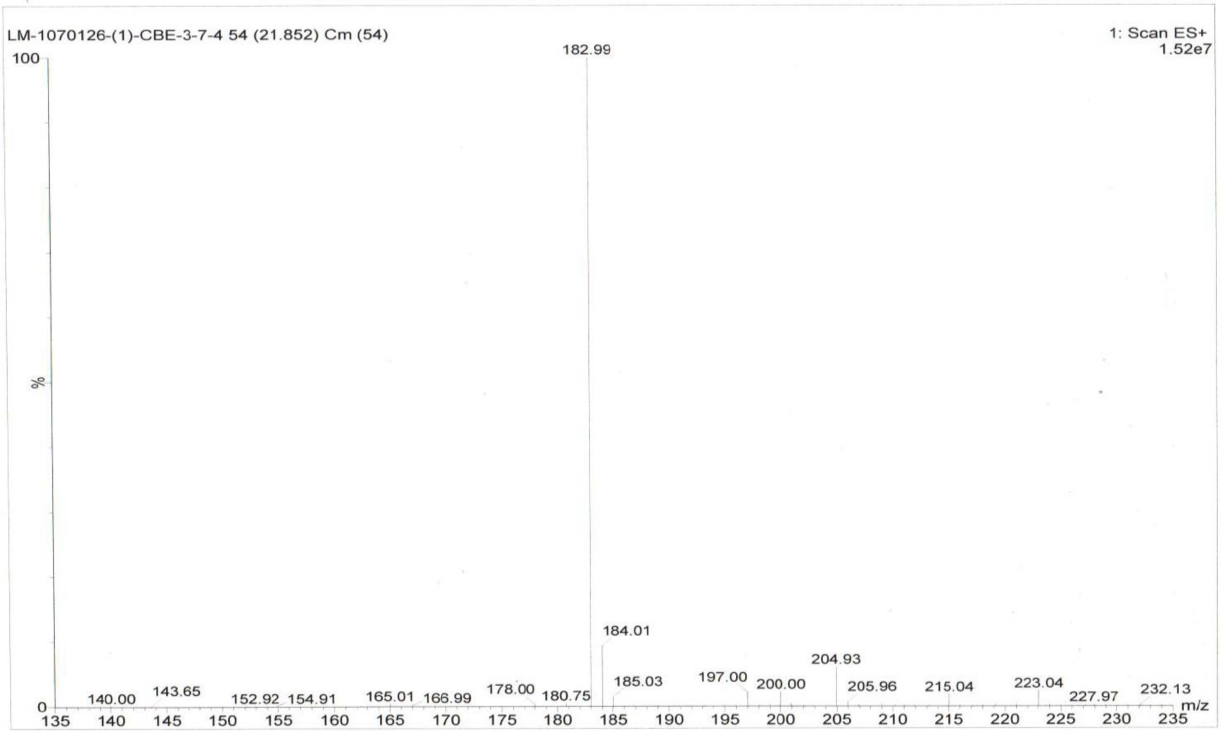

Figure S56: ESIMS of syringaldehyde (**11**), $[M+H]^+$ *m/z* 183

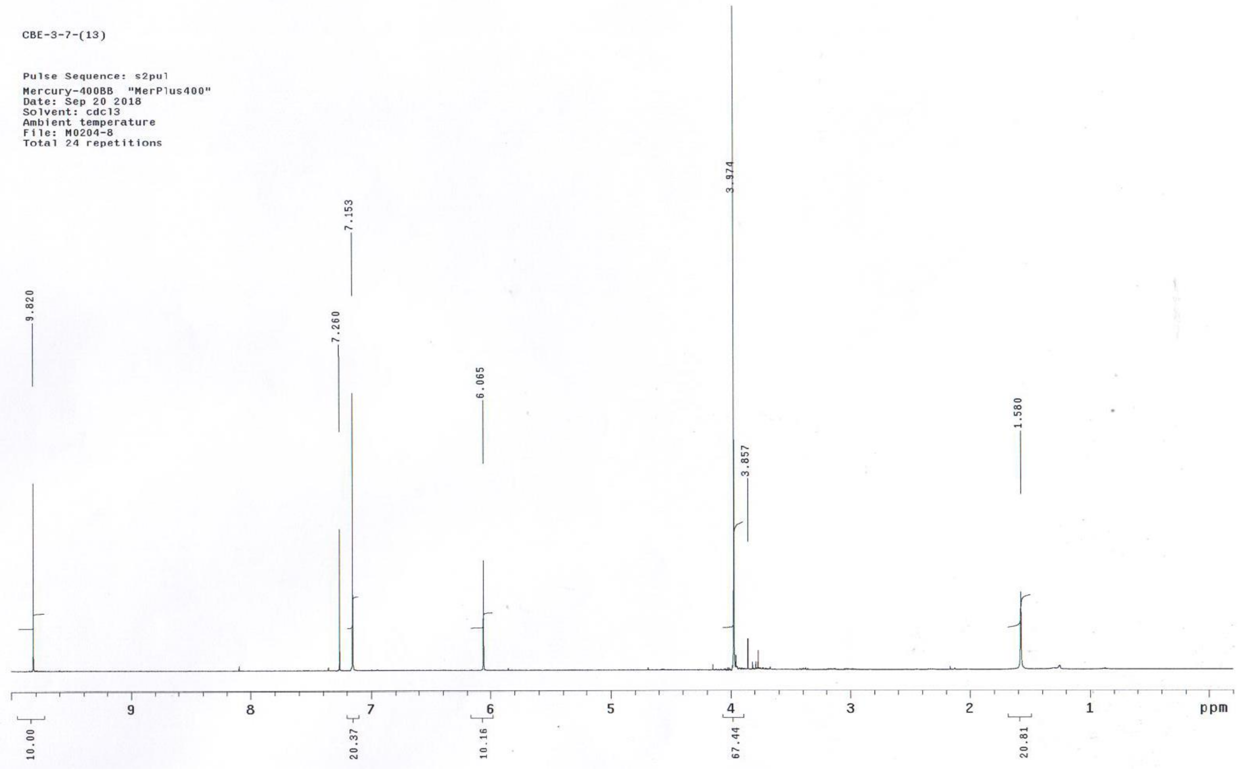

Figure S57: ^{1}H-NMR spectrum of syringaldehyde (**11**), (400 MHz, $CDCl_3$)

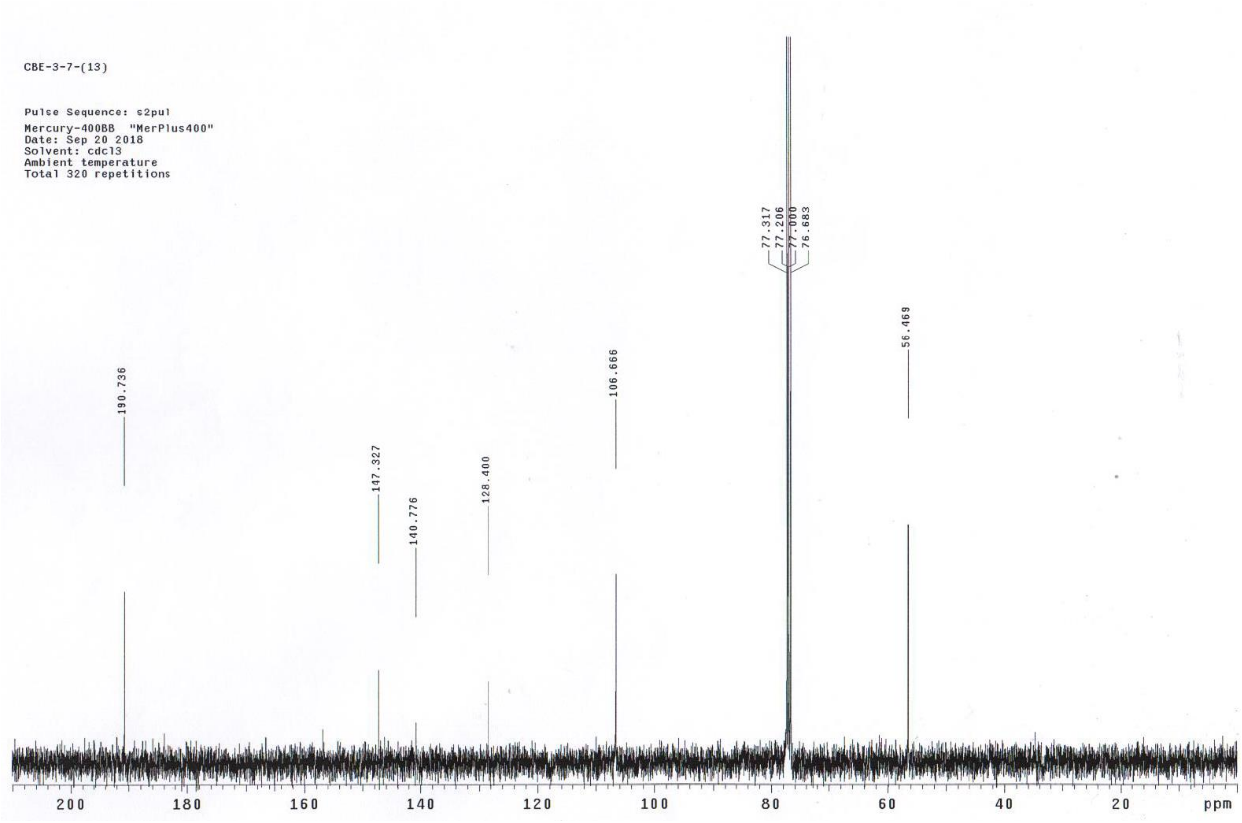

Figure S58: ^{13}C-NMR spectrum of syringaldehyde (**11**), (100 MHz, $CDCl_3$)

Reference for **11** :

Saito, T., Yamaji, T., Hayamizu, K., Yanagisawa, M. & Yamamoto, O. Spectral database for organic compounds, SDBS. https://sdbs.db.aist.go.jp/sdbs/cgi-bin/direct_frame_disp.cgi?sdbsno=5097 (1999).

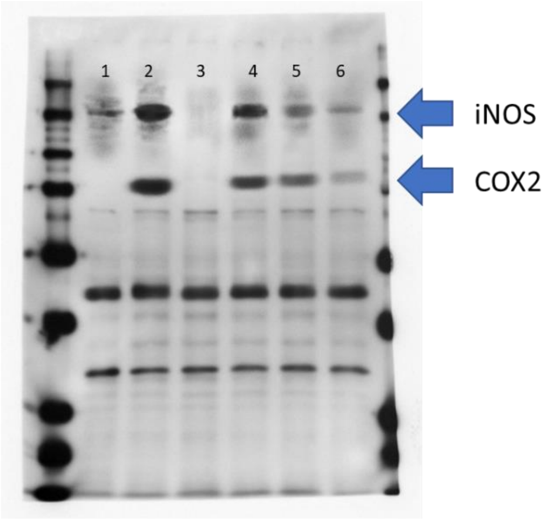

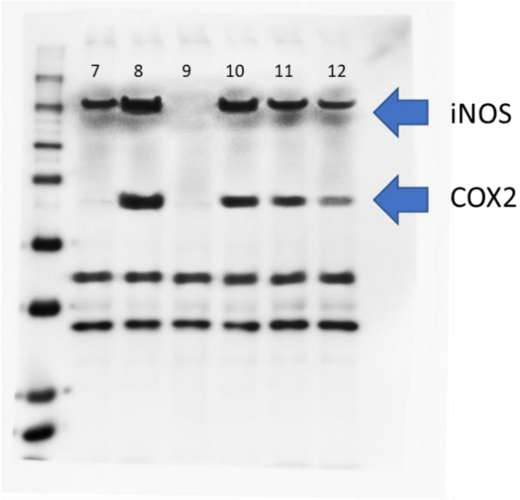

1 = DMSO
2 = DMSO + LPS
3 = 10 µM Dex + LPS
4 = 3 µM Compound 1 + LPS
5 = 10 µM Compound 1 + LPS
6 = 30 µM Compound 1 + LPS

7 = DMSO
8 = DMSO + LPS
9 = 10 µM Dex + LPS
10 = 3 µM Compound 2 + LPS
11 = 10 µM Compound 2 + LPS
12 = 30 µM Compound 2 + LPS

Figure S59: The original Western blots of presenting in **Figure 3**

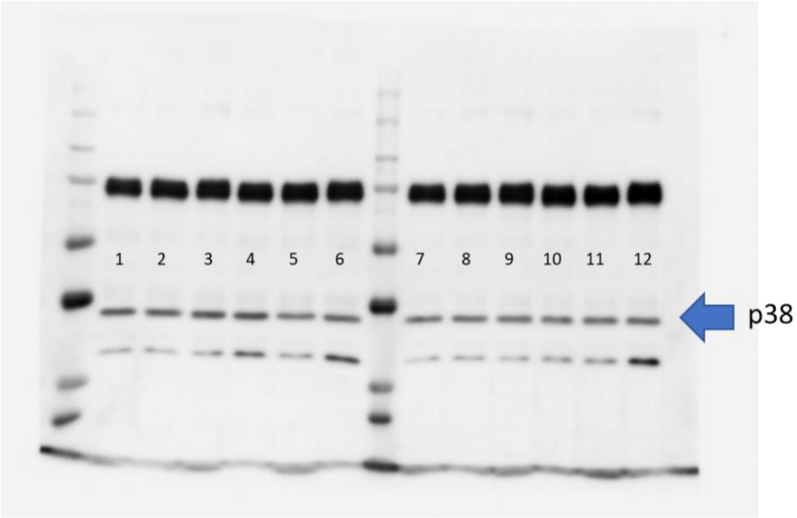

1 = DMSO
2 = DMSO + LPS
3 = 10 µM Dex + LPS
4 = 3 µM Compound 1 + LPS
5 = 10 µM Compound 1 + LPS
6 = 30 µM Compound 1 + LPS

7 = DMSO
8 = DMSO + LPS
9 = 10 µM Dex + LPS
10 = 3 µM Compound 2 + LPS
11 = 10 µM Compound 2 + LPS
12 = 30 µM Compound 2 + LPS

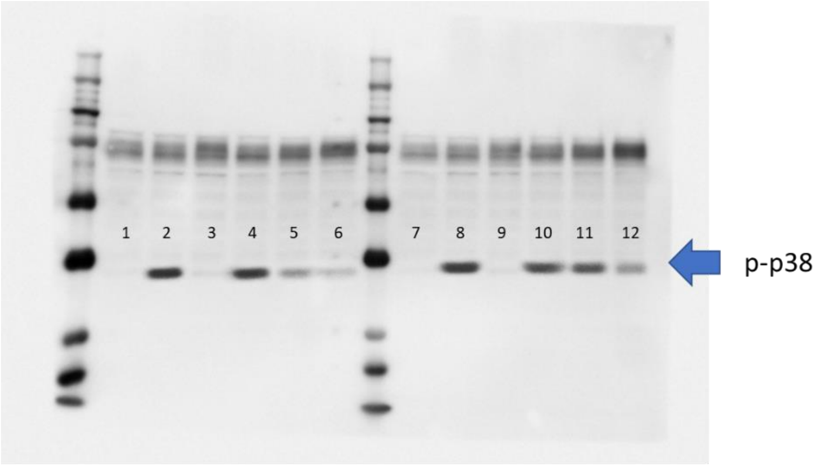

1 = DMSO
2 = DMSO + LPS
3 = 10 µM Dex + LPS
4 = 3 µM Compound 1 + LPS
5 = 10 µM Compound 1 + LPS
6 = 30 µM Compound 1 + LPS

7 = DMSO
8 = DMSO + LPS
9 = 10 µM Dex + LPS
10 = 3 µM Compound 2 + LPS
11 = 10 µM Compound 2 + LPS
12 = 30 µM Compound 2 + LPS

Figure S60: The original Western blots of presenting in **Figure 4**

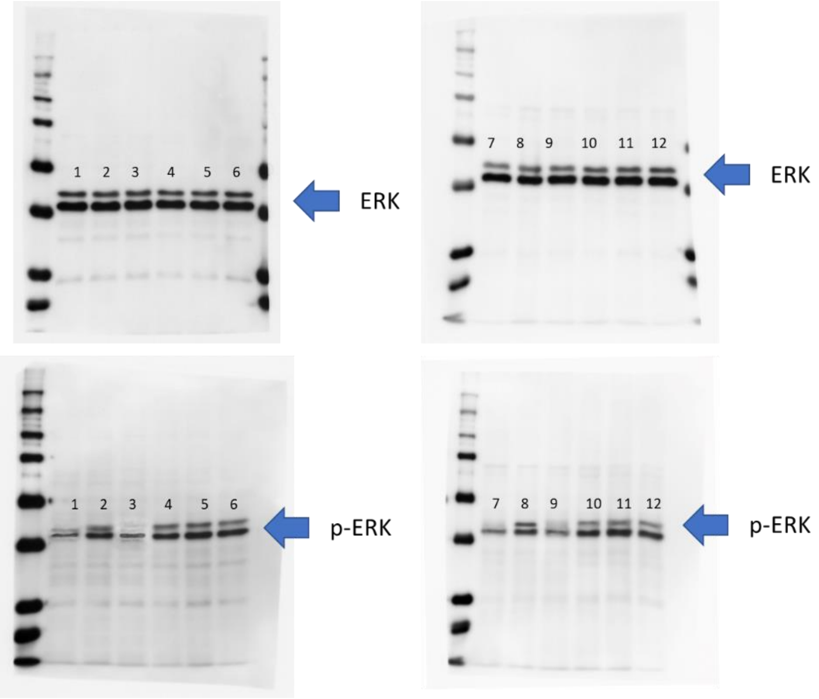

1 = DMSO
2 = DMSO + LPS
3 = 10 μM Dex + LPS
4 = 3 μM Compound 1 + LPS
5 = 10 μM Compound 1 + LPS
6 = 30 μM Compound 1 + LPS

7 = DMSO
8 = DMSO + LPS
9 = 10 μM Dex + LPS
10 = 3 μM Compound 2 + LPS
11 = 10 μM Compound 2 + LPS
12 = 30 μM Compound 2 + LPS

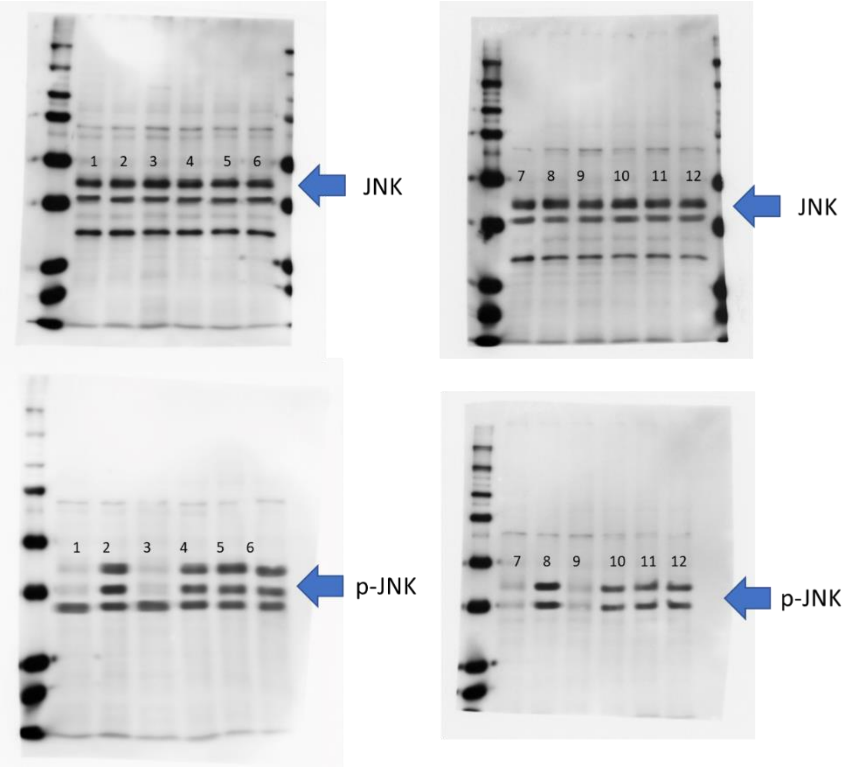

1 = DMSO
2 = DMSO + LPS
3 = 10 μM Dex + LPS
4 = 3 μM Compound 1 + LPS
5 = 10 μM Compound 1 + LPS
6 = 30 μM Compound 1 + LPS

7 = DMSO
8 = DMSO + LPS
9 = 10 μM Dex + LPS
10 = 3 μM Compound 2 + LPS
11 = 10 μM Compound 2 + LPS
12 = 30 μM Compound 2 + LPS

Figure S60 (continued): The original Western blots of presenting in **Figure 4**

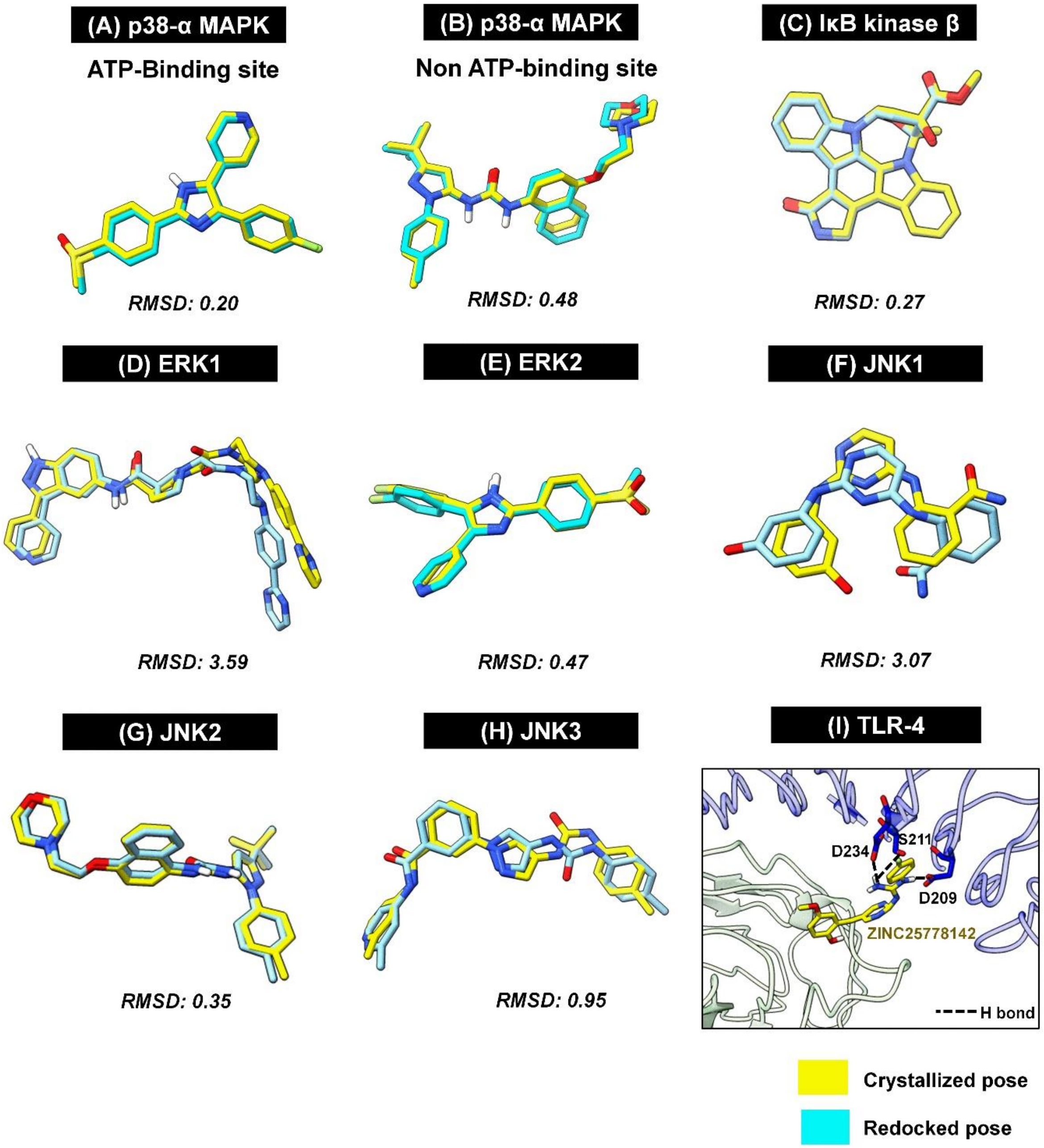

Figure S61: Validation of docking protocols used in this study. (**A-H**) Alignment of the redocked pose and available crystallized ligand for focused kinases in the molecular docking study as well as calculated RMSD value derived from superimposition of two structures (the lower RMSD indicates greater overlapping). (**I**) The orientation within the TLR4-MD2 interface of the docked conformation (ZINC25778142 compound) used as a reference and its intermolecular interactions with the key reported residues including D209, S211, and D234.

Table S2 Targeted proteins and crystallized ligand used in *in silico* studies

**Targets**	**PDB**	**Crystallized ligand**	**Reference**
TLR-4	2Z65	-	[44]
P38α (ATP-binding site)	3ZSH	SB203580	[33]
P38α (non-ATP binding site)	1KV2	BIRB796	[45]
ERK1	4QTB	SCH772984	[46]
ERK2	1PME	SB203580	[47]
JNK1	2NO3	4-anilinopyrimidines	[48]
JNK2	3NPC	BIRB796	[49]
JNK3	4W4Y	3-(4-{[(4-methylphenyl)carbamoyl]amino}-1H-pyrazol-1-yl)-N-(2-methylpyridin-4-yl)benzamide	[50]
IκB kinase β	4KIK	K-252A	[51]
